# Anesthetic Exposure During Childhood and Neurodevelopmental Outcomes: A Systematic Review and Meta-analysis

**DOI:** 10.1001/jamanetworkopen.2022.17427

**Published:** 2022-06-16

**Authors:** Charles Reighard, Shaqif Junaid, William M. Jackson, Ayesha Arif, Hannah Waddington, Andrew J. O. Whitehouse, Caleb Ing

**Affiliations:** 1 Department of Anesthesiology, Columbia University Vagelos College of Physicians and Surgeons, New York, New York; 2 Faculty of Education, Victoria University of Wellington, Wellington, New Zealand; 3 Telethon Kids Institute, University of Western Australia, Perth, Australia; 4 Department of Anesthesiology and Epidemiology, Columbia University Vagelos College of Physicians and Surgeons and Mailman School of Public Health, New York, New York

## Abstract

**Importance:**

Clinical studies of neurodevelopmental outcomes after anesthetic exposure have evaluated a range of outcomes with mixed results.

**Objective:**

To examine via meta-analyses the associations between exposure to general anesthesia and domain-specific neurodevelopmental outcomes in children.

**Data Sources:**

PubMed/MEDLINE, Embase, CINAHL, Web of Science and the Cochrane Library were searched from inception to August 31, 2021.

**Study Selection:**

Inclusion criteria were exposures to procedures requiring general anesthesia at younger than 18 years and evaluation of long-term neurodevelopmental function after exposure. Studies lacking unexposed controls or focused on children with major underlying comorbidities were excluded.

**Data Extraction and Synthesis:**

Extracted variables included effect size; hazard, risk, or odds ratio; number of exposures; procedure type; major comorbidities; age of exposure and assessment; presence of unexposed controls; and study design. Studies were independently reviewed by 2 coders, and review was conducted in accordance with the Preferred Reporting Items for Systematic Reviews and Meta-Analyses guidelines. Data were pooled using a random-effects model.

**Main Outcomes and Measures:**

The main outcomes were standardized mean differences (SMD) for scores in the neurodevelopmental domains of academics, behavioral problems, cognition, executive function, general development, language, motor function, nonverbal reasoning, social cognition, and hazard and risk of neurodevelopmental disorder diagnoses.

**Results:**

A total of 31 studies contributed data for meta-analysis. For each of the assessed neurodevelopmental domains, the numbers of children evaluated ranged from 571 to 63 315 exposed and 802 to 311 610 unexposed. Children with any exposure (single or multiple) had significantly worse behavioral problems scores, indicating more behavioral problems (SMD, −0.10; 95% CI, −0.18 to −0.02; *P* = .02), and worse scores in academics (SMD, −0.07; 95% CI −0.12 to −0.01; *P* = .02), cognition (SMD, −0.03; 95% CI, −0.05 to 0.00; *P* = .03), executive function (SMD, −0.20; 95% CI, −0.32 to −0.09; *P* < .001), general development (SMD, −0.08; 95% CI, −0.13 to −0.02; *P* = .01), language (SMD, −0.08; 95% CI, −0.14 to −0.02; *P* = .01), motor function (SMD, −0.11; 95% CI, −0.21 to −0.02; *P* = .02), and nonverbal reasoning (SMD, −0.15; 95% CI, −0.27 to −0.02; *P* = .02). Higher incidences of neurodevelopmental disorder diagnoses were also reported (hazard ratio, 1.19; 95% CI, 1.09 to 1.30; *P* < .001; risk ratio, 1.81; 95% CI, 1.25 to 2.61; *P* = .002).

**Conclusions and Relevance:**

These findings support the hypothesis that associations between anesthetic exposure during childhood and subsequent neurodevelopmental deficits differ based on neurodevelopmental domain.

## Key Points

**Question:**

Is exposure to general anesthesia during childhood associated with deficits in specific neurodevelopmental domains?

**Findings:**

In this systematic review and meta-analysis of 31 studies, childhood exposure to general anesthesia was associated with statistically significantly more behavioral problems and neurodevelopmental disorder diagnoses, as well as deficits in executive function, nonverbal reasoning, motor function, and, to a lesser extent, language, general development, and academics. Cognition score differences, while statistically significant, had the weakest association.

**Meaning:**

These findings suggest that the associations between anesthetic exposure during childhood and subsequent neurodevelopmental deficits differ based on neurodevelopmental domain.

## Teaser

This systematic review and meta-analysis assesses associations between exposure to general anesthesia and domain-specific neurodevelopmental outcomes in children.

## Introduction

Millions of children are exposed to anesthesia for surgical and diagnostic procedures each year.^1,2^ Clinical studies have evaluated neurodevelopmental outcomes after exposure to anesthesia with mixed results. Methodological variability contributes to the difficulty in interpreting these studies, including heterogeneity in patient populations (eg, underlying comorbid illnesses), doses and durations of anesthetic exposure, and even the outcomes used to evaluate children.^3^ As anesthetic neurotoxic effects were first identified in rodents,^4^ and then other animal models prior to the identification of an obvious human phenotype of injury,^5^ a wide range of outcomes have been evaluated in human studies, including academic performance, general intelligence, language, behavior, and mental disorder diagnoses.^3,6^ In some meta-analyses, early anesthetic exposure has been associated with overall neurodevelopmental impairment, but these studies pooled data from all outcome types.^7,8,9^ A 2021 meta-analysis^10^ only pooled data from the same prospectively collected neuropsychological tests, finding more behavioral problems in children with a single anesthetic exposure but no differences in general intelligence. While the restrictive criteria of that analysis by Ing et al^10^ only allowed the inclusion of 3 studies, those results suggest that statistically significant differences in children exposed to general anesthesia may be found in meta-analyses pooling data from multiple studies, and that the association between anesthesia exposure and neurodevelopmental deficit may differ based on which neurodevelopmental domain was evaluated.

This study systematically reviewed all studies of neurodevelopmental outcomes after exposure to surgical procedures and anesthesia to map reported outcomes into formal domains. A domain specific meta-analysis was then performed on studies of children without major underlying comorbidities to evaluate the hypothesis that anesthetic exposure is preferentially associated with deficits in specific neurodevelopmental domains.

## Methods

### Search Strategy

This systematic review and meta-analysis was approved by the institutional review board at Columbia University Medical Center. Systematic review with meta-analysis was performed adhering to the Preferred Reporting Items for Systematic Reviews and Meta-analyses (PRISMA) reporting guideline and Meta-analysis of Observational Studies in Epidemiology (MOOSE) reporting guideline.^11^ The review protocol was not registered in an online database.

All studies evaluating neurodevelopmental outcomes in children who were exposed to a procedure requiring general anesthesia at younger than 18 years were identified. Search algorithms were applied to PubMed/MEDLINE, Embase, CINAHL, Web of Science, and the Cochrane Library. Search algorithms were previously published by Clausen and colleagues,^3^ identifying 67 English-language studies published before June 17, 2017. In this study, the same algorithms were used, save for minor modifications in search term formatting to identify additional studies published June 17, 2017, to August 31, 2021 (eMethods in the Supplement).^10^ This method of using results from previously published systematic reviews has been advocated as a method for a more efficient review of new evidence.^12^ Reference lists of included studies were also reviewed to identify additional studies that were missed.

### Identification of All Studies of Adverse Outcomes Associated With General Anesthetic

The inclusion criteria for systematic reviews were exposure to procedures requiring general anesthesia at younger than 18 years and any evaluation of neurodevelopmental function after exposure. Exclusion criteria were nonresearch review articles, studies only involving animals, and studies measuring short term outcomes (eg, postoperative delirium, postoperative pain, and outcomes measured within 30 days of exposure). Studies that met criteria were reviewed using Covidence systematic review software (Veritas Health Innovation) with characteristics and outcomes from each study independently reviewed and extracted by 2 of the reviewers (C.R., S.J., W.M.J., A.A., and C.I.), with conflicts resolved through consensus and consultation with a third reviewer.

### Grouping Neurodevelopmental Outcomes Into Domains in All Studies

All neurodevelopmental outcomes were categorized by 2 research psychologists, with A.W. initially reviewing all outcomes and placing each into a specific neurodevelopmental domain and subdomain based on standard classifications.^13^ H.W. subsequently reviewed these domain and subdomain categories and the mapping of each outcome, with any conflicts resolved by discussion and consensus.

### Inclusion and Exclusion Criteria for Meta-analysis

Studies eligible for domain specific meta-analysis were required to include children exposed to general anesthesia, as well as an unexposed comparison group, and report outcomes from at least 1 of the psychologist-identified domains. Studies that focused primarily on children with major preexisting comorbidities or congenital anomalies were excluded. This process was adopted owing to the difficulty of determining the contribution of the anesthetic to neurodevelopment in children with significant baseline comorbidity and major perioperative physiological insult, such as children who required cardiopulmonary bypass for heart surgery.

Where duplicate reports evaluating the same population were found, only the study with the largest sample size was chosen. For studies that reported outcomes of the same cases (eg, at different ages), selection was determined by longest follow-up interval. For studies evaluating the same cohorts but reporting different outcomes (ie, scores from different domains) outcomes from all different domains were evaluated.

### Evaluation of Specific Neurodevelopmental Domains Using Meta-analysis

Given prior evidence that neurodevelopmental deficits in children exposed to general anesthesia differed by domain,^10^ meta-analyses were performed evaluating each domain independently. Most studies reported scores that reflected overall function in a given neurodevelopmental domain. Owing to the uncertain validity of comparing overall domain scores with subdomain scores within a specific domain, the overall scores were chosen for analysis. If 2 overall domain scores were available, the more comprehensive overall score was chosen based on consensus by the research psychologists (A.W. and H.W.). For language, evaluations of verbal IQ were pooled with evaluations of overall language. For academics, while most studies reported an overall score, some studies only reported individual subject scores (eg, reading, math). To include results from these studies, an overall academic score was calculated by combining math and reading scores into a synthetic score^14^ generated by mean math and reading scores and calculating variance using a correlation of 0.55.^15^ For some domains in which specific subdomains are commonly evaluated, subdomain analysis was also performed. These subdomains included internalizing and externalizing behavioral problems and fine and gross motor function.

For the clinical diagnoses and symptoms domain, outcomes like blindness were not conceptualized as neurodevelopmental disorders and were excluded. Some studies evaluated the presence of any neurodevelopmental disorder diagnoses, while others focused on specific categories. When overall neurodevelopmental disorder diagnoses were not available, learning disability was preferentially chosen since it was the primary outcome of many studies evaluating neurodevelopmental disorder diagnoses, followed by attention-deficit/hyperactivity disorder (ADHD). A subanalysis specifically evaluating ADHD was also performed.

### Statistical Analysis

To evaluate all domains on the same scale, the standardized mean differences (SMDs) between exposed and unexposed children were calculated for scores from each study. Negative SMDs indicated worse scores in the exposed group while positive SMDs indicated worse scores in the unexposed group. When studies only reported odds ratios (ORs) or risk ratios (RRs) of crossing a score threshold for deficit, the ORs or RRs were converted to SMDs.^16^ ORs were natural log–transformed, and SEs were calculated. Each natural log OR and corresponding SE were then converted to effect size and its SE by dividing by π / √3. In studies reporting RRs, RRs were first converted to ORs. For the clinical diagnoses and symptoms domain, studies reporting hazard ratios (HRs) for neurodevelopmental disorder diagnosis were analyzed separately from studies reporting RRs and ORs, which were converted to and reported as RRs.^17^ Only 1 case-control study was identified for inclusion, and given that RR could not be calculated, the data from this study could not be pooled with the other studies and was therefore excluded. Consistency between studies was evaluated using Cochrane Q and *I*^2^ statistics. An overall meta-analysis for each domain was performed by pooling data from eligible studies using random-effects models.

Domain-specific analyses assessed children with any exposure (single or multiple) compared with unexposed children. Given the potential for increased risk of Type I error when fewer than 3 studies are included,^18^ meta-analyses were only performed if data were available from at least 3 studies in a specific domain. Publication bias was evaluated using a funnel plot. Analyses were performed using RevMan version 5 (Cochrane Collaboration),^19^ and statistical significance was determined at the 2-sided *P* < .05 level for all outcomes.

The potential differences associated with the number of anesthetic exposures were explored in additional sensitivity analyses, specifically evaluating children with single exposure with possible multiple exposure, single exposure only, and multiple exposure only. The *single with possible multiple exposure* classification allowed for single exposure as well as studies designed to identify children who had a single exposure but did not exclude those who had additional exposure prior to outcome assessment. A *combined studies* classification referred to studies designed to include children with single and multiple exposures but did not report independent results from each group. Combined studies and those in which exposure number was not specified were excluded from these sensitivity analyses.

Critical appraisals were conducted using the Cochrane risk of bias tool^20^ for randomized clinical trials (RCTs) and the Risk Of Bias in Nonrandomised Studies of Interventions (ROBINS-I)^21,22^ for nonrandomized studies. Two reviewers (S.J., W.M.J., and C.I.), independently assessed each study, with conflicts resolved through consensus and consultation with a third reviewer.

## Results

### Systematic Review and Identification of Neurodevelopmental Domains

**Figure 1.**
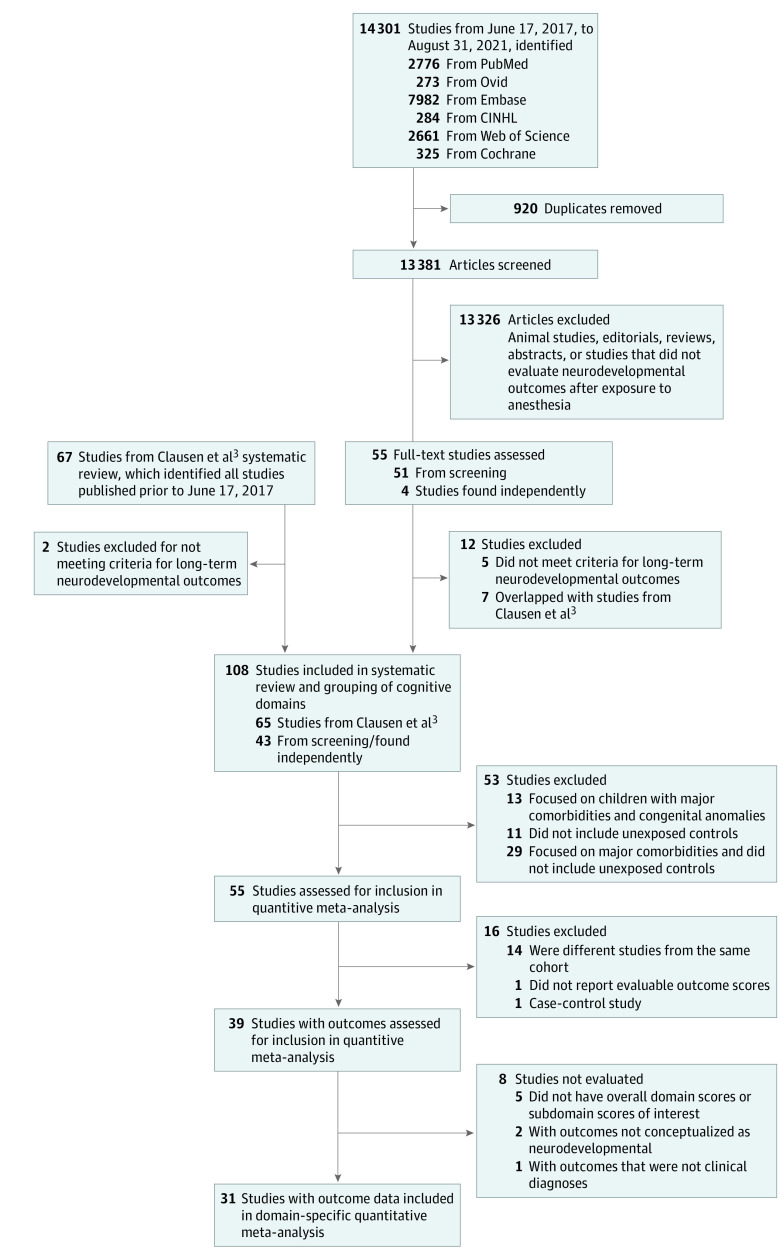
Flowchart of Included Studies

The systemic review identified 14 301 studies published between June 17, 2017, and August 31, 2021, after removal of duplicates. By combining 65 studies identified by Clausen and colleagues,^3^ and 43 studies (4 were identified by reviewing reference lists of included studies) in the present review, a total of 108 studies^23,24,25,26,27,28,29,30,31,32,33,34,35,36,37,38,39,40,41,42,43,44,45,46,47,48,49,50,51,52,53,54,55,56,57,58,59,60,61,62,63,64,65,66,67,68,69,70,71,72,73,74,75,76,77,78,79,80,81,82,83,84,85,86,87,88,89,90,91,92,93,94,95,96,97,98,99,100,101,102,103,104,105,106,107,108,109,110,111,112,113,114,115,116,117,118,119,120,121,122,123,124,125,126,127,128,129,130^ met criteria for inclusion for systematic review and grouping of cognitive domains (Figure 1). Outcomes from studies were classified into 13 domains: academics, adaptive behavior, behavioral problems, cognition, clinical diagnoses and symptoms (only neurodevelopmental disorder diagnoses were evaluated in the meta-analysis), executive function, general development, general health and well-being, language, motor function, nonverbal reasoning, sensory, and social cognition (eTable 1 in the Supplement), with some outcomes also mapping to subdomains. The assigned domains and subdomains of each of the 422 different outcomes are reported sorted by outcome type (eTable 2 in the Supplement) and sorted by domain and subdomain (eTable 3 in the Supplement), as well as the outcomes reported in each study (eTable 4 in the Supplement).

Study characteristics were also evaluated, including comorbidities and ages of exposure and assessment. Of 108 studies considered for meta-analysis, 53 studies were excluded for lacking an unexposed population or focusing on children with major comorbidities. The major comorbidities justifying exclusion for each study are described in eTable 5 in the Supplement and include congenital heart disease, extreme prematurity, or medulloblastoma. A further 16 studies were excluded for using cohorts that overlapped with other studies or not reporting outcome scores that could be evaluated (eTable 6 in the Supplement), and 8 studies were excluded for not reporting overall domain scores, leaving 31 studies^27,31,33,35,36,40,45,48,56,60,61,63,64,72,76,79,82,87,88,90,91,95,102,103,104,105,107,108,120,122,130^ contributing data for meta-analysis (Table). Owing to a limited number of eligible studies (less than 3 studies) evaluating adaptive behavior, general health and well-being, sensory, and social cognition, these domains could not be evaluated.

**Table.** All Outcomes Used From 31 Studies Included in the Domain-Specific Meta-analysis^a^

Study	Outcomes used		Outcomes not used	
	Overall	Subdomains	Outcome	Reason for not using
**Academics**				
Bartels et al,^27^ 2009	National standardized test	NA	NA	NA
Flick et al,^35^ 2011	California achievement math and reading	NA	NA	NA
Hansen et al,^36^ 2011	Test score (standardized test and teacher rating)	NA	Test nonattainment	Used test scores
Bong et al,^48^ 2013	PSLE	NA	NA	NA
Ing et al al,^56^ 2014	WAMSE numeracy and reading	NA	WAMSE spelling and writing	Used numeracy and reading outcomes
Williams et al,^61^ 2014	State achievement math and reading	NA	NA	NA
Glatz et al,^88^ 2017	School grades	NA	NA	NA
Hu et al, 2017^90^	OLSAT	NA	NA	NA
Schneuer et al,^103^ 2018	NAPLAN numeracy and reading	NA	NA	NA
Warner et al,^105^ 2018	CLDQ math and reading	NA	NA	NA
McCann et al,^108^ 2019	WIAT-II numerical and reading	NA	WIAT-II spelling	Not overall outcome
Walkden et al,^122^ 2020	Key stage 4 total points	NA	Key stage 2 English, Math, and Science; Key stage 3 English Math, and Science; Number of Key Stage 4 examination entries; Key Stage 2 nonattainment; Key Stage 3 nonattainment, Key stage 4 English and Math A, A, B, or C grade; and Key stage 4 Science 2 “good” passes (≥C grade)	Used overall outcome at oldest age
**Adaptive behavior**				
Sun et al,^82^ 2016	NA	NA	ABAS-II composite	Inadequate number of studies for meta-analysis
McCann et al,^108^ 2019	NA	NA	ABAS-II composite	Inadequate number of studies for meta-analysis
Kobayashi et al,^120^ 2020	NA	NA	J-ASQ-3 problem solving, personal-social	Not overall outcome
**Behavioral problems**				
Bartels et al,^27^ 2009	NA	CTRS-R	NA	NA
Ing et al,^40^ 2012	CBCL total problems	CBCL internalizing and externalizing	NA	NA
Stratmann et al,^60^ 2014	CBCL total problems	NA	NA	NA
Bakri et al,^64^ 2015	NA	CBCL internalizing and externalizing	CBCL aggressive behavior, anxious depression, delinquent behavior, emotionally reactive, somatic complaints, or withdrawn	Subdomains, not overall scores
Sun et al,^82^ 2016	CBCL total problems	CBCL internalizing and externalizing	NA	NA
Warner et al,^105^ 2018	CBCL total problems	CBCL internalizing and externalizing	NA	NA
Khochfe et al,^107^ 2019	NA	ECBI	NA	NA
McCann et al,^108^ 2019	CBCL total problems	CBCL internalizing and externalizing	NA	NA
Walkden et al,^122^ 2020	SDQ	NA	Skuse sociocognitive dysfunction score	Not overall score
**Cognition**				
Walker et al,^33^ 2010	BSID-III cognition	NA	NA	NA
Flick et al,^35^ 2011	TCS total cognitive	NA	NA	NA
Stratmann et al,^60^ 2014	WASI FSIQ	NA	NA	NA
Backeljauw et al,^63^ 2015	WISC-III NL	NA	NA	NA
Sun et al,^82^ 2016	WASI FSIQ	NA	NA	NA
Glatz et al,^88^ 2017	Conscription IQ	NA	NA	NA
Lv et al,^102^ 2018	BSID-II MDI	NA	NA	NA
Warner et al,^105^ 2018	WASI FSIQ	NA	NA	NA
McCann et al,^108^ 2019	WPPSI-III FSIQ	NA	NA	NA
Walkden et al,^122^ 2020	WISC-III GIQ	NA	WASI GIQ	Less comprehensive measure of cognition
Zhou et al,^130^ 2021	WPPSI-IV CR FSIQ	NA	NA	NA
**Clinical diagnoses and symptoms**				
Wilder et al,^31^ 2009	LD diagnosis	NA	Reading, written, and math LD	Used overall LD
Flick et al,^35^ 2011	LD diagnosis	NA	Reading, written, and math LD, IEP speech/language, and IEP emotion/behavior	Used overall LD
Sprung et al,^45^ 2012	ADHD diagnosis	ADHD diagnosis	NA	NA
Bong et al,^48^ 2013	LD diagnosis	NA	NA	NA
Minutillo et al,^51^ 2013	NA	NA	Blindness, cerebral palsy, and hearing	Not conceptualized as neurodevelopmental disorder
Ing et al a,^56^ 2014	Mental disorder diagnosis	NA	NA	NA
Bakri et al,^64^ 2015	ADHD diagnosis	ADHD diagnosis	Affective, anxiety, pervasive developmental, and oppositional defiant problems	Used ADHD diagnoses
Hu et al,^90^ 2017	LD diagnosis	ADHD diagnosis	Reading, written, and math LD; IEP speech/language; and IEP emotion/behavior	Used overall LD
Ing et al a,^91^ 2017	Mental disorder diagnoses	ADHD diagnosis	DD diagnosis	Used mental disorder and ADHD diagnosis
Nestor et al,^95^ 2017	Psychiatric diagnoses	NA	DD diagnosis	Used overall outcome
Castellheim et al,^99^ 2018	NA	NA	A-TAC ASD, A-TAC-LD, and A-TAC ADHD	Not clinical diagnoses
Kozanhan et al,^101^ 2018	NA	NA	Cerebral palsy	Not conceptualized as neurodevelopmental disorder
Tsai et al,^104^ 2018	ADHD diagnosis	ADHD diagnosis	NA	NA
Warner et al,^105^ 2018	NA	NA	CBCL ADHD problems	Not clinical diagnoses
McCann et al,^108^ 2019	Behavioral disorder diagnoses	NA	ADHD, ASD, and DD diagnosis; blindness; cerebral palsy; hearing	Used Behavioral disorder diagnoses. ADHD diagnosis could not be evaluated because no other studies reported ADHD using odds or risk ratios
Zhou et al,^130^ 2021	NA	NA	Cerebral palsy, hearing or vision impairment, intervention for neurodevelopmental problem, DD, and language, behavioral, or psychomotor disorder	Not conceptualized as neurodevelopmental disorder, and not formal clinical diagnoses
**Executive function**				
Flick et al,^35^ 2011	NA	NA	TCS memory	Not overall score
Ing et al,^40^ 2012	NA	NA	SDMT oral and SDMT written	Not overall scores
Fan et al,^49^ 2013	NA	NA	WPPSI-III Animal House	Not overall score
Stratmann et al,^60^ 2014	NA	NA	Recognition memory	Not overall score
Taghon et al,^70^ 2015	NA	NA	Go task	Not overall score
Aun et al,^71^ 2016	NA	NA	G-TVPS, HKLL, and WJ	Not overall scores
Poor Zamany et al,^80^ 2016	NA	NA	BDS, FDS, PVF, and SVF	Not overall scores
Sun et al,^82^ 2016	BRIEF-GEC	NA	CVLT-C, DKEFS subtests, NEPSY-II (multiple components), WISC-IV coding and digit span, CPT2 commissions, and omissions	Not overall scores
Warner et al,^105^ 2018	BRIEF-GEC	NA	WCST, WRAML-2, CPT2 (any component), DKEFS expressive language composite, category fluency, and Tower Test total	Not overall scores
McCann et al,^108^ 2019	BRIEF-GEC	NA	CMS, NEPSY-II (multiple components), WPPSI-III processing speed	Not overall scores
Warner et al,^112^ 2019	NA	NA	OTB	Not overall score
Walkden et al,^122^ 2020	NA	NA	TEA-Ch sky search and opposite worlds, and counting span	Not overall scores
Zhou et al,^130^ 2021	NA	NA	WPPSI-IV CR working memory, and processing speed	Not overall scores
**General development**				
Graham et al,^76^ 2016	EDI total score	NA	NA	NA
O’Leary et al,^79^ 2016	EDI early developmental vulnerability	NA	EDI multiple challenge index and language and cognitive development	Not overall scores
Schneuer et al,^103^ 2018	Developmentally high-risk AVEDI	NA	AVEDI cognitive development	Not overall score
**General health and well-being**				
Graham et al,^76^ 2016	NA	NA	EDI physical health and well-being	Not overall score
O’Leary et al,^79^ 2016	NA	NA	EDI physical health and well-being	Not overall score
**Language**				
Ing et al,^40^ 2012	CELF Total Score	NA	PPVT	Less comprehensive measure of language than CELF Total Score
Stratmann et al,^60^ 2014	WASI VIQ	NA	NA	NA
Backeljauw et al,^63^ 2015	WISC-III VIQ	NA	OWLS	Not overall score
Graham et al,^76^ 2016	NA	NA	EDI communication	Not overall score
O’Leary et al,^79^ 2016	NA	NA	EDI communication	Not overall score
Sun et al,^82^ 2016	WASI VIQ	NA	NEPSY-II comprehension of instructions and WASI similarities	Not overall score
Hu et al,^90^ 2017	OLSAT language	NA	IEP speech/language	Not overall score
Schneuer et al,^103^ 2018	NA	NA	AVEDI communication health and language	Not overall scores
Warner et al,^105^ 2018	Boston Naming Test	NA	CTOPP and WASI Vocabulary	Not overall score
McCann et al,^108^ 2019	WPPSI-III VIQ	NA	NA	NA
Kobayashi et al,^120^ 2020	J-ASQ-3 Communication	NA	NA	NA
Walkden et al,^122^ 2020	Children’s Communication Checklist	NA	WISC-III VIQ	NA
Zhou et al,^130^ 2021	WPPSI-IV (CR) VCI	NA	NA	NA
**Motor function**				
Walker et al,^33^ 2010	NA	BSID-III fine motor and gross motor	NA	NA
Ing et al,^40^ 2012	MAND	NA	NA	NA
Davidson et al,^72^ 2016	BSID-III motor composite	BSID-III fine motor and gross motor	NA	NA
Sun et al,^82^ 2016	NA	GPT dominant hand (fine motor)	NA	NA
Lv et al,^102^ 2018	BSID-II PDI	NA	NA	NA
Warner et al,^105^ 2018	NA	Beery Fine motor composite	Beery Buktenica visual perception, Beery Buktenica VMI, and GPT dominant and nondominant hand	Not overall scores
Kobayashi et al,^120^ 2020	NA	J-ASQ-3 fine motor and gross motor	NA	NA
Walkden et al,^122^ 2020	NA	NA	M-ABC preferred and nonpreferred hand peg, heel-to-toe walking, and and bean bag throwing	Limited assessment of fine and gross motor function
**Nonverbal reasoning**				
Ing et al,^40^ 2012	CPM	NA	NA	NA
Stratmann et al,^60^ 2014	WASI-PIQ	NA	NA	NA
Backeljauw et al,^63^ 2015	WISC-III PIQ	NA	NA	NA
Sun et al,^82^ 2016	WASI-PIQ	NA	WASI block design and WASI matrix reasoning	Not overall scores
de Heer et al,^87^ 2017	SON-R	NA	NA	NA
McCann et al,^108^ 2019	WPPSI-III PIQ	NA	NEPSY-II design copy	Not overall score
**Social cognition**				
Graham et al,^76^ 2016	NA	NA	EDI emotional health and social knowledge	Not overall scores
O’Leary et al,^79^ 2016	NA	NA	EDI emotional health and social knowledge	Not overall scores
McCann et al,^108^ 2019	NA	NA	NEPSY-II theory of mind and affect recognition	Not overall scores

Abbreviations: ABAS-II, Adaptive Behavior Assessment System–Second Edition; ADHD, attention-deficit/hyperactivity disorder; ASD, Autism Spectrum Disorder; A-TAC, Autism-Tics, ADHD, and Other Comorbidities Inventory; AVEDI, Early Development Instrument (Australia); BDS, Backward Digit Span Test; BRIEF-GEC, Behavior Rating Inventory of the Executive Functions Global Executive Composite; BSID-II, Bayley Scales of Infant Development–Second Edition; BSID-III, Bayley Scales of Infant Development–Third Edition; CBCL, Child Behavior Checklist; CELF, Clinical Evaluation of Language Fundamentals; CLDQ, Colorado Learning Difficulties Questionnaire; CMS, Children’s Memory Scale; CPM, Raven’s Colored Progressive Matrices; CPT2, Conner Continuous Performance Test II; CTOPP, Comprehensive Test of Phonological Processing; CTRS-R, Conners Teacher Rating Scale–Revised, Short Form; CVLT-C, California Verbal Learning Test–Children; DD, developmental delay; DKEFS, Delis-Kaplan Executive Function System; ECBI, Eyberg Child Behavior Inventory; EDI, Early Development Instrument; FDS, Forward Digit Span Test; FSIQ, Full Scale Intelligence Quotient; GIQ, Global Intelligence Quotient; GPT, Grooved Pegboard Test; G-TVPS, Gardner Test of Visual-Perceptual Skills Revised; HKLL, Hong Kong List Learning; IEP, Individualized Education Plan; J-ASQ-3, Ages and Stages Questionnaire 3 Japanese version; LD, learning disorder or disability; MAND, McCarren Assessment of Neuromuscular Development; MDI, Mental Development Index; NA, not applicable; NAPLAN, National Assessment Program-Literacy and Numeracy; NEPSY-II, Developmental Neuropsychological Assessment Battery–Second Edition; OLSAT, Stanford/Otis-Lennon School Ability Test; OTB, Operant Test Battery; OWLS, Oral and Written Language Scales; PDI, Psychomotor Development Index; PIQ, Performance Intelligence Quotient; PPVT, Peabody Picture Vocabulary Test; PSLE, Primary School Leaving Examination; PVF, Phonemic Verbal Fluency Test; SDMT, Symbol Digit Modality Test; SDQ, Strengths and Difficulties Questionnaire; SON-R, Hogrefe/Snijders-Oomen Nonverbal Intelligence Test–Revised; SVF, Semantic Verbal Fluency Test; TCS, Test of Cognitive Skills; TEA-Ch, Test of Everyday Attention for Children; VCI, Verbal Comprehension Index; VIQ, Verbal Intelligence Quotient; VMI, Visual-motor Integration; WAMSE, Western Australian Literacy and Numeracy Standardized Test; WASI, Wechsler Abbreviated Scale of Intelligence; WCST, Wisconsin Card Sort Test; WIAT-II, Wechsler Individual Achievement Test–Second Edition; WISC-III, Wechsler Intelligence Scale for Children–Third Edition; WISC-IV, Wechsler Intelligence Scale for Children–Fourth Edition; WJ, Woodcock–Johnson Visual Matching Test; WPPSI-III, Wechsler Preschool and Primary Scale of Intelligence–Third Edition; WPPSI-IV CR, Wechsler Preschool & Primary Scale of Intelligence–Fourth Edition, Chinese Version; WRAML-2, Wide Range Assessment of Memory and Learning, Second Edition.

^a^ No studies with a sensory outcome were eligible for meta-analysis.

### Domain-Specific Meta-analysis

**Figure 2.**
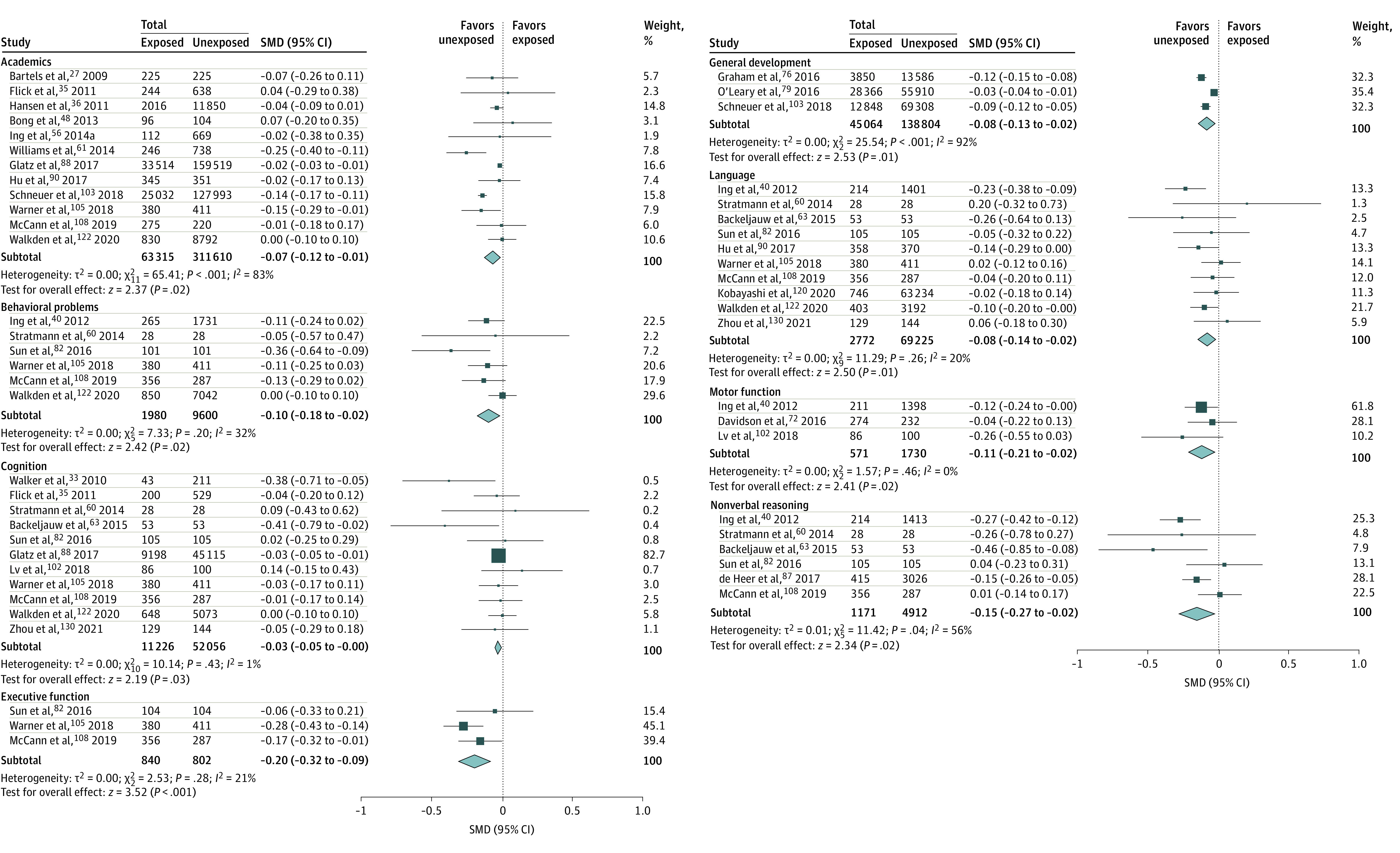
Domain-Specific Meta-analysis of Scores After Any Exposure to Surgery and Anesthesia SMD indicates standardized mean difference.

**Figure 3.**
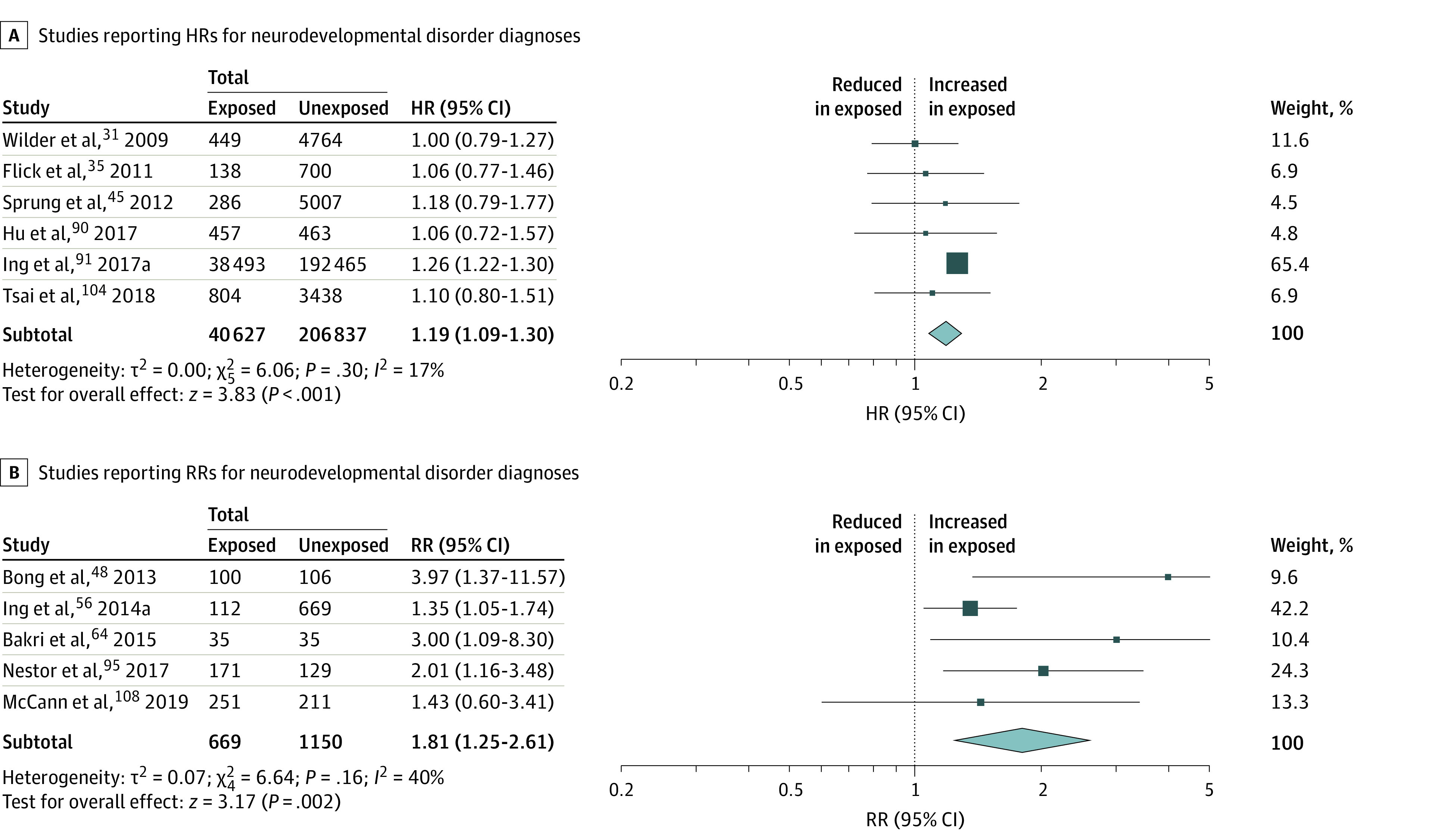
Meta-analysis of Hazard and Risk of Neurodevelopmental Disorder Diagnoses After Any Exposure to Surgery and Anesthesia HR indicates hazard ratio; RR, relative risk.

For each of the assessed neurodevelopmental domains, the numbers of children evaluated ranged from 571 to 63 315 children who were exposed and 802 to 311 610 unexposed children, depending on the domain (Figure 2). Any exposure was associated with significantly worse behavioral problems scores, indicating more behavioral problems (SMD, −0.10; 95% CI, −0.18 to −0.02; *P* = .02) and worse scores in academics (SMD, −0.07; 95% CI −0.12 to −0.01; *P* = .02), cognition (SMD, −0.03; 95% CI, −0.05 to 0.00; *P* = .03), executive function (SMD, −0.20; 95% CI, −0.32 to −0.09; *P* < .001), general development (SMD, −0.08; 95% CI, −0.13 to −0.02; *P* = .01), language (SMD, −0.08; 95% CI, −0.14 to −0.02; *P* = .01), motor function (SMD, −0.11; 95% CI, −0.21 to −0.02; *P* = .02), and nonverbal reasoning (SMD, −0.15; 95% CI, −0.27 to −0.02; *P* = .02). Any exposure was also associated with a higher incidence of neurodevelopmental disorder diagnoses (HR, 1.19; 95% CI, 1.09 to 1.30; *P* < .001; RR, 1.81; 95% CI, 1.25 to 2.61; *P* = .002) (Figure 3).

**Figure 4.**
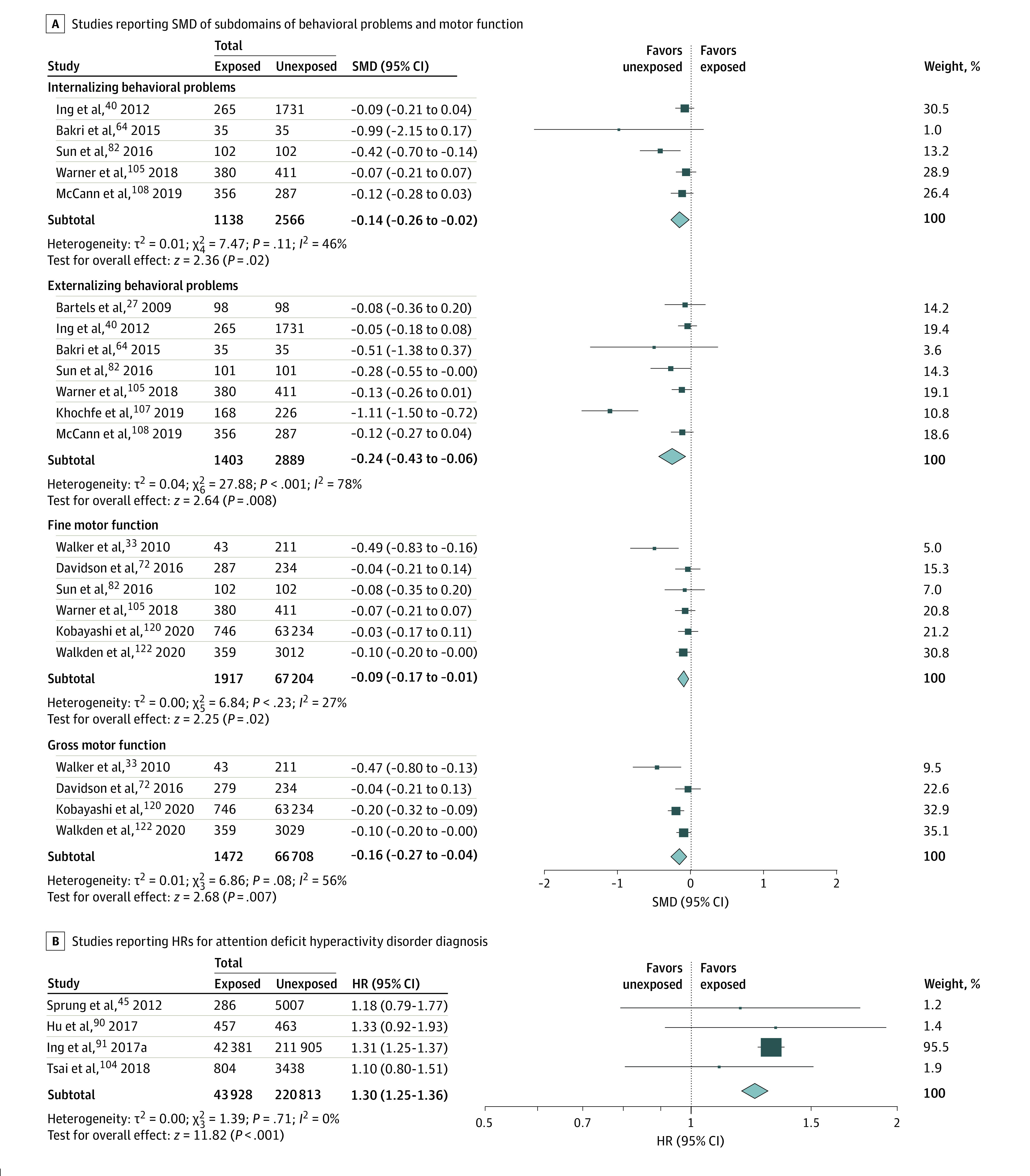
Domain-Specific Meta-analysis of Subdomain Scores and Hazard of ADHD After Any Exposure to Surgery and Anesthesia HR indicates hazard ratio; SMD, standardized mean difference.

For behavioral problem and motor function subdomains, any exposure was associated with worse internalizing (SMD, −0.14; 95% CI, −0.26 to −0.02; *P* = .02) and externalizing (SMD, −0.24; 95% CI, −0.43 to −0.06; *P* = .008) behavioral problem scores, indicating more problems, worse scores in fine (SMD, −0.09; 95% CI, −0.17 to −0.01; *P* = .02) and gross (SMD, −0.16; 95% CI, −0.27 to −0.04; *P* = .007) motor function, and a higher incidence of ADHD (HR, 1.30; 95% CI, 1.25 to 1.36; *P* < .001) (Figure 4). *I*^2^ statistics ranged from 0% to 92%, depending on the domain, indicating low between-study inconsistency in some outcomes, such as cognition and motor function domains and the ADHD diagnosis subdomain, but considerable variability in others, such as the academics and general development domains.

Sensitivity analyses evaluated the association between exposure number and domain-specific outcomes. Not all studies reported results based on exposure number, and some studies only evaluated 1 exposure type (eg, single or multiple) (eTable 7 in the Supplement). Therefore in exposure number analyses, some domains did not have an adequate number of studies to be evaluated, and in general, fewer domains were evaluated in each of the sensitivity analyses than the any exposure analysis.

In children with single with possible multiple exposure, worse scores were observed in academics (SMD, −0.09; 95% CI, −0.18 to −0.01; *P* = .03), cognition (SMD, −0.03; 95% CI, −0.05 to −0.01; *P* = .01), executive function (SMD, −0.20; 95% CI, −0.32 to −0.09; *P* < .001), and general development (SMD, −0.07; 95% CI, −0.14 to −0.01; *P* = .02), but no differences in behavioral problems, language, or nonverbal reasoning (eFigure 1 in the Supplement). A higher incidence of neurodevelopmental disorder diagnoses was also observed (HR, 1.15; 95% CI, 1.03 to 1.29; *P* = .01; RR, 2.07; 95% CI, 1.32 to 3.24; *P* = .001) (eFigure 2 in the Supplement), worse externalizing behavioral problems (SMD, −0.35; 95% CI, −0.65 to −0.06; *P* = .02), fine (SMD, −0.09; 95% CI, −0.17 to −0.01; *P* = .02) and gross (SMD, −0.16; 95% CI, −0.27 to −0.04; *P* = .007) motor function scores, and a higher incidence of ADHD diagnoses (HR, 1.30; 95% CI, 1.25 to 1.36; *P* < .001) but no difference in internalizing behavioral problems (eFigure 3 in the Supplement).

In children with single exposures, worse scores were observed in cognition (SMD, −0.03; 95% CI, −0.05 to 0.00; *P* = .02), general development (SMD, −0.07; 95% CI, −0.14 to −0.01; *P* = .02), and fine motor function (SMD, −0.08; 95% CI, −0.15 to −0.01; *P* = .03). There were no differences in academics or language, but we observed an increased incidence of neurodevelopmental disorder diagnoses (HR, 1.15; 95% CI, 1.03 to 1.29; *P* = .01) and ADHD diagnosis (HR, 1.30; 95% CI, 1.25 to 1.36; *P* < .001) (eFigure 4 and eFigure 5 in the Supplement).

In children with multiple exposures, worse scores were reported in academics (SMD, −0.16; 95% CI, −0.27 to −0.05; *P* = .006), general development (SMD, −0.08; 95% CI, −0.16 to −0.01; *P* = .04), language (SMD, −0.27; 95% CI, −0.45 to −0.09; *P* = .003), and fine motor function (SMD, −0.33; 95% CI, −0.44 to −0.23; *P* < .001). There was also an increased incidence of neurodevelopmental disorder diagnoses (HR, 1.86; 95% CI, 1.48 to 2.32; *P* < .001) and ADHD diagnosis (HR, 2.09; 95% CI, 1.53 to 2.86; *P* < .001). However, no difference in cognition was observed (eFigure 6 and eFigure 7 in the Supplement).

### Bias Assessment and Publication Bias

All studies were at risk of bias, with the highest risks due to confounding, the retrospective determination of intervention status, and presence of cointervention (eg, surgical procedure) with anesthesia exposure (eTable 8, eTable 9, and eFigure 8 in the Supplement). Examination of the funnel plot did not find bias but identified 1 outlier reporting an externalizing behavioral problems outcome from Khochfe et al^107^ (eFigure 9 in the Supplement). After removing this study, statistically significant differences in externalizing behavioral problems after any exposure persisted (SMD, −0.11; 95% CI, −0.18 to −0.03; *P* = .03), with an *I*^2^ statistic of 0%, and after single with possible multiple exposure (SMD, −0.14; 95% CI, −0.24 to −0.04; *P* = .004), with an *I*^2^ statistic of 0%.

## Discussion

In this systematic review and meta-analysis, significant differences were observed in some, but not all, assessed neurodevelopmental domains and subdomains. However, as statistical significance is based in large part on sample size, which varied between domains, a more informative way to interpret this data may be to evaluate the SMD values in addition to the *P* values.^131^ Cohen proposed that for interpreting the magnitude of SMD values in the behavioral sciences, an SMD of 0.2 suggests small effect size; 0.5, medium effect size; and 0.8, large effect sizes.^132^ Based on this interpretation, all outcomes in children with any exposure were associated with no more than small effect sizes. However, domain-specific effect size differences can be appreciated with the largest differences found in behavioral problems, executive function, nonverbal reasoning, and motor function, followed by language, general development, and academics. The smallest effect sizes were in cognition, which was measured primarily using full-scale IQ. Of these, in sensitivity analyses, differences persisted in academics, cognition, and executive function in single with possible multiple exposure, although several domains could not be evaluated owing to an inadequate number of available studies. Regarding multiple exposures, compared with unexposed children, statistically significant differences in academics and language were seen in SMDs that were approximately 2-fold as large as those found in any exposure, single with possible multiple exposure, or single exposure. No differences were found in cognition after multiple exposure.

A higher incidence of neurodevelopmental disorder diagnosis was found in children with any exposure, including a 30% increased hazard of ADHD, with differences persisting even when evaluating single with possible multiple exposure and single exposure. Compared with unexposed children, in children with multiple exposures*,* incidences of neurodevelopmental disorder diagnoses and ADHD were more than 2-fold as high as in children with any exposure.

Most studies included in these meta-analyses were observational, so anesthetic exposure cannot be causally linked to differences in scores and neurodevelopmental disorder diagnoses using our results. However, these results help define a pattern of deficit to explore in future studies. Diffuse distributions of deficits, such as those we observed in this study, have been reported in studies of other neurotoxic exposures, with the possibility of some clustering around certain domains.^133,134^ In particular, in this study, the outcome with the largest SMD was the subdomain of externalizing behavioral problems, which are commonly found in children with ADHD.^135^ The increased rates of neurodevelopmental disorder and ADHD diagnoses is also consistent with studies of other neurotoxic exposures, specifically, children exposed to pesticides have reported behavioral problems, with 50% to 142% increased risks of ADHD and symptoms of hyperactivity.^136,137^

### Limitations

This study has a number of important limitations. First, despite grouping outcomes into domains, there was significant variation in study characteristics, including medical disease in children and adjustment for confounding, as well as some differences in the outcomes combined within domains, which may have contributed to significant between-study heterogeneity, with large *I*^2^ statistics seen in several domains. Second, nearly all included studies were observational and therefore likely subject to unmeasured confounding, including underlying medical issues, perioperative physiological disturbances, or psychological trauma due to hospitalization. However, the purpose of this study was not to establish a causal relationship between anesthetic exposure and a given neurodevelopmental domain, but to suggest appropriate neurodevelopmental domains to evaluate in future studies. Third, neurodevelopmental disorders in the clinical diagnoses and symptoms domain are inherently related to scores in other domains (eg, learning disability diagnosis is related to academic scores). Fourth, the analyses were performed without *P* value adjustment for multiple comparisons. As a result, we are primarily interpreting the calculated effect sizes and CIs of the outcomes to evaluate domain-specific differences and inform the design of future studies.

## Conclusions

The results from this systematic review and meta-analysis help identify patterns of deficits in specific domains, with comparatively larger effect sizes seen in the executive function, nonverbal reasoning, motor function, and behavioral problems, particularly externalizing behavioral problems, domains, coupled with an increased incidence of neurodevelopmental disorder diagnoses, particularly ADHD diagnosis. However, the cognition domain was found to have the weakest association with anesthetic exposure. The effect sizes in children with multiple exposures were also found to be 2-fold as large as those with single exposures. Many of the individual published studies may not have adequate power to identify small effect size differences. By using meta-analyses to pool data from individual studies, potential phenotypes of neurodevelopmental deficit that are associated with general anesthesia exposure have been identified. Based on these results, further studies are needed to determine the mechanisms behind these reported associations and whether these differences in specific neurodevelopmental domains can be attributed to childhood exposure to anesthetic medications.

## Notes

Supplement. **eMethods.** Literature Search Strategies **eTable 1.** All Domains and Subdomains Evaluated in Studies of Potential Neurotoxic Effects of Anesthetic **eTable 2.** All Outcomes Evaluated in Studies of Potential Neurotoxic Effects of Anesthetic and Their Associated Neurodevelopmental Domain and Subdomain Classifications **eTable 3.** All Neurodevelopmental Domain and Subdomains and the Classification of Outcomes into These Domains and Subdomains **eTable 4.** Outcomes From Each of the 108 Reviewed Studies **eTable 5.** Characteristics of All 108 Reviewed Studies **eTable 6.** Outcomes of Duplicate Studies or Studies That Did Not Report Outcome Scores That Could Be Evaluated **eTable 7.** Exposure Data Used From Each of the 31 Included Studies **eTable 8.** Cochrane Risk of Bias Assessment in Randomized Trial **eTable 9.** Risk of Bias In Nonrandomized Studies or Interventions (ROBINS-I) Assessment of Eligible Nonrandomized Studies **eFigure 1.** Domain-Specific Meta-analysis of Scores After Single With Possible Multiple Exposure to Surgery and Anesthesia **eFigure 2.** Meta-analysis of Hazard and Risk of Clinical Diagnoses and Symptoms After Single With Possible Multiple Exposure to Surgery and Anesthesia **eFigure 3.** Domain-Specific Meta-analysis of Subdomain Scores and Hazard of ADHD After Single With Possible Multiple Exposure to Surgery and Anesthesia **eFigure 4.** Domain-Specific Meta-analysis of Scores and Hazard of Clinical Diagnoses and Symptoms After Single Exposure to Surgery and Anesthesia **eFigure 5.** Domain-Specific Meta-analysis of Subdomain Scores and Hazard of ADHD After Single Exposure to Surgery and Anesthesia **eFigure 6.** Domain-Specific Meta-analysis of Scores and Hazard of Clinical Diagnoses and Symptoms After Multiple Exposure to Surgery and Anesthesia **eFigure 7.** Domain-Specific Meta-analysis of Subdomain Scores and Hazard of ADHD After Multiple Exposure to Surgery and Anesthesia **eFigure 8.** ROBINS-I Risk of Bias Assessment Figure **eFigure 9.** Funnel Plot for Studies of Any Exposure to Surgery and Anesthesia
