# Supplementary material for: Anesthetic Exposure During Childhood and Neurodevelopmental Outcomes: A Systematic Review and Meta-analysis
Source: JAMA Netw Open. 2022 Jun 16;5(6):e2217427. doi: 10.1001/jamanetworkopen.2022.17427 (PMC9204549; doi:10.1001/jamanetworkopen.2022.17427)
Supplement: Supplement. — eMethods. Literature Search Strategies eTable 1. All Domains and Subdomains Evaluated in Studies of Potential Neurotoxic Effects of Anesthetic eTable 2. All Outcomes Evaluated in Studies of Potential Neurotoxic Effects of Anesthetic and Their Associated Neurodevelopmental Domain and Subdomain Classifications eTable 3. All Neurodevelopmental Domain and Subdomains and the Classification of Outcomes into These Domains and Subdomains eTable 4. Outcomes From Each of the 108 Reviewed Studies eTable 5. Characteristics of All 108 Reviewed Studies eTable 6. Outcomes of Duplicate Studies or Studies That Did Not Report Outcome Scores That Could Be Evaluated eTable 7. Exposure Data Used From Each of the 31 Included Studies eTable 8. Cochrane Risk of Bias Assessment in Randomized Trial eTable 9. Risk of Bias In Nonrandomized Studies or Interventions (ROBINS-I) Assessment of Eligible Nonrandomized Studies eFigure 1. Domain-Specific Meta-analysis of Scores After Single With Possible Multiple Exposure to Surgery and Anesthesia eFigure 2. Meta-analysis of Hazard and Risk of Clinical Diagnoses and Symptoms After Single With Possible Multiple Exposure to Surgery and Anesthesia eFigure 3. Domain-Specific Meta-analysis of Subdomain Scores and Hazard of ADHD After Single With Possible Multiple Exposure to Surgery and Anesthesia eFigure 4. Domain-Specific Meta-analysis of Scores and Hazard of Clinical Diagnoses and Symptoms After Single Exposure to Surgery and Anesthesia eFigure 5. Domain-Specific Meta-analysis of Subdomain Scores and Hazard of ADHD After Single Exposure to Surgery and Anesthesia eFigure 6. Domain-Specific Meta-analysis of Scores and Hazard of Clinical Diagnoses and Symptoms After Multiple Exposure to Surgery and Anesthesia eFigure 7. Domain-Specific Meta-analysis of Subdomain Scores and Hazard of ADHD After Multiple Exposure to Surgery and Anesthesia eFigure 8. ROBINS-I Risk of Bias Assessment Figure eFigure 9. Funnel Plot for Studies of Any Exposure to Surgery and Anest [file jamanetwopen-e2217427-s001.pdf]

Supplemental Online Content

Reighard C, Junaid S, Jackson WM, et al. Anesthetic exposure during childhood and neurodevelopmental outcomes: a systematic review and meta-analysis. *JAMA Netw Open*. 2022;5(6):e2217427. doi:10.1001/jamanetworkopen.2022.17427

eMethods. Literature Search Strategies
eTable 1. All Domains and Subdomains Evaluated in Studies of Potential Neurotoxic Effects of Anesthetic
eTable 2. All Outcomes Evaluated in Studies of Potential Neurotoxic Effects of Anesthetic and Their Associated Neurodevelopmental Domain and Subdomain Classifications
eTable 3. All Neurodevelopmental Domain and Subdomains and the Classification of Outcomes into These Domains and Subdomains
eTable 4. Outcomes From Each of the 108 Reviewed Studies
eTable 5. Characteristics of All 108 Reviewed Studies
eTable 6. Outcomes of Duplicate Studies or Studies That Did Not Report Outcome Scores That Could Be Evaluated
eTable 7. Exposure Data Used From Each of the 31 Included Studies
eTable 8. Cochrane Risk of Bias Assessment in Randomized Trial
eTable 9. Risk of Bias In Nonrandomized Studies or Interventions (ROBINS-I) Assessment of Eligible Nonrandomized Studies
eFigure 1. Domain-Specific Meta-analysis of Scores After Single With Possible Multiple Exposure to Surgery and Anesthesia
eFigure 2. Meta-analysis of Hazard and Risk of Clinical Diagnoses and Symptoms After Single With Possible Multiple Exposure to Surgery and Anesthesia
eFigure 3. Domain-Specific Meta-analysis of Subdomain Scores and Hazard of ADHD After Single With Possible Multiple Exposure to Surgery and Anesthesia
eFigure 4. Domain-Specific Meta-analysis of Scores and Hazard of Clinical Diagnoses and Symptoms After Single Exposure to Surgery and Anesthesia
eFigure 5. Domain-Specific Meta-analysis of Subdomain Scores and Hazard of ADHD After Single Exposure to Surgery and Anesthesia
eFigure 6. Domain-Specific Meta-analysis of Scores and Hazard of Clinical Diagnoses and Symptoms After Multiple Exposure to Surgery and Anesthesia
eFigure 7. Domain-Specific Meta-analysis of Subdomain Scores and Hazard of ADHD After Multiple Exposure to Surgery and Anesthesia
eFigure 8. ROBINS-I Risk of Bias Assessment Figure
eFigure 9. Funnel Plot for Studies of Any Exposure to Surgery and Anesthesia

This supplemental material has been provided by the authors to give readers additional information about their work.

© 2022 Reighard C et al. *JAMA Network Open.*

eMethods. Literature Search Strategies

Pubmed

(((((child OR children OR childhood OR infant OR infants OR infancy OR adolescent OR adolescents OR adolescence OR newborn OR Newborns OR neonate OR neonates OR babies OR toddlers OR paediatric OR pediatric)) AND ((Anesthesia OR Anaesthesia OR Anesthetics OR Anaesthetics OR General Anesthesia OR General Anaesthesia OR General Anesthetics OR General Anaesthetics OR Anesthetic OR Anaesthetic OR Anesthetic exposure OR Anaesthetic exposure OR Anesthetic agents OR Anaesthetic agents OR intravenous anesthesia OR intravenous anaesthesia OR intravenous anaesthetics OR intravenous anesthetics OR volatile anaesthetics OR volatile anesthetics OR inhalation anesthesia OR inhalation anaesthesia OR sevoflurane OR isoflurane OR desflurane OR nitrous oxide OR halothane OR propofol OR thiomebumal OR ketamine OR GABA-A receptor agonist OR NMDA-receptor antagonist OR Adrenergic Alpha-2 receptor agonist OR benzodiazepine OR midazolam OR clonidine OR dexmedetomidine))) AND ((Cognition OR Cognitive outcome OR Cognitive outcomes OR Cognitive defect OR Cognitive defects OR Cognitive deficit OR Cognitive deficits OR Cognitive disorder OR Cognition disorders OR Cognitive impairment OR Cognitive impairments OR Neurodevelopment OR Neurodevelopmental outcome OR Neurodevelopmental outcomes OR child behaviour OR child behaviours OR Developmental disability OR Developmental disabilities OR Developmental disorder OR Developmental disorders OR Developmental delay disorder OR Developmental delay disorders OR behavioural impairment OR behavioural impairments OR behavioural change OR behavioural changes)))) NOT ((animals[mh] NOT humans[mh]))

© 2022 Reighard C et al. *JAMA Network Open.*

OVID Medline

1. (child of impaired parents or children or single parent child or child, preschool or child,preschool or pre-school child or pre-school going children or pre-schooler or pre-schoolers or preschool child institution or preschooler or infancy or Infant or Baby or Childhood or child,school or school boy or school children or school girl or school-going boy or school-going boys or school-going child or school-going children or school-going girl or school-going girls or schoolboy or schoolboys or schoolchild or schoolchildren or schoolgirl or schoolgirls or schoolgoing child or schoolgoing children or animals, newborn or child,newborn or full term infant or human neonate or human newborn or infant, newborn or neonatal animal or neonate or neonate animal or neonates or newborn animal or newborn baby or newborn child or newborn infant or newly born baby or newly born child or newly born infant or Toddlers or adolescent, institutionalized or institutionalised adolescent or institutionalized adolescent or teenager or Teenage).mp. [mp=title, abstract, original title, name of substance word, subject heading word, floating sub-heading word, keyword heading word, organism supplementary concept word, protocol supplementary concept word, rare disease supplementary concept word, unique identifier, synonyms]
2. exp Child/
3. exp Preschool Child/
4. exp Infant/
5. exp Newborn/
6. exp Adolescent/
7. exp Adolescence/
8. 1 or 2 or 3 or 4 or 5 or 6 or 7
9. exp General an?esthesia/
10. exp Intravenous an?esthesia/
11. exp Inhalation An?esthesia/
12. exp anesthetic agent/
13. exp Isoflurane/
14. exp Sevoflurane/
15. exp Desflurane/
16. exp Halothane/
17. exp Nitrous Oxide/
18. exp Propofol/

© 2022 Reighard C et al. *JAMA Network Open.*

19. exp Thiopental/

20. exp Ketamine/

21. exp Clonidine/

22. exp Benzodiazepine/

23. exp Dexmedetomidine/

24. (Anaesthesia or anaesthesia, intratracheal or anaesthesia, auto or anaesthetic action or anesthesia, intratracheal or anesthesia,auto or anesthetic action or anesthetization or animal anaesthesia or animal anesthesia or autoanaesthesia or autoanesthesia or drop mask anaesthesia or drop mask anesthesia or narcosis or neuroanaesthesia or neuroanesthesia or short anaesthesia or short anesthesia or short duration or anaesthesia or short duration anesthesia or anaesthesia, general or anaesthesia,general or anesthesia, general or anesthesia,general or general anaesthesia or anaesthesia,child or anaesthesia,paediatric or anesthesia,child or anesthesia,pediatric or child anaesthesia or child anesthesia or paediatric anaesthesia or anaesthesia,intravenous or anaesthesia,local,intravenous or anesthesia, intravenous or anesthesia,intravenous or anesthesia,local,intravenous or intravenous anaesthesia or intravenous local anaesthesia or intravenous local anesthesia or local anaesthesia,intravenous or local anesthesia,intravenous or anaesthesia, closed-circuit or anaesthesia, inhalation or anaesthesia,closed circuit or anaesthesia,inhalation or anaesthetic system,closed or anesthesia, closed-circuit or anesthesia, inhalation or anesthesia,closed circuit or anesthesia,inhalation or anesthetic system,closed or closed anaesthetic circuit or closed anaesthetic system or closed anesthetic circuit or closed anesthetic system or closed circuit anaesthesia or closed circuit anesthesia or inhalation anaesthesia or anaesthetic or anaesthetic agent or anaesthetic drug or anaesthetics or anaesthetics, combined or anaesthetics, dissociative or anaesthetics, general or anesthetic or anesthetic drug or anesthetics or anesthetics, combined or anesthetics, dissociative or anesthetics, general or general anaesthetic or general anaesthetic agent or general anesthetic or general anesthetic agent or preanaesthetics or preanesthetics or anaesthetic,volatile or anaesthetics, inhalation or anaesthetics,rapidly evapourating or anesthetic,volatile or anesthetics, inhalation or anesthetics,rapidly evaporating or gas anaesthetic agent or gas anesthetic agent or inhalation anaesthetic or inhalation anaesthetic agent or inhalation anesthetic or volatile anaesthetic or volatile anaesthetic agent or volatile anesthetic or volatile anesthetic agent or anaesthetic,intravenous or anaesthetics, intravenous or anaesthetics,intravenous or anesthetic,intravenous or anesthetics, intravenous or anesthetics,intravenous or intravenous anaesthetic agent or aerane or aerrane or forane or forene or forthane or isoflurano or isorane or sofloran or sevoflo or sevofrane or sevorane or sevotec or sojourn or ultane or ultane pen or sulorane or suprane or anestane or bromochlorotrifluorethane or fluorothan or fluorothane or fluothane or ftorotan or halan or halothan or halothane metabolite or halothane or ineltano or narcotan or phthorothane or trothane or dinitrogen monoxide or dinitrogen oxide or factitious air or hyponitrous acid anhydride or laughing gas or nitrogen protoxide or anepol or cryotol

or diisoprofol or diprivan or diprofol or disoprivan or disoprofol or fresofol or gobbifol or pofol or propocam or propofol lipuro or propofol-lipuro or rapinovet or recofol or safol or anesthal or farmotal or hypnostan or intraval or leopental or nesdonal or penthiobarbital or penthotal or pentothal or pentothal sodico or pentothal sodium or pharmothal or ravonal or sodipental or sodium pentothal or sodium thiopental or thiomebumal or thiomebumal sodium or thionembutal or thionyl or thiopental sodium or thiopentalbarbital or thiopentemal or thiopenthal or thiopentobarbital or thiopentone or thiopentone sodium or thiotal or thiothal or tio pentemal or tiopental sodico or trapanal or anesject or calipsol or calypso or imalgene or kalipsol or katamine or keta-hameln or ketaject or ketalar or ketalin or ketamax or ketamine hcl or ketamine hydrochloride or ketaminol vet or ketanest or ketased or ketaset or ketaved or ketavet or ketmin or ketoject or ketolar or narkamon or narketan or soon-soon or tekam or velonarcon or vetalar or buccolam or dalam or doricum or dormonid or fortanest or fulsed or hypnoval or hypnovel or hypnoyvel or ipnovel or midacum or midazo or midazol or midazolam hydrochloride or midazolam hydrochloride preservative free or midolam or miloz or versed or benzodiazepine or benzodiazepin derivative or benzodiazepines or benzodiazepinones or benzodiazepine agonist or benzodiazepine receptor agonist or benzodiazepine receptor stimulant or benzodiazepine receptor stimulator or benzodiazepine stimulant or benzodiazepine stimulating agent or benzodiazepine stimulator or arkamin or atensina or caprysin or catapres or catapres tts or catapresan or catapresan depot or catapresan tts or catapressan or catapressan perlonguettes or catapressant or catasan or chlofazolin or chlophazolin or chlophelin or clonidine or clofelin or clofeline or clomidine or clonidine or clonicel or clonidine or clonidine chlorhydrate or clonidine hydrochloride or clonipresan or clonistada or clonistada retard or clonnirit or clophelin or clopheline or daipres or dcai or dichlorophenylaminoimidazoline or dichlorophenylaminoimidazoline hydrochloride or dixarit or duraclon or haemiton or hemiton or huma-clonidine or hypodine or isoglaucon or jenloga or kapvay or melzin or normopresan or normopresin or paracefan or sulmidine or taitecin or tenso timelets or adrenergic alpha agonists or adrenergic alpha-agonists or alpha adrenergic agent or alpha adrenergic agonist or alpha adrenergic receptor agent or alpha adrenergic receptor agonist or alpha adrenergic receptor stimulant or alpha adrenergic receptor stimulator or alpha adrenergic stimulant or alpha adrenergic stimulating agent or alpha adrenergic stimulator or alpha adrenoceptor agonist or alpha adrenoceptor stimulant or alpha adrenoceptor stimulating agent or alpha adrenoceptor stimulator or alpha agonist or alpha sympathicomimetic or alpha sympathicomimetic agent or noradrenalin agonist or noradrenergic agonist or noradrenergic receptor stimulating agent or dexamedetomidine or dexdomitor or dexdor or dexmedetomidine hydrochloride or precede or primadex or sileo or GABA agonist or GABA agonists or GABA receptor agonist or GABA receptor stimulant or GABA receptor stimulating agent or GABA receptor stimulator or GABAergic receptor agonist or GABAergic receptor stimulant or GABAergic receptor stimulating agent or GABAergic receptor stimulator or GABAmimetic or GABAmimetic agent or gamma aminobutyric acid agonist or gamma aminobutyric acid receptor agonist or gamma aminobutyric acid receptor stimulant or gamma aminobutyric acid receptor stimulating agent or gamma aminobutyric acid receptor stimulator or n-

© 2022 Reighard C et al. *JAMA Network Open.*

methyl d-aspartate antagonist or n-methyl d-aspartate blocker or n-methyl d-aspartate blocking agent or n-methyl d-aspartate receptor antagonist or n-methyl d-aspartate receptor blocker or n-methyl d-aspartate receptor blocking agent or n-methyl d-aspartic acid antagonist or n-methyl d-aspartic acid blocker or n-methyl d-aspartic acid blocking agent or n-methyl d-aspartic acid receptor antagonist or n-methyl d-aspartic acid receptor blocker or n-methyl d-aspartic acid receptor blocking agent or n-methyl dextro aspartate antagonist or n-methyl dextro aspartate blocker or n-methyl dextro aspartate blocking agent or n-methyl dextro aspartate receptor antagonist or n-methyl dextro aspartate receptor blocker or n-methyl dextro aspartate receptor blocking agent or n-methyl dextro aspartic acid antagonist or n-methyl dextro aspartic acid blocker or n-methyl dextro aspartic acid blocking agent or n-methyl dextro aspartic acid receptor antagonist or n-methyl dextro aspartic acid receptor blocker or NMDA antagonist or NMDA blocker or NMDA blocking agent or NMDA receptor antagonist or NMDA receptor antagonists or NMDA receptor blocker or NMDA receptor blocking agent).mp. [mp=title, abstract, original title, name of substance word, subject heading word, floating sub-heading word, keyword heading word, organism supplementary concept word, protocol supplementary concept word, rare disease supplementary concept word, unique identifier, synonyms]

25. 9 or 10 or 11 or 12 or 13 or 14 or 14 or 15 or 16 or 17 or 18 or 19 or 20 or 21 or 22 or 23 or 24

26. (cognitive accessibility or cognitive balance or cognitive dissonance or cognitive function or cognitive structure or cognitive symptoms or cognitive task or cognitive thinking or neurobehavioral manifestations or neurobehavioural manifestations or volition or cognition disorder or cognition disorders or cognitive defects or cognitive deficit or cognitive disability or cognitive disorder or cognitive disorders or cognitive dysfunction or cognitive impairment or delirium, dementia, amnestic, cognitive disorders or overinclusion or response interference or behavior, child or behaviour, child or child behavior or infant behavior or infant behavior or disorder,learning or impaired learning or learning deficit or learning difficulty or learning disabilities or learning disability or learning disorders or learning disturbance or learning impairment or learning problem or abnormal development or child development disorder or development disorder or developmental disabilities).mp. [mp=title, abstract, original title, name of substance word, subject heading word, floating sub-heading word, keyword heading word, organism supplementary concept word, protocol supplementary concept word, rare disease supplementary concept word, unique identifier, synonyms]

27. exp developmental disorder/ or exp Learning disorder/ or exp Child behavior/ or exp Postoperative cognitive dysfunction/ or exp Cognitive defect/ or exp Cognition assessment/ or exp Cognition/

26 or 27

28. 8 and 25 and 28

29 not ((exp animal/ or nonhuman/) not exp human/)

© 2022 Reighard C et al. *JAMA Network Open.*

Embase

1. 'child of impaired parents'/exp OR 'child of impaired parents' OR (('child'/exp OR child) AND of AND impaired AND ('parents'/exp OR parents)) OR 'children'/exp OR children OR 'single parent child'/exp OR 'single parent child' OR (single AND ('parent'/exp OR parent) AND ('child'/exp OR child)) OR 'child, preschool'/exp OR 'child, preschool' OR (child, AND ('preschool'/exp OR preschool)) OR 'pre-school child'/exp OR 'pre-school child' OR ('pre school' AND ('child'/exp OR child)) OR 'pre-school going children'/exp OR 'pre-school going children' OR ('pre school' AND going AND ('children'/exp OR children)) OR 'pre schooler'/exp OR 'pre schooler' OR 'pre schoolers'/exp OR 'pre schoolers' OR 'preschool child institution'/exp OR 'preschool child institution' OR (('preschool'/exp OR preschool) AND ('child'/exp OR child) AND institution) OR 'preschooler'/exp OR preschooler OR 'infancy'/exp OR infancy OR 'infant'/exp OR infant OR 'baby'/exp OR baby OR 'childhood'/exp OR childhood OR 'child,school'/exp OR child,school OR 'school boy'/exp OR 'school boy' OR (('school'/exp OR school) AND ('boy'/exp OR boy)) OR 'school children'/exp OR 'school children' OR (('school'/exp OR school) AND ('children'/exp OR children)) OR 'school girl'/exp OR 'school girl' OR (('school'/exp OR school) AND ('girl'/exp OR girl)) OR 'school-going boy' OR ('school going' AND ('boy'/exp OR boy)) OR 'school-going boys' OR ('school going' AND boys) OR 'school-going child' OR ('school going' AND ('child'/exp OR child)) OR 'school-going children' OR ('school going' AND ('children'/exp OR children)) OR 'school-going girl' OR ('school going' AND ('girl'/exp OR girl)) OR 'school-going girls' OR ('school going' AND girls) OR 'schoolboy'/exp OR schoolboy OR 'schoolboys'/exp OR schoolboys OR 'schoolchild'/exp OR schoolchild OR 'schoolchildren'/exp OR schoolchildren OR 'schoolgirl'/exp OR schoolgirl OR 'schoolgirls'/exp OR schoolgirls OR 'schoolgoing child' OR (schoolgoing AND ('child'/exp OR child)) OR 'schoolgoing children' OR (schoolgoing AND ('children'/exp OR children)) OR 'animals, newborn'/exp OR 'animals, newborn' OR (animals, AND ('newborn'/exp OR newborn)) OR 'child, newborn'/exp OR 'child, newborn' OR (child, AND ('newborn'/exp OR newborn)) OR 'full term infant'/exp OR 'full term infant' OR (full AND term AND ('infant'/exp OR infant)) OR 'human neonate'/exp OR 'human neonate' OR (('human'/exp OR human) AND ('neonate'/exp OR neonate)) OR 'human newborn'/exp OR 'human newborn' OR (('human'/exp OR human) AND ('newborn'/exp OR newborn)) OR 'infant, newborn'/exp OR 'infant, newborn' OR (infant, AND ('newborn'/exp OR newborn)) OR 'neonatal animal'/exp OR 'neonatal animal' OR (neonatal AND ('animal'/exp OR animal)) OR 'neonate'/exp OR neonate OR 'neonate animal'/exp OR 'neonate animal' OR (('neonate'/exp OR neonate) AND ('animal'/exp OR animal)) OR neonates OR 'newborn animal'/exp OR 'newborn animal' OR (('newborn'/exp OR newborn) AND ('animal'/exp OR animal)) OR 'newborn baby'/exp OR 'newborn baby' OR (('newborn'/exp OR newborn) AND ('baby'/exp OR baby)) OR 'newborn child'/exp OR 'newborn child' OR (('newborn'/exp OR newborn) AND ('child'/exp OR child)) OR 'newborn infant'/exp OR 'newborn infant' OR (('newborn'/exp OR newborn) AND ('infant'/exp OR infant)) OR 'newly born baby'/exp OR 'newly born baby' OR (newly AND born AND ('baby'/exp OR baby)) OR 'newly born child'/exp OR 'newly born child' OR (newly AND born AND ('child'/exp OR child)) OR 'newly born infant'/exp OR 'newly born infant' OR (newly AND born AND ('infant'/exp OR infant)) OR 'toddlers'/exp OR toddlers OR 'adolescent,

© 2022 Reighard C et al. *JAMA Network Open.*

institutionalized'/exp OR 'adolescent, institutionalized' OR (adolescent, AND institutionalized) OR 'institutionalised adolescent'/exp OR 'institutionalised adolescent' OR (institutionalised AND ('adolescent'/exp OR adolescent)) OR 'institutionalized adolescent'/exp OR 'institutionalized adolescent' OR (institutionalized AND ('adolescent'/exp OR adolescent)) OR 'teenager'/exp OR teenager OR 'teenage'/exp OR teenage

2. 'Child'/exp

3. 'Preschool Child'/exp

4. 'Juvenile'/exp

5. 'Infancy'/exp

6. 'Infancy'/exp

7. 'Baby'/exp

8. 'Childhood'/exp

9. 'School Child'/exp

10. 'Newborn'/exp

11. 'Toddler'/exp

12. 'Adolescent'/exp

13. 'Adolescence'/exp

14. 1 or 2 or 3 or 4 or 5 or 6 or 7 or 8 or 9 or 10 or 11 or 12 or 13

15. 'General an?esthesia'/exp

16. 'P?ediatric An?esthesia'/exp

17. 'Intravenous an?esthesia'/exp

18. 'Inhalation An?esthesia'/exp

19. 'anesthetic agent'/exp

20. 'Inhalation an?esthetic Agent'/exp

21. 'Intravenous an?esthetic Agent'/exp

22. 'Isoflurane'/exp

23. 'Sevoflurane'/exp

24. 'Desflurane'/exp

25. 'Halothane'/exp

26. 'Nitrous Oxide'/exp

© 2022 Reighard C et al. *JAMA Network Open.*

27. 'Propofol'/exp

28. 'Thiopental'/exp

29. 'Ketamine'/exp

30. 'Clonidine'/exp

31. 'Benzodiazepine receptor stimulating agent'/exp

32. 'Benzodiazepine derivative'/exp

33. 'Benzodiazepine'/exp

34. 'alpha adrenergic receptor stimulating agent'/exp

35. 'Dexmedetomidine'/exp

36. '4 aminobutyric acid receptor stimulating agent'/exp

37. 'n methyl dextro aspartic acid receptor blocking agent'/exp

38. 'anaesthesia, intratracheal'/exp OR 'anaesthesia, intratracheal' OR (anaesthesia, AND intratracheal) OR 'anaesthesia,auto'/exp OR anaesthesia,auto OR 'anaesthetic action'/exp OR 'anaesthetic action' OR (('anaesthetic'/exp OR anaesthetic) AND ('action'/exp OR action)) OR 'anesthesia, intratracheal'/exp OR 'anesthesia, intratracheal' OR (anesthesia, AND intratracheal) OR 'anesthesia,auto'/exp OR anesthesia,auto OR 'anesthetic action'/exp OR 'anesthetic action' OR (('anesthetic'/exp OR anesthetic) AND ('action'/exp OR action)) OR 'anesthetization'/exp OR anesthetization OR 'animal anaesthesia'/exp OR 'animal anaesthesia' OR (('animal'/exp OR animal) AND ('anaesthesia'/exp OR anaesthesia)) OR 'animal anesthesia'/exp OR 'animal anesthesia' OR (('animal'/exp OR animal) AND ('anesthesia'/exp OR anesthesia)) OR 'autoanaesthesia'/exp OR autoanaesthesia OR 'autoanesthesia'/exp OR autoanesthesia OR 'drop mask anaesthesia'/exp OR 'drop mask anaesthesia' OR (drop AND ('mask'/exp OR mask) AND ('anaesthesia'/exp OR anaesthesia)) OR 'drop mask anesthesia'/exp OR 'drop mask anesthesia' OR (drop AND ('mask'/exp OR mask) AND ('anesthesia'/exp OR anesthesia)) OR 'narcosis'/exp OR narcosis OR 'neuroanaesthesia'/exp OR neuroanaesthesia OR 'neuroanesthesia'/exp OR neuroanesthesia OR 'short anaesthesia'/exp OR 'short anaesthesia' OR (short AND ('anaesthesia'/exp OR anaesthesia)) OR 'short anesthesia'/exp OR 'short anesthesia' OR (short AND ('anesthesia'/exp OR anesthesia)) OR 'short duration'/exp OR 'short duration' OR (short AND ('duration'/exp OR duration)) OR 'anaesthesia'/exp OR anaesthesia OR 'short duration anesthesia'/exp OR 'short duration anesthesia' OR (short AND ('duration'/exp OR duration) AND ('anesthesia'/exp OR anesthesia)) OR 'anaesthesia, general'/exp OR 'anaesthesia, general' OR (anaesthesia, AND general) OR 'anaesthesia,general'/exp OR anaesthesia,general OR 'anesthesia, general'/exp OR 'anesthesia, general' OR (anesthesia, AND general) OR 'anesthesia,general'/exp OR anesthesia,general OR 'general anaesthesia'/exp OR 'general anaesthesia' OR (general AND ('anaesthesia'/exp OR anaesthesia)) OR 'anaesthesia,child'/exp OR anaesthesia,child OR 'anaesthesia,paediatric'/exp OR anaesthesia,paediatric OR 'anesthesia,child'/exp OR anesthesia,child OR 'anesthesia,pediatric'/exp OR

© 2022 Reighard C et al. *JAMA Network Open.*

anesthesia,pediatric OR 'child anaesthesia'/exp OR 'child anaesthesia' OR (('child'/exp OR child) AND ('anaesthesia'/exp OR anaesthesia)) OR 'child anesthesia'/exp OR 'child anesthesia' OR (('child'/exp OR child) AND ('anesthesia'/exp OR anesthesia)) OR 'paediatric anaesthesia'/exp OR 'paediatric anaesthesia' OR (('paediatric'/exp OR paediatric) AND ('anaesthesia'/exp OR anaesthesia)) OR 'anaesthesia,intravenous'/exp OR anaesthesia,intravenous OR 'anaesthesia,local,intravenous'/exp OR anaesthesia,local,intravenous OR 'anesthesia, intravenous'/exp OR 'anesthesia, intravenous' OR (anesthesia, AND intravenous) OR 'anesthesia,intravenous'/exp OR anesthesia,intravenous OR 'anesthesia,local,intravenous'/exp OR anesthesia,local,intravenous OR 'intravenous anaesthesia'/exp OR 'intravenous anaesthesia' OR (intravenous AND ('anaesthesia'/exp OR anaesthesia)) OR 'intravenous local anaesthesia'/exp OR 'intravenous local anaesthesia' OR (intravenous AND local AND ('anaesthesia'/exp OR anaesthesia)) OR 'intravenous local anesthesia'/exp OR 'intravenous local anesthesia' OR (intravenous AND local AND ('anesthesia'/exp OR anesthesia)) OR 'local anaesthesia,intravenous'/exp OR 'local anaesthesia,intravenous' OR (local AND ('anaesthesia,intravenous'/exp OR anaesthesia,intravenous)) OR 'local anesthesia,intravenous'/exp OR 'local anesthesia,intravenous' OR (local AND ('anesthesia,intravenous'/exp OR anesthesia,intravenous)) OR 'anaesthesia, closed-circuit'/exp OR 'anaesthesia, closed-circuit' OR (anaesthesia, AND 'closed circuit') OR 'anaesthesia, inhalation'/exp OR 'anaesthesia, inhalation' OR (anaesthesia, AND ('inhalation'/exp OR inhalation)) OR 'anaesthesia,closed circuit'/exp OR 'anaesthesia,closed circuit' OR (anaesthesia,closed AND ('circuit'/exp OR circuit)) OR 'anaesthesia,inhalation'/exp OR anaesthesia,inhalation OR 'anaesthetic system,closed'/exp OR 'anaesthetic system,closed' OR (('anaesthetic'/exp OR anaesthetic) AND system,closed) OR 'anesthesia, closed-circuit'/exp OR 'anesthesia, closed-circuit' OR (anesthesia, AND 'closed circuit') OR 'anesthesia, inhalation'/exp OR 'anesthesia, inhalation' OR (anesthesia, AND ('inhalation'/exp OR inhalation)) OR 'anesthesia,closed circuit'/exp OR 'anesthesia,closed circuit' OR (anesthesia,closed AND ('circuit'/exp OR circuit)) OR 'anesthesia,inhalation'/exp OR anesthesia,inhalation OR 'anesthetic system,closed'/exp OR 'anesthetic system,closed' OR (('anesthetic'/exp OR anesthetic) AND system,closed) OR 'closed anaesthetic circuit'/exp OR 'closed anaesthetic circuit' OR (closed AND ('anaesthetic'/exp OR anaesthetic) AND ('circuit'/exp OR circuit)) OR 'closed anaesthetic system'/exp OR 'closed anaesthetic system' OR (closed AND ('anaesthetic'/exp OR anaesthetic) AND system) OR 'closed anesthetic circuit'/exp OR 'closed anesthetic circuit' OR (closed AND ('anesthetic'/exp OR anesthetic) AND ('circuit'/exp OR circuit)) OR 'closed anesthetic system'/exp OR 'closed anesthetic system' OR (closed AND ('anesthetic'/exp OR anesthetic) AND system) OR 'closed circuit anaesthesia'/exp OR 'closed circuit anaesthesia' OR (closed AND ('circuit'/exp OR circuit) AND ('anaesthesia'/exp OR anaesthesia)) OR 'closed circuit anesthesia'/exp OR 'closed circuit anesthesia' OR (closed AND ('circuit'/exp OR circuit) AND ('anesthesia'/exp OR anesthesia)) OR 'inhalation anaesthesia'/exp OR 'inhalation anaesthesia' OR (('inhalation'/exp OR inhalation) AND ('anaesthesia'/exp OR anaesthesia)) OR 'anaesthetic'/exp OR anaesthetic OR 'anaesthetic agent'/exp OR 'anaesthetic agent' OR (('anaesthetic'/exp OR anaesthetic) AND ('agent'/exp OR agent)) OR 'anaesthetic drug'/exp OR 'anaesthetic drug' OR (('anaesthetic'/exp OR anaesthetic) AND ('drug'/exp OR

© 2022 Reighard C et al. *JAMA Network Open.*

drug)) OR 'anaesthetics'/exp OR anaesthetics OR 'anaesthetics, combined'/exp OR 'anaesthetics, combined' OR (anaesthetics, AND combined) OR 'anaesthetics, dissociative'/exp OR 'anaesthetics, dissociative' OR (anaesthetics, AND dissociative) OR 'anaesthetics, general'/exp OR 'anaesthetics, general' OR (anaesthetics, AND general) OR 'anesthetic'/exp OR anesthetic OR 'anesthetic drug'/exp OR 'anesthetic drug' OR (('anesthetic'/exp OR anesthetic) AND ('drug'/exp OR drug)) OR 'anesthetics'/exp OR anesthetics OR 'anesthetics, combined'/exp OR 'anesthetics, combined' OR (anesthetics, AND combined) OR 'anesthetics, dissociative'/exp OR 'anesthetics, dissociative' OR (anesthetics, AND dissociative) OR 'anesthetics, general'/exp OR 'anesthetics, general' OR (anesthetics, AND general) OR 'general anaesthetic'/exp OR 'general anaesthetic' OR (general AND ('anaesthetic'/exp OR anaesthetic)) OR 'general anaesthetic agent'/exp OR 'general anaesthetic agent' OR (general AND ('anaesthetic'/exp OR anaesthetic) AND ('agent'/exp OR agent)) OR 'general anesthetic'/exp OR 'general anesthetic' OR (general AND ('anesthetic'/exp OR anesthetic)) OR 'general anesthetic agent'/exp OR 'general anesthetic agent' OR (general AND ('anesthetic'/exp OR anesthetic) AND ('agent'/exp OR agent)) OR 'preanaesthetics'/exp OR preanaesthetics OR 'preanesthetics'/exp OR preanesthetics OR 'anaesthetic,volatile'/exp OR anaesthetic,volatile OR 'anaesthetics, inhalation'/exp OR 'anaesthetics, inhalation' OR (anaesthetics, AND ('inhalation'/exp OR inhalation)) OR 'anaesthetics,rapidly evapourating'/exp OR 'anaesthetics,rapidly evapourating' OR (anaesthetics,rapidly AND evapourating) OR 'anesthetic,volatile'/exp OR anesthetic,volatile OR 'anesthetics, inhalation'/exp OR 'anesthetics, inhalation' OR (anesthetics, AND ('inhalation'/exp OR inhalation)) OR 'anesthetics,rapidly evaporating'/exp OR 'anesthetics,rapidly evaporating' OR (anesthetics,rapidly AND evaporating) OR 'gas anaesthetic agent'/exp OR 'gas anaesthetic agent' OR (('gas'/exp OR gas) AND ('anaesthetic'/exp OR anaesthetic) AND ('agent'/exp OR agent)) OR 'gas anesthetic agent'/exp OR 'gas anesthetic agent' OR (('gas'/exp OR gas) AND ('anesthetic'/exp OR anesthetic) AND ('agent'/exp OR agent)) OR 'inhalation anaesthetic'/exp OR 'inhalation anaesthetic' OR (('inhalation'/exp OR inhalation) AND ('anaesthetic'/exp OR anaesthetic)) OR 'inhalation anaesthetic agent'/exp OR 'inhalation anaesthetic agent' OR (('inhalation'/exp OR inhalation) AND ('anaesthetic'/exp OR anaesthetic) AND ('agent'/exp OR agent)) OR 'inhalation anesthetic'/exp OR 'inhalation anesthetic' OR (('inhalation'/exp OR inhalation) AND ('anesthetic'/exp OR anesthetic)) OR 'volatile anaesthetic'/exp OR 'volatile anaesthetic' OR (volatile AND ('anaesthetic'/exp OR anaesthetic)) OR 'volatile anaesthetic agent'/exp OR 'volatile anaesthetic agent' OR (volatile AND ('anaesthetic'/exp OR anaesthetic) AND ('agent'/exp OR agent)) OR 'volatile anesthetic'/exp OR 'volatile anesthetic' OR (volatile AND ('anesthetic'/exp OR anesthetic)) OR 'volatile anesthetic agent'/exp OR 'volatile anesthetic agent' OR (volatile AND ('anesthetic'/exp OR anesthetic) AND ('agent'/exp OR agent)) OR 'anaesthetic,intravenous'/exp OR anaesthetic,intravenous OR 'anaesthetics, intravenous'/exp OR 'anaesthetics, intravenous' OR (anaesthetics, AND intravenous) OR 'anaesthetics,intravenous'/exp OR anaesthetics,intravenous OR 'anesthetic,intravenous'/exp OR anesthetic,intravenous OR 'anesthetics, intravenous'/exp OR 'anesthetics, intravenous' OR (anesthetics, AND intravenous) OR 'anesthetics,intravenous'/exp OR anesthetics,intravenous OR 'intravenous anaesthetic agent'/exp OR 'intravenous anaesthetic agent'

© 2022 Reighard C et al. *JAMA Network Open.*

OR (intravenous AND ('anaesthetic'/exp OR anaesthetic) AND ('agent'/exp OR agent)) OR 'trifluoroethyl ether' OR (trifluoroethyl AND ('ether'/exp OR ether)) OR 'forane'/exp OR forane OR 'forene'/exp OR forene OR 'forthane'/exp OR forthane OR 'isoflurano'/exp OR isoflurano OR 'isorane'/exp OR isorane OR 'sofloran'/exp OR sofloran OR 'sevoflo'/exp OR sevoflo OR 'sevofrane'/exp OR sevofrane OR 'sevorane'/exp OR sevorane OR 'sevotec'/exp OR sevotec OR sojourn OR 'ultane'/exp OR ultane OR 'ultane pen' OR (('ultane'/exp OR ultane) AND ('pen'/exp OR pen)) OR 'sulorane'/exp OR sulorane OR 'suprane'/exp OR suprane OR 'anestane'/exp OR anestane OR 'bromochlorotrifluorethane'/exp OR bromochlorotrifluorethane OR 'fluorothan'/exp OR fluorothan OR 'fluorothane'/exp OR fluorothane OR 'fluothane'/exp OR fluothane OR 'ftorotan'/exp OR ftorotan OR 'halan'/exp OR halan OR 'halothan'/exp OR halothan OR 'halothane metabolite'/exp OR 'halothane metabolite' OR (('halothane'/exp OR halothane) AND ('metabolite'/exp OR metabolite)) OR 'halothane'/exp OR halothane OR 'ineltano'/exp OR ineltano OR 'narcotan'/exp OR narcotan OR 'phthorothane'/exp OR phthorothane OR 'trothane'/exp OR trothane OR 'dinitrogen monoxide'/exp OR 'dinitrogen monoxide' OR (('dinitrogen'/exp OR dinitrogen) AND monoxide) OR 'dinitrogen oxide'/exp OR 'dinitrogen oxide' OR (('dinitrogen'/exp OR dinitrogen) AND ('oxide'/exp OR oxide)) OR 'factitious air'/exp OR 'factitious air' OR (factitious AND ('air'/exp OR air)) OR 'hyponitrous acid anhydride'/exp OR 'hyponitrous acid anhydride' OR (hyponitrous AND ('acid'/exp OR acid) AND ('anhydride'/exp OR anhydride)) OR 'laughing gas'/exp OR 'laughing gas' OR (('laughing'/exp OR laughing) AND ('gas'/exp OR gas)) OR 'nitrogen protoxide'/exp OR 'nitrogen protoxide' OR (('nitrogen'/exp OR nitrogen) AND protoxide) OR 'anepol'/exp OR anepol OR 'cryotol'/exp OR cryotol OR 'diisoprofol'/exp OR diisoprofol OR 'diprivan'/exp OR diprivan OR 'diprofol'/exp OR diprofol OR 'disoprivan'/exp OR disoprivan OR 'disoprofol'/exp OR disoprofol OR 'fresofol'/exp OR fresofol OR 'gobbifol'/exp OR gobbifol OR 'pofol'/exp OR pofol OR 'propocam'/exp OR propocam OR (('propofol'/exp OR propofol) AND ('lipuro'/exp OR lipuro)) OR 'propofol lipuro'/exp OR 'propofol lipuro' OR 'rapinovet'/exp OR rapinovet OR 'recofol'/exp OR recofol OR 'safol'/exp OR safol OR 'anesthal'/exp OR anesthal OR 'farmotal'/exp OR farmotal OR 'hypnostan'/exp OR hypnostan OR 'intraval'/exp OR intraval OR 'leopental'/exp OR leopental OR 'nesdonal'/exp OR nesdonal OR 'penthiobarbital'/exp OR penthiobarbital OR 'penthotal'/exp OR penthotal OR 'pentothal'/exp OR pentothal OR 'pentothal sodico'/exp OR 'pentothal sodico' OR (('pentothal'/exp OR pentothal) AND sodico) OR 'pentothal sodium'/exp OR 'pentothal sodium' OR (('pentothal'/exp OR pentothal) AND ('sodium'/exp OR sodium)) OR 'pharmothal'/exp OR pharmothal OR 'ravonal'/exp OR ravonal OR 'sodipental'/exp OR sodipental OR 'sodium pentothal'/exp OR 'sodium pentothal' OR (('sodium'/exp OR sodium) AND ('pentothal'/exp OR pentothal)) OR 'sodium thiopental'/exp OR 'sodium thiopental' OR (('sodium'/exp OR sodium) AND ('thiopental'/exp OR thiopental)) OR 'thiomebumal'/exp OR thiomebumal OR 'thiomebumal sodium'/exp OR 'thiomebumal sodium' OR (('thiomebumal'/exp OR thiomebumal) AND ('sodium'/exp OR sodium)) OR 'thionembutal'/exp OR thionembutal OR 'thionyl'/exp OR thionyl OR 'thiopental sodium'/exp OR 'thiopental sodium' OR (('thiopental'/exp OR thiopental) AND ('sodium'/exp OR sodium)) OR 'thiopentalbarbital'/exp OR thiopentalbarbital OR 'thiopentemal'/exp OR thiopentemal OR 'thiopenthal'/exp OR thiopenthal OR

© 2022 Reighard C et al. *JAMA Network Open.*

'thiopentobarbital'/exp OR thiopentobarbital OR 'thiopentone'/exp OR thiopentone OR 'thiopentone sodium'/exp OR 'thiopentone sodium' OR (('thiopentone'/exp OR thiopentone) AND ('sodium'/exp OR sodium)) OR 'thiotal'/exp OR thiotal OR 'thiothal'/exp OR thiothal OR 'tio pentemal'/exp OR 'tio pentemal' OR (tio AND pentemal) OR 'tiopental sodico'/exp OR 'tiopental sodico' OR (tiopental AND sodico) OR 'trapanal'/exp OR trapanal OR 'anesject'/exp OR anesject OR 'calipsol'/exp OR calipsol OR 'calypso'/exp OR calypso OR 'imalgene'/exp OR imalgene OR 'kalipsol'/exp OR kalipsol OR 'katamine'/exp OR katamine OR 'keta hameln'/exp OR 'keta hameln' OR 'ketaject'/exp OR ketaject OR 'ketalar'/exp OR ketalar OR 'ketalin'/exp OR ketalin OR 'ketamax'/exp OR ketamax OR 'ketamine hcl'/exp OR 'ketamine hcl' OR (('ketamine'/exp OR ketamine) AND hcl) OR 'ketamine hydrochloride'/exp OR 'ketamine hydrochloride' OR (('ketamine'/exp OR ketamine) AND ('hydrochloride'/exp OR hydrochloride)) OR 'ketaminol vet'/exp OR 'ketaminol vet' OR (('ketaminol'/exp OR ketaminol) AND vet) OR 'ketanest'/exp OR ketanest OR 'ketased'/exp OR ketased OR 'ketaset'/exp OR ketaset OR 'ketaved'/exp OR ketaved OR 'ketavet'/exp OR ketavet OR 'ketmin'/exp OR ketmin OR 'ketoject'/exp OR ketoject OR 'ketolar'/exp OR ketolar OR 'narkamon'/exp OR narkamon OR 'narketan'/exp OR narketan OR 'soon soon'/exp OR 'soon soon' OR 'tekam'/exp OR tekam OR 'velonarcon'/exp OR velonarcon OR 'vetalar'/exp OR vetalar OR 'buccolam'/exp OR buccolam OR 'dalam'/exp OR dalam OR 'doricum'/exp OR doricum OR 'dormonid'/exp OR dormonid OR 'fortanest'/exp OR fortanest OR 'fulsed'/exp OR fulsed OR 'hypnoval'/exp OR hypnoval OR 'hypnovel'/exp OR hypnovel OR 'hypnoyvel'/exp OR hypnoyvel OR 'ipnovel'/exp OR ipnovel OR 'midacum'/exp OR midacum OR 'midazo'/exp OR midazo OR 'midazol'/exp OR midazol OR 'midazolam hydrochloride'/exp OR 'midazolam hydrochloride' OR (('midazolam'/exp OR midazolam) AND ('hydrochloride'/exp OR hydrochloride)) OR 'midazolam hydrochloride preservative free'/exp OR 'midazolam hydrochloride preservative free' OR (('midazolam'/exp OR midazolam) AND ('hydrochloride'/exp OR hydrochloride) AND ('preservative'/exp OR preservative) AND free) OR 'midolam'/exp OR midolam OR 'miloz'/exp OR miloz OR 'versed'/exp OR versed OR 'benzodiazepine'/exp OR benzodiazepine OR 'benzodiazepin derivative'/exp OR 'benzodiazepin derivative' OR (benzodiazepin AND derivative) OR 'benzodiazepines'/exp OR benzodiazepines OR 'benzodiazepinones'/exp OR benzodiazepinones OR 'benzodiazepine agonist'/exp OR 'benzodiazepine agonist' OR (('benzodiazepine'/exp OR benzodiazepine) AND ('agonist'/exp OR agonist)) OR 'benzodiazepine receptor agonist'/exp OR 'benzodiazepine receptor agonist' OR (('benzodiazepine'/exp OR benzodiazepine) AND ('receptor'/exp OR receptor) AND ('agonist'/exp OR agonist)) OR 'benzodiazepine receptor stimulant'/exp OR 'benzodiazepine receptor stimulant' OR (('benzodiazepine'/exp OR benzodiazepine) AND ('receptor'/exp OR receptor) AND ('stimulant'/exp OR stimulant)) OR 'benzodiazepine receptor stimulator'/exp OR 'benzodiazepine receptor stimulator' OR (('benzodiazepine'/exp OR benzodiazepine) AND ('receptor'/exp OR receptor) AND ('stimulator'/exp OR stimulator)) OR 'benzodiazepine stimulant'/exp OR 'benzodiazepine stimulant' OR (('benzodiazepine'/exp OR benzodiazepine) AND ('stimulant'/exp OR stimulant)) OR 'benzodiazepine stimulating agent'/exp OR 'benzodiazepine stimulating agent' OR (('benzodiazepine'/exp OR benzodiazepine) AND stimulating AND ('agent'/exp OR agent)) OR 'benzodiazepine

© 2022 Reighard C et al. *JAMA Network Open.*

stimulator'/exp OR 'benzodiazepine stimulator' OR (('benzodiazepine'/exp OR benzodiazepine) AND ('stimulator'/exp OR stimulator)) OR 'arkamin'/exp OR arkamin OR 'atensina'/exp OR atensina OR 'caprysin'/exp OR caprysin OR 'catapres'/exp OR catapres OR 'catapres tts'/exp OR 'catapres tts' OR (('catapres'/exp OR catapres) AND tts) OR 'catapresan'/exp OR catapresan OR 'catapresan depot'/exp OR 'catapresan depot' OR (('catapresan'/exp OR catapresan) AND ('depot'/exp OR depot)) OR 'catapresan tts'/exp OR 'catapresan tts' OR (('catapresan'/exp OR catapresan) AND tts) OR 'catapressan'/exp OR catapressan OR 'catapressan perlonguettes'/exp OR 'catapressan perlonguettes' OR (('catapressan'/exp OR catapressan) AND perlonguettes) OR 'catapressant'/exp OR catapressant OR 'catasan'/exp OR catasan OR 'chlofazolin'/exp OR chlofazolin OR 'chlophazolin'/exp OR chlophazolin OR 'chlophelin'/exp OR chlophelin OR 'clofelin'/exp OR clofelin OR 'clofeline'/exp OR clofeline OR 'clomidine'/exp OR clomidine OR 'clonicel'/exp OR clonicel OR 'clonidine'/exp OR clonidine OR 'clonidine chlorhydrate'/exp OR 'clonidine chlorhydrate' OR (('clonidine'/exp OR clonidine) AND ('chlorhydrate'/exp OR chlorhydrate)) OR 'clonidine hydrochloride'/exp OR 'clonidine hydrochloride' OR (('clonidine'/exp OR clonidine) AND ('hydrochloride'/exp OR hydrochloride)) OR 'clonipresan'/exp OR clonipresan OR 'clonistada'/exp OR clonistada OR 'clonistada retard'/exp OR 'clonistada retard' OR (('clonistada'/exp OR clonistada) AND ('retard'/exp OR retard)) OR 'clonnirit'/exp OR clonnirit OR 'clophelin'/exp OR clophelin OR 'clopheline'/exp OR clopheline OR 'daipres'/exp OR daipres OR 'dcai'/exp OR dcai OR 'dichlorophenylaminoimidazoline'/exp OR dichlorophenylaminoimidazoline OR 'dichlorophenylaminoimidazoline hydrochloride'/exp OR 'dichlorophenylaminoimidazoline hydrochloride' OR (('dichlorophenylaminoimidazoline'/exp OR dichlorophenylaminoimidazoline) AND ('hydrochloride'/exp OR hydrochloride)) OR 'dixarit'/exp OR dixarit OR 'duraclon'/exp OR duraclon OR 'haemiton'/exp OR haemiton OR 'hemiton'/exp OR hemiton OR 'huma clonidine'/exp OR 'huma clonidine' OR 'hypodine'/exp OR hypodine OR 'isoglaucon'/exp OR isoglaucon OR 'jenloga'/exp OR jenloga OR 'kapvay'/exp OR kapvay OR 'melzin'/exp OR melzin OR 'normopresan'/exp OR normopresan OR 'normopresin'/exp OR normopresin OR 'paracefan'/exp OR paracefan OR 'sulmidine'/exp OR sulmidine OR 'taitecin'/exp OR taitecin OR 'tenso timelets'/exp OR 'tenso timelets' OR (tenso AND timelets) OR 'adrenergic alpha agonists'/exp OR 'adrenergic alpha agonists' OR (adrenergic AND alpha AND ('agonists'/exp OR agonists)) OR 'adrenergic alpha-agonists'/exp OR 'adrenergic alpha-agonists' OR (adrenergic AND 'alpha agonists') OR 'alpha adrenergic agent'/exp OR 'alpha adrenergic agent' OR (alpha AND adrenergic AND ('agent'/exp OR agent)) OR 'alpha adrenergic agonist'/exp OR 'alpha adrenergic agonist' OR (alpha AND adrenergic AND ('agonist'/exp OR agonist)) OR 'alpha adrenergic receptor agent'/exp OR 'alpha adrenergic receptor agent' OR (alpha AND adrenergic AND ('receptor'/exp OR receptor) AND ('agent'/exp OR agent)) OR 'alpha adrenergic receptor agonist'/exp OR 'alpha adrenergic receptor agonist' OR (alpha AND adrenergic AND ('receptor'/exp OR receptor) AND ('agonist'/exp OR agonist)) OR 'alpha adrenergic receptor stimulant'/exp OR 'alpha adrenergic receptor stimulant' OR (alpha AND adrenergic AND ('receptor'/exp OR receptor) AND ('stimulant'/exp OR stimulant)) OR 'alpha adrenergic receptor stimulator'/exp OR 'alpha adrenergic receptor stimulator' OR (alpha AND adrenergic AND ('receptor'/exp OR receptor) AND ('stimulator'/exp OR

© 2022 Reighard C et al. *JAMA Network Open.*

stimulator)) OR 'alpha adrenergic stimulant'/exp OR 'alpha adrenergic stimulant' OR (alpha AND adrenergic AND ('stimulant'/exp OR stimulant)) OR 'alpha adrenergic stimulating agent'/exp OR 'alpha adrenergic stimulating agent' OR (alpha AND adrenergic AND stimulating AND ('agent'/exp OR agent)) OR 'alpha adrenergic stimulator'/exp OR 'alpha adrenergic stimulator' OR (alpha AND adrenergic AND ('stimulator'/exp OR stimulator)) OR 'alpha adrenoceptor agonist'/exp OR 'alpha adrenoceptor agonist' OR (alpha AND ('adrenoceptor'/exp OR adrenoceptor) AND ('agonist'/exp OR agonist)) OR 'alpha adrenoceptor stimulant'/exp OR 'alpha adrenoceptor stimulant' OR (alpha AND ('adrenoceptor'/exp OR adrenoceptor) AND ('stimulant'/exp OR stimulant)) OR 'alpha adrenoceptor stimulating agent'/exp OR 'alpha adrenoceptor stimulating agent' OR (alpha AND ('adrenoceptor'/exp OR adrenoceptor) AND stimulating AND ('agent'/exp OR agent)) OR 'alpha adrenoceptor stimulator'/exp OR 'alpha adrenoceptor stimulator' OR (alpha AND ('adrenoceptor'/exp OR adrenoceptor) AND ('stimulator'/exp OR stimulator)) OR 'alpha agonist'/exp OR 'alpha agonist' OR (alpha AND ('agonist'/exp OR agonist)) OR 'alpha sympathicomimetic'/exp OR 'alpha sympathicomimetic' OR (alpha AND ('sympathicomimetic'/exp OR sympathicomimetic)) OR 'alpha sympathicomimetic agent'/exp OR 'alpha sympathicomimetic agent' OR (alpha AND ('sympathicomimetic'/exp OR sympathicomimetic) AND ('agent'/exp OR agent)) OR 'noradrenalin agonist'/exp OR 'noradrenalin agonist' OR (('noradrenalin'/exp OR noradrenalin) AND ('agonist'/exp OR agonist)) OR 'noradrenergic agonist'/exp OR 'noradrenergic agonist' OR (noradrenergic AND ('agonist'/exp OR agonist)) OR 'noradrenergic receptor stimulating agent'/exp OR 'noradrenergic receptor stimulating agent' OR (noradrenergic AND ('receptor'/exp OR receptor) AND stimulating AND ('agent'/exp OR agent)) OR 'dexamedetomidine'/exp OR dexamedetomidine OR 'dexdomitor'/exp OR dexdomitor OR 'dexdor'/exp OR dexdor OR 'dexmedetomidine hydrochloride'/exp OR 'dexmedetomidine hydrochloride' OR (('dexmedetomidine'/exp OR dexmedetomidine) AND ('hydrochloride'/exp OR hydrochloride)) OR precede OR 'primadex'/exp OR primadex OR 'sileo'/exp OR sileo OR 'gaba agonist'/exp OR 'gaba agonist' OR (('gaba'/exp OR gaba) AND ('agonist'/exp OR agonist)) OR 'gaba agonists'/exp OR 'gaba agonists' OR (('gaba'/exp OR gaba) AND ('agonists'/exp OR agonists)) OR 'gaba receptor agonist'/exp OR 'gaba receptor agonist' OR (('gaba'/exp OR gaba) AND ('receptor'/exp OR receptor) AND ('agonist'/exp OR agonist)) OR 'gaba receptor stimulant'/exp OR 'gaba receptor stimulant' OR (('gaba'/exp OR gaba) AND ('receptor'/exp OR receptor) AND ('stimulant'/exp OR stimulant)) OR 'gaba receptor stimulating agent'/exp OR 'gaba receptor stimulating agent' OR (('gaba'/exp OR gaba) AND ('receptor'/exp OR receptor) AND stimulating AND ('agent'/exp OR agent)) OR 'gaba receptor stimulator'/exp OR 'gaba receptor stimulator' OR (('gaba'/exp OR gaba) AND ('receptor'/exp OR receptor) AND ('stimulator'/exp OR stimulator)) OR 'gabaergic receptor agonist'/exp OR 'gabaergic receptor agonist' OR (gabaergic AND ('receptor'/exp OR receptor) AND ('agonist'/exp OR agonist)) OR 'gabaergic receptor stimulant'/exp OR 'gabaergic receptor stimulant' OR (gabaergic AND ('receptor'/exp OR receptor) AND ('stimulant'/exp OR stimulant)) OR 'gabaergic receptor stimulating agent'/exp OR 'gabaergic receptor stimulating agent' OR (gabaergic AND ('receptor'/exp OR receptor) AND stimulating AND ('agent'/exp OR agent)) OR 'gabaergic receptor stimulator'/exp OR 'gabaergic receptor stimulator' OR (gabaergic AND ('receptor'/exp

OR receptor) AND ('stimulator'/exp OR stimulator)) OR 'gabamimetic'/exp OR gabamimetic OR 'gabamimetic agent'/exp OR 'gabamimetic agent' OR (('gabamimetic'/exp OR gabamimetic) AND ('agent'/exp OR agent)) OR 'gamma aminobutyric acid agonist'/exp OR 'gamma aminobutyric acid agonist' OR (gamma AND aminobutyric AND ('acid'/exp OR acid) AND ('agonist'/exp OR agonist)) OR 'gamma aminobutyric acid receptor agonist'/exp OR 'gamma aminobutyric acid receptor agonist' OR (gamma AND aminobutyric AND ('acid'/exp OR acid) AND ('receptor'/exp OR receptor) AND ('agonist'/exp OR agonist)) OR 'gamma aminobutyric acid receptor stimulant'/exp OR 'gamma aminobutyric acid receptor stimulant' OR (gamma AND aminobutyric AND ('acid'/exp OR acid) AND ('receptor'/exp OR receptor) AND ('stimulant'/exp OR stimulant)) OR 'gamma aminobutyric acid receptor stimulating agent'/exp OR 'gamma aminobutyric acid receptor stimulating agent' OR (gamma AND aminobutyric AND ('acid'/exp OR acid) AND ('receptor'/exp OR receptor) AND stimulating AND ('agent'/exp OR agent)) OR 'gamma aminobutyric acid receptor stimulator'/exp OR 'gamma aminobutyric acid receptor stimulator' OR (gamma AND aminobutyric AND ('acid'/exp OR acid) AND ('receptor'/exp OR receptor) AND ('stimulator'/exp OR stimulator)) OR 'n-methyl d-aspartate antagonist'/exp OR 'n-methyl d-aspartate antagonist' OR ('n methyl' AND ('d aspartate'/exp OR 'd aspartate') AND antagonist) OR 'n-methyl d-aspartate blocker'/exp OR 'n-methyl d-aspartate blocker' OR ('n methyl' AND ('d aspartate'/exp OR 'd aspartate') AND blocker) OR 'n-methyl d-aspartate blocking agent'/exp OR 'n-methyl d-aspartate blocking agent' OR ('n methyl' AND ('d aspartate'/exp OR 'd aspartate') AND blocking AND ('agent'/exp OR agent)) OR 'n-methyl d-aspartate receptor antagonist'/exp OR 'n-methyl d-aspartate receptor antagonist' OR ('n methyl' AND ('d aspartate'/exp OR 'd aspartate') AND ('receptor'/exp OR receptor) AND antagonist) OR 'n-methyl d-aspartate receptor blocker'/exp OR 'n-methyl d-aspartate receptor blocker' OR ('n methyl' AND ('d aspartate'/exp OR 'd aspartate') AND ('receptor'/exp OR receptor) AND blocker) OR 'n-methyl d-aspartate receptor blocking agent'/exp OR 'n-methyl d-aspartate receptor blocking agent' OR ('n methyl' AND ('d aspartate'/exp OR 'd aspartate') AND ('receptor'/exp OR receptor) AND blocking AND ('agent'/exp OR agent)) OR 'n-methyl d-aspartic acid antagonist'/exp OR 'n-methyl d-aspartic acid antagonist' OR ('n methyl' AND 'd aspartic' AND ('acid'/exp OR acid) AND antagonist) OR 'n-methyl d-aspartic acid blocker'/exp OR 'n-methyl d-aspartic acid blocker' OR ('n methyl' AND 'd aspartic' AND ('acid'/exp OR acid) AND blocker) OR 'n-methyl d-aspartic acid blocking agent'/exp OR 'n-methyl d-aspartic acid blocking agent' OR ('n methyl' AND 'd aspartic' AND ('acid'/exp OR acid) AND blocking AND ('agent'/exp OR agent)) OR 'n-methyl d-aspartic acid receptor antagonist'/exp OR 'n-methyl d-aspartic acid receptor antagonist' OR ('n methyl' AND 'd aspartic' AND ('acid'/exp OR acid) AND ('receptor'/exp OR receptor) AND antagonist) OR 'n-methyl d-aspartic acid receptor blocker'/exp OR 'n-methyl d-aspartic acid receptor blocker' OR ('n methyl' AND 'd aspartic' AND ('acid'/exp OR acid) AND ('receptor'/exp OR receptor) AND blocker) OR 'n-methyl d-aspartic acid receptor blocking agent'/exp OR 'n-methyl d-aspartic acid receptor blocking agent' OR ('n methyl' AND 'd aspartic' AND ('acid'/exp OR acid) AND ('receptor'/exp OR receptor) AND blocking AND ('agent'/exp OR agent)) OR 'n-methyl dextro aspartate antagonist'/exp OR 'n-methyl dextro aspartate antagonist' OR ('n methyl' AND dextro AND ('aspartate'/exp OR aspartate) AND antagonist) OR 'n-methyl dextro

aspartate blocker'/exp OR 'n-methyl dextro aspartate blocker' OR ('n methyl' AND dextro AND ('aspartate'/exp OR aspartate) AND blocker) OR 'n-methyl dextro aspartate blocking agent'/exp OR 'n-methyl dextro aspartate blocking agent' OR ('n methyl' AND dextro AND ('aspartate'/exp OR aspartate) AND blocking AND ('agent'/exp OR agent)) OR 'n-methyl dextro aspartate receptor antagonist'/exp OR 'n-methyl dextro aspartate receptor antagonist' OR ('n methyl' AND dextro AND ('aspartate'/exp OR aspartate) AND ('receptor'/exp OR receptor) AND antagonist) OR 'n-methyl dextro aspartate receptor blocker'/exp OR 'n-methyl dextro aspartate receptor blocker' OR ('n methyl' AND dextro AND ('aspartate'/exp OR aspartate) AND ('receptor'/exp OR receptor) AND blocker) OR 'n-methyl dextro aspartate receptor blocking agent'/exp OR 'n-methyl dextro aspartate receptor blocking agent' OR ('n methyl' AND dextro AND ('aspartate'/exp OR aspartate) AND ('receptor'/exp OR receptor) AND blocking AND ('agent'/exp OR agent)) OR 'n-methyl dextro aspartic acid antagonist'/exp OR 'n-methyl dextro aspartic acid antagonist' OR ('n methyl' AND dextro AND aspartic AND ('acid'/exp OR acid) AND antagonist) OR 'n-methyl dextro aspartic acid blocker'/exp OR 'n-methyl dextro aspartic acid blocker' OR ('n methyl' AND dextro AND aspartic AND ('acid'/exp OR acid) AND blocker) OR 'n-methyl dextro aspartic acid blocking agent'/exp OR 'n-methyl dextro aspartic acid blocking agent' OR ('n methyl' AND dextro AND aspartic AND ('acid'/exp OR acid) AND blocking AND ('agent'/exp OR agent)) OR 'n-methyl dextro aspartic acid receptor antagonist'/exp OR 'n-methyl dextro aspartic acid receptor antagonist' OR ('n methyl' AND dextro AND aspartic AND ('acid'/exp OR acid) AND ('receptor'/exp OR receptor) AND antagonist) OR 'n-methyl dextro aspartic acid receptor blocker'/exp OR 'n-methyl dextro aspartic acid receptor blocker' OR ('n methyl' AND dextro AND aspartic AND ('acid'/exp OR acid) AND ('receptor'/exp OR receptor) AND blocker) OR 'nmda antagonist'/exp OR 'nmda antagonist' OR (('nmda'/exp OR nmda) AND antagonist) OR 'nmda blocker'/exp OR 'nmda blocker' OR (('nmda'/exp OR nmda) AND blocker) OR 'nmda blocking agent'/exp OR 'nmda blocking agent' OR (('nmda'/exp OR nmda) AND blocking AND ('agent'/exp OR agent)) OR 'nmda receptor antagonist'/exp OR 'nmda receptor antagonist' OR (('nmda'/exp OR nmda) AND ('receptor'/exp OR receptor) AND antagonist) OR 'nmda receptor antagonists'/exp OR 'nmda receptor antagonists' OR (('nmda'/exp OR nmda) AND ('receptor'/exp OR receptor) AND antagonists) OR 'nmda receptor blocker'/exp OR 'nmda receptor blocker' OR (('nmda'/exp OR nmda) AND ('receptor'/exp OR receptor) AND blocker) OR 'nmda receptor blocking agent'/exp OR 'nmda receptor blocking agent' OR (('nmda'/exp OR nmda) AND ('receptor'/exp OR receptor) AND blocking AND ('agent'/exp OR agent))

39. #15 or #16 or #17 or #18 or #19 or #20 or #21 or #22 or #23 or #24 or #25 or #26 or #27 or #28 or #29 or #30 or #31 or #32 or #33 or #34 or #35 or #36 or #37 or #38

40. ‘cognitive accessibility’ or ‘cognitive balance’ or ‘cognitive dissonance’ or ‘cognitive function’ or ‘cognitive structure’ or ‘cognitive symptoms’ or ‘cognitive task’ or ‘cognitive thinking’ or ‘neurobehavioral manifestations’ or ‘neurobehavioural manifestations’ or ‘volition’ or ‘cognition disorder’ or ‘cognition disorders’ or ‘cognitive defects’ or ‘cognitive deficit’ or ‘cognitive disability’ or ‘cognitive disorder’ or ‘cognitive disorders’ or ‘cognitive dysfunction’ or ‘cognitive impairment’ or ‘delirium, dementia, amnestic, cognitive

© 2022 Reighard C et al. *JAMA Network Open.*

disorders' or 'overinclusion' or 'response interference' or 'behavior, child' or 'behaviour, child' or 'child behavior' or 'infant behavior' or 'infant behavior' or 'disorder, learning' or 'impaired learning' or 'learning deficit' or 'learning difficulty' or 'learning disabilities' or 'learning disability' or 'learning disorders' or 'learning disturbance' or 'learning impairment' or 'learning problem' or 'abnormal development' or 'child development disorder' or 'development disorder' or 'developmental disabilities'

41. 'developmental disorder'/exp or 'Learning disorder'/exp or 'Child behavior'/exp or 'Postoperative cognitive dysfunction'/exp or 'Cognitive defect'/exp or 'Cognition assessment'/exp or 'Cognition'/exp

42. #40 or #41

43. #14 and #39 and #42

44. not (('animal'/exp or 'nonhuman'/exp) not 'human'/exp)

© 2022 Reighard C et al. *JAMA Network Open.*

CINAHL

S41	S6 AND S32 AND S40
S40	S33 OR S34 OR S35 OR S36 OR S37 OR S38 OR S39
S39	Cognition Disorder OR Cognition Disorders OR Cognitive Disorders OR Cognitive Disorder OR Deficiency, Mental OR Disability, Intellectual OR Mental Deficiency OR Mental Retardation OR Mental Retardation, Psychosocial OR Retardation, Mental OR Intellectual Disabilities OR Learning Disabilities OR Learning Disorders, Chronic OR Cognitive Symptoms OR Signs and Symptoms, Neurobehavioral OR Cognitive Symptom OR Neurobehavioral Manifestation OR Neurobehavioral Sign and Symptom OR Neurobehavioral Sign Symptom OR Neurobehavioral Signs and Symptoms OR Neurobehavioral Signs Symptoms OR Neurobehavioural Manifestation OR Neurobehavioural Signs and Symptoms OR Signs and Symptoms, Neurobehavioural OR Disabilities, Developmental OR Developmental Disability OR Child Development Disorder OR Children Development Disorders OR Infant Development Disorders
S38	(MH "Developmental Disabilities")
S37	(MH "Neurobehavioral Manifestations+")
S36	(MH "Intellectual Disability+")
S35	(MH "Child Development Disorders+")
S34	(MH "Cognition Disorders+")
S33	(MH "Cognition+")
S32	S7 OR S8 OR S9 OR S10 OR S11 OR S12 OR S13 OR S14 OR S15 OR S16 OR S17 OR S18 OR S19 OR S20 OR S21 OR S22 OR S23 OR S24 OR S25 OR S26 OR S27 OR S28 OR S29 OR S30 OR S31
S31	Anesthesias, General OR General anesthesias OR general anesthesia OR Anesthesias, Inhalation OR Anesthesias, Intravenous OR Anesthesia, Obstetrical OR Anesthesias, Obstetrical OR Hypnosis, Anaesthetic OR Hypnosis, Anaesthetics OR Anaesthesia, General OR Anaesthesias, General OR General anaesthesias OR general anaesthesia OR Anaesthesia, Inhalation OR Anaesthesias, Inhalation OR Anaesthesia, Intravenous OR Anaesthesias, Intravenous OR Anaesthesia, Obstetrical OR Anaesthesias, Obstetrical OR Hypnosis, Anaesthetic OR Hypnosis, Anaesthetics OR General Anesthetics OR General Anesthetics OR Anesthetic, General OR Inhalation Anesthetics OR Anesthetic, Inhalation OR General Anaesthetics OR Anaesthetic, General OR General Anaesthetics OR Anaesthetic, General OR Anaesthetics,

© 2022 Reighard C et al. *JAMA Network Open.*

	Inhalation OR Inhalation Anaesthetics OR Anaesthetic, Inhalation OR Barbiturates OR Fluothane OR Desflurane OR Anesthetic, Intravenous OR Intravenous Anesthetics OR Intravenous Anesthetic OR Anaesthetics, Intravenous OR Anaesthetic, Intravenous OR Intravenous Anaesthetics OR Intravenous Anaesthetic OR Anesthetic OR Anesthetics OR Anaesthetic OR Anaesthetics OR Sedatives, Barbiturate OR Sedatives, Barbiturates OR Sedatives, Nonbarbiturate OR Sedatives, Nonbarbiturates OR Sodium Oxybate OR Alprazolam OR Chlordiazepoxide OR Clorazepate Dipotassium OR Flunitrazepam OR Flurazepam OR Lorazepam OR Oxazepam OR Temazepam OR Quazepam OR Catapres OR Catapresan OR Catapressan OR Chlophazolin OR Clofelin OR Clofenil OR Clopheline OR Clonidine Dihydrochloride OR Clonidine Monohydrobromide OR Clonidine Monohydrochloride OR Dixarit OR Gemiton OR Hemiton OR Isoglaucon OR Klofelin OR Klofenil OR Clonidine Hydrochloride OR Dexmedetomidine OR Adrenergic Alpha-Agonists OR Adrenergic Alpha-Agonist OR Alpha-Adrenergic Receptor Agonists OR Adrenergic Alpha-Agonist OR Alpha Agonists OR Alpha Agonists, Adrenergic OR Alpha-Adrenergic Receptor Agonist OR Receptor Agonists, Adrenergic Alpha OR Receptor Agonists, Alpha-Adrenergic OR Gamma-Aminobutyric Acid Agonists OR GABA Agonist OR Agonists, GABA OR Agonist GABA OR Gamma Aminobutyric Acid Agonist OR Amino Acids, Excitatory, Antagonists OR Glutamate Antagonists OR Amino Acid Antagonists, Excitatory OR Amino Acid Antagonist Excitatory OR Amino Acids Excitatory Antagonist OR Glutamate Antagonist OR Early Exposure
S30	(MH "Adrenergic Alpha-Agonists+")
S29	(MH "Excitatory Amino Acid Antagonists+")
S28	(MH "GABA Modulators+")
S27	(MH "GABA Agonists+")
S26	(MH "Clonidine")
S25	(MH "Anesthetics+")
S24	(MH "Ketamine")
S23	(MH "Antianxiety Agents, Benzodiazepine+")
S22	(MH "Midazolam")
S21	(MH "Sedatives, Barbiturate+")
S20	(MH "Barbiturates+")

© 2022 Reighard C et al. *JAMA Network Open.*

S19	(MH "Thiopental")
S18	(MH "Propofol")
S17	(MH "Nitrous Oxide")
S16	(MH "Halothane")
S15	(MH "Isoflurane")
S14	(MH "Sevoflurane")
S13	(MH "Isoflurane")
S12	(MH "Anesthetics, Intravenous+")
S11	(MH "Anesthetics, Inhalation+")
S10	(MH "Anesthesia, Intravenous")
S9	(MH "Anesthesia, Inhalation")
S8	(MH "Anesthetics, General+")
S7	(MH "Anesthesia, General+")
S6	S1 OR S2 OR S3 OR S4 OR S5
S5	Children OR Childhood OR Child, Abandoned OR Child, Adopted OR Child, Disabled OR Child, Foster OR Child, Gifted OR Child, Hospitalized OR Child, Institutionalized OR Child, Medically Fragile OR Child, Preschool OR Infants OR Infancy OR Infant, Drug-Exposed OR Infant, High Risk OR Infant, Hospitalized OR Newborn Infant OR Newborn Infants OR Neonate OR Neonates OR Baby Newborn OR Adolescent OR Adolescent, Hospitalized OR Adolescents OR Youth OR Youths OR Teenager OR Teenagers OR Teens OR Teen Ager OR Teen Agers OR Toddler OR Toddlers
S4	(MH "Child, Preschool")
S3	(MH "Adolescence+")
S2	(MH "Child+")
S1	(MH "Infant, Newborn+")

Web of Science

#4	#3 AND #2 AND #1 NOT SU=(Veterinary Sciences) NOT TS=(mouse or mice or rat)<br>Refined by: LANGUAGES: (ENGLISH)
#3	(Cognition OR Cognition disorder OR Cognition disorders OR Cognition impairment OR Cognition Impairments OR Cognition deficit OR Cognition deficits OR Cognitive defect OR Cognitive defects OR Cognitive disorder OR Cognitive disorders OR Cognitive impairment OR Cognitive impairments OR Cognitive deficit OR Cognitive deficits OR Cognitive Dysfunction OR Cognitive Dysfunctions OR Development disability OR Development disabilities OR Development disabilities OR Developmental disabilities OR Child development deviation OR Child development disorder OR Child development disorders OR Developmental delay disorder OR Developmental delay disorders OR Neurodevelopment OR Neurodevelopment outcome OR Learning impairment OR Learning disability OR Learning disabilities OR Behavioral impairment OR Behavioral change OR Behavioral Changes OR Neurodegeneration OR Intellectual deficiency OR Intellectual deficiencies OR Neurobehavior OR Neurobehavioral manifestation OR Neurobehavioral manifestations)
#2	(General Anesthesia OR General Anaesthesia OR General Anesthetic OR General Anesthetics OR General Anaesthetic OR General Anaesthetics OR Intravenous Anesthetic OR Intravenous Anaesthetic OR Intravenous Anesthetics OR Intravenous Anaesthetics OR Inhalation Anesthesia OR Inhalation Anaesthesia OR Isoflurane OR Desflurane OR Sevoflurane OR Nitrous Oxide OR Halothane OR Propofol OR Thiopental OR Ketamine OR Clonidine OR Dexmedetomidine OR Midazolam OR Benzodiazepine OR GABA-Agonist OR NMDA-Antagonist OR Early Exposure OR Methyl-D-Aspartate OR Gamma Aminobutyric Acid Agonist OR Gamma Aminoburytic Acid Agonists OR Sedative OR Sedatives)
#1	(Child OR Children OR Childhood OR Infant OR Infants OR Infancy OR Adolescent OR Adolescents OR Adolescence OR Preschool Child OR Preschool Children OR School Child OR School Children OR Baby OR Babies OR Newborn OR Newborns OR Newborn Child OR Newborn Children OR Neonate OR Neonates OR Toddler OR Toddlers OR Teenager OR Teenagers OR Teenage)

© 2022 Reighard C et al. *JAMA Network Open.*

Cochrane/CENTRAL

ID Search

#1 MeSH descriptor: [Child] explode all trees

#2 MeSH descriptor: [Infant] explode all trees

#3 MeSH descriptor: [Adolescent] explode all trees

#4 Child or Children or child, preschool or children, preschool or preschool child or preschool children or Infant or Infants or infant, newborn or infants, newborn or neonate or neonates or newborn infant or newborn infants or newborn or newborns or adolescence or adolescent, female or adolescent, male or adolescent or adolescents, female or adolescents, male or adolescents or female adolescent or female adolescents or male adolescent or male adolescents or teen or teenager or teenagers or teens or youth or youths or Baby or Babies or Toddler or Toddlers or Infancy or Childhood:ti,ab,kw (Word variations have been searched)

#5 #1 or #2 or #3 or #4

#6 MeSH descriptor: [Anesthesia, General] explode all trees

#7 MeSH descriptor: [Anesthetics] explode all trees

#8 MeSH descriptor: [Anesthetics, General] explode all trees

#9 MeSH descriptor: [Anesthetics, Inhalation] explode all trees

#10 MeSH descriptor: [Nitrous Oxide] explode all trees

#11 MeSH descriptor: [Anesthetics, Intravenous] explode all trees

#12 MeSH descriptor: [Propofol] explode all trees

#13 MeSH descriptor: [Hypnotics and Sedatives] explode all trees

#14 MeSH descriptor: [Thiopental] explode all trees

#15 MeSH descriptor: [GABA Modulators] explode all trees

#16 MeSH descriptor: [GABA Agonists] explode all trees

#17 MeSH descriptor: [Excitatory Amino Acid Antagonists] explode all trees

#18 MeSH descriptor: [Adrenergic alpha-2 Receptor Agonists] explode all trees

#19 MeSH descriptor: [Benzodiazepines] explode all trees

#20 Anesthesia, General or Anesthesias, General or General Anesthesia or General Anesthesias or Anesthesia, Inhalation or Inhalation Anesthesia or Insufflation Anesthesia or Anesthesia, Insufflation or Anesthesia, Intravenous or Anesthesias, Intravenous or Intravenous Anesthesias or Intravenous Anesthesias or Anesthetics, General or General Anesthetic or Anesthetics, Inhalation or Inhalation Anesthetics or Anesthetic Gases or Gases, Anesthetic or Anesthetics, Intravenous or Clonidine or Clonidine Dihydrochloride

or Dihydrochloride, Clonidine or Clonidine Hydrochloride or Hydrochloride, Clonidine or Clonidine Monohydrochloride Monohydrochloride, Clonidine or Clonidine Monohydrobromide Monohydrobromide, Clonidine or Boehringer Ingelheim Brand of Clonidine Hydrochloride or Adrenergic alpha 2 Receptor Agonists or Adrenergic alpha2 Agonists or Adrenergic alpha2 Agonists or alpha2 Agonists, Adrenergic or Adrenergic alpha-2 Agonists or Adrenergic alpha 2 Agonists or Agonists, Adrenergic alpha-2 or alpha-2 Agonists, Adrenergic or Adrenergic alpha-2 Receptor Agonist or Adrenergic alpha 2 Receptor Agonist or Dexmedetomidine or Hospira Brand of Dexmedetomidine Hydrochloride or Dexmedetomidine Hydrochloride or Hydrochloride, Dexmedetomidine or Midazolam or Midazolam Maleate or Maleate, Midazolam or Midazolam Hydrochloride or Hydrochloride, Midazolam or Benzodiazepine or Benzodiazepine Compounds or Benzodiazepines or Intravenous Anesthetics or Anesthetic Drug or Anesthetic Drugs or Drugs, Anesthetic or Drug, Anesthetic or Anesthetic Agent or Anesthetic Agents or Agents, Anesthetic or Anesthetic Effect or Effect, Anesthetic or Anesthetic Effects or Effects, Anesthetic or Isoflurane or Desflurane or Sevoflurane or Halothane or Nitrous Oxide or Oxide, Nitrous or Propofol or Zeneca Brand of Propofol or Astra Brand of Propofol or AstraZeneca Brand of Propofol or Alpha Brand of Propofol or Juste Brand of Propofol or Propofol Fresenius or Fresenius Kabi Brand of Propofol or Propofol MCT or Fresenius Brand of Propofol or Propofol Rovi or Rovi Brand of Propofol or Propofol-Lipuro or Braun Brand of Propofol or Pisa Brand of Propofol or Schering Brand of Propofol or Curamed Brand of Propofol or Parnell Brand of Propofol or Propofol Abbott or Abbott Brand of Propofol or Hypnotics and Sedatives or Sedatives and Hypnotics or Hypnotic Effect or Effect, Hypnotic or Hypnotic Effects or Effects, Hypnotic or Sedatives or Hypnotics or Sedative Effect or Effect, Sedative or Sedative Effects or Effects, Sedative or Thiopental or Rhone Merieux Brand of Thiopental Sodium or Merial Brand of Thiopental Sodium or Abbott Brand of Thiopental Sodium or Pisa Brand of Thiopental Sodium or Thiopental Nycomed or Nycomed Brand of Thiopental Sodium or Thiopental Sodium or Braun Brand of Thiopental Sodium or Altana Pharma Brand of Thiopental Sodium or Pharmtech Brand of Thiopental Sodium or GABA Modulators or Modulators, GABA or GABAergic Modulators or Modulators, GABAergic or Gamma-Aminobutyric Acid Modulators or Acid Modulators, gamma-Aminobutyric or Modulators, gamma-Aminobutyric Acid or Gamma Aminobutyric Acid Modulators or GABA-A Receptor Agonists or Agonists, GABA-A Receptor or GABA A Receptor Agonists or Receptor Agonists, GABA-A or GABA-A Receptor Agonist or Agonist, GABA-A Receptor or GABA A Receptor Agonist or Receptor Agonist, GABA-A or GABA-A Agonists or Agonists, GABA-A or GABA A Agonists or Agonists, GABA or gamma-Aminobutyric Acid Agonists or Acid Agonists, gamma-Aminobutyric or Agonists, gamma-Aminobutyric Acid or Gamma Aminobutyric Acid Agonists or GABA Receptor Agonists or Agonists, GABA Receptor or Receptor Agonists, GABA or Gamma-Aminobutyric Acid Agonist or Acid Agonist, gamma-Aminobutyric or Agonist, gamma-Aminobutyric Acid or Gamma Aminobutyric Acid Agonist or GABA Agonist or Agonist, GABA or GABA Receptor Agonist or Agonist, GABA Receptor or Receptor Agonist, GABA or Ketamine or Ketamine Hydrochloride or Excitatory Amino Acid Antagonists or Antagonists, Excitatory Amino Acid or Amino Acids,

© 2022 Reighard C et al. *JAMA Network Open.*

Excitatory, Antagonists or Glutamate Receptor Antagonists or Antagonists, Glutamate Receptor or Receptor Antagonists, Glutamate or EAA Antagonists or Antagonists, EAA or Glutamate Antagonists or Antagonists, Glutamate or Amino Acid Antagonists, Excitatory

#21 #6 or #7 or #8 or #9 or #10 or #11 or #12 or #13 or #14 or #15 or #16 or #17 or #18 or #19 or #20

#22 MeSH descriptor: [Cognition] explode all trees

#23 MeSH descriptor: [Cognition Disorders] explode all trees

#24 MeSH descriptor: [Neurodevelopmental Disorders] explode all trees

#25 MeSH descriptor: [Developmental Disabilities] explode all trees

#26 MeSH descriptor: [Neurobehavioral Manifestations] explode all trees

#27 Disorder, Cognition or Disorders, Cognition or Cognition or Disorder, Mental or Disorders, Mental or Mental Disorder or Behavior Disorders or Disorders, Behavior or Mental Disorders, Severe or Disorder, Severe Mental or Disorders, Severe Mental or Mental Disorder, Severe or Severe Mental Disorder or Severe Mental Disorders or Cognitive Impairments or Cognitive Impairment or Impairment, Cognitive or Impairments, Cognitive or Cognitive Deficits or Cognitive Deficit or Deficit, Cognitive or Deficits, Cognitive or Cognitive Defects or Cognitive Defect or Defect, Cognitive or Defects, Cognitive or Specific Learning Disorder or Disorder, Specific Learning or Learning Disorder, Specific or Learning Disorders, Specific or Specific Learning Disorders or Neurodevelopmental Disorders or Disorder, Neurodevelopmental or Disorders, Neurodevelopmental or Neurodevelopmental Disorder or Mental Disorders Diagnosed in Childhood or Disorders Usually Diagnosed in Infancy, Childhood or Adolescence or Child Mental Disorders or Child Mental Disorder or Disorder, Child Mental or Disorders, Child Mental or Mental Disorder, Child or Mental Disorders, Child or Developmental Disabilities or Disabilities, Developmental or Developmental Disability or Disability, Developmental or Developmental Delay Disorders or Developmental Delay Disorder or Neurobehavioral Manifestations or Manifestation, Neurobehavioral or Manifestations, Neurobehavioral or Neurobehavioral Manifestation or Signs and Symptoms, Neurobehavioral or Cognitive Manifestations or Cognitive Manifestation or Manifestation, Cognitive or Manifestations, Cognitive or Neurobehavioral Signs and Symptoms

#28 #22 or #23 or #24 or #25 or #26 or #27

#29 #5 and #21 and #28

© 2022 Reighard C et al. *JAMA Network Open.*

eTable 1. All Domains and Subdomains Evaluated in Studies of Potential Neurotoxic Effects of Anesthetic

Domain	Subdomains
Academics	Math
	Writing
	Reading
	Spelling
Adaptive behavior	Social
	Community participation and independence
	Problem solving
	Daily living skills
	Personal/social
Behavioral problems	Externalizing problems
	Internalizing problems
	Social problems
	Thought problems
Cognition	
Clinical diagnoses and symptoms	Attention deficit hyperactivity disorder
	Autism
	Cerebral palsy
	Developmental delay
	Post-traumatic stress disorder
	Sleep
Executive function	Memory
	Flexibility
	Attention
	Inhibition
	Processing speed
General development	
General health and wellbeing	
Language	Verbal intelligence quotient
	Expressive language
	Receptive language
	Articulation
Motor function	Fine motor
	Gross motor
Nonverbal reasoning	
Sensory	
Social-cognition	

© 2022 Reighard C et al. *JAMA Network Open.*

eTable 2. All Outcomes Evaluated in Studies of Potential Neurotoxic Effects of Anesthetic and Their Associated Neurodevelopmental Domain and Subdomain Classifications

Test	Description of Score	Domain	Overall Measure or Subdomain
A-TAC	The Autism - Tics, ADHD and other Comorbidities Inventory: Autism Spectrum Disorder	Clinical diagnoses and symptoms	ASD
A-TAC	The Autism - Tics, ADHD and other Comorbidities Inventory: Learning Disabilities	Clinical diagnoses and symptoms	Developmental delay
A-TAC	The Autism - Tics, ADHD and other Comorbidities Inventory: Attention Deficit Hyperactivity Disorder	Clinical diagnoses and symptoms	ADHD
ABAS-II	Adaptive Behavior Assessment System-2nd edition Conceptual Composite Score	Adaptive behavior	Overall
ABAS-II	Adaptive Behavior Assessment System-2nd edition General Adaptive Composite Score	Adaptive behavior	Overall
ABAS-II	Adaptive Behavior Assessment System-2nd edition Social Composite Score	Adaptive behavior	Social
ABAS-II	Adaptive Behavior Assessment System-2nd edition Practical Composite Score	Adaptive behavior	Overall
ABAS-II	Adaptive Behavior Assessment System-2nd edition Motor Composite Score	Motor function	Overall
ABS	Adaptive Behavior Scale Conceptual	Adaptive behavior	Overall
ABS	Adaptive Behavior Scale General Adaptive Composite	Adaptive behavior	Overall
ABS	Adaptive Behavior Scale Practical	Adaptive behavior	Overall
ABS	Adaptive Behavior Scale Social	Adaptive behavior	Social
ADHD	Attention Deficit Hyperactivity Disorder Diagnosis	Clinical diagnoses and symptoms	ADHD
ADHD	ADHD medication use	Clinical diagnoses and symptoms	ADHD
ADHD RS IV	ADHD Rating Scale IV Preschool Version Inattention	Clinical diagnoses and symptoms	ADHD
ADHD RS IV	ADHD Rating Scale IV Preschool Version Impulsivity	Clinical diagnoses and symptoms	ADHD
AIMS	Alberta Infant Motor Scale	Motor function	Overall
Ambulation	Ability to ambulate	Motor function	Gross motor
ASD	Autism Spectrum Disorder Diagnoses	Clinical diagnoses and symptoms	ASD
ASQ	Ages & Stages Questionnaire Personal/Social	Adaptive behavior	Personal/social
ASQ	Ages & Stages Questionnaire Problem Solving	Adaptive behavior	Problem solving
ASQ	Ages & Stages Questionnaire Communication	Language	Overall
ASQ	Ages & Stages Questionnaire Fine Motor	Motor function	Fine motor
ASQ	Ages & Stages Questionnaire Gross Motor	Motor function	Gross motor
Attention (unspecified)	Attention: Spatial Forward	Executive function	Attention
AvEDI	Early Development Instrument (Australia): Language and Cognitive Development	Language	Unable to be mapped to a subdomain
AvEDI	Early Development Instrument (Australia version) Developmentally High Risk	General development	Overall

© 2022 Reighard C et al. *JAMA Network Open.*

AvEDI	Early Development Instrument (Australia): Physical Health and Well-Being	General health and wellbeing	No subdomains for this domain
AvEDI	Early Development Instrument (Australia): Communication Skills and General Knowledge	Language	Unable to be mapped to a subdomain
AvEDI	Early Development Instrument (Australia): Emotional Health and Maturity	Social-cognition	No subdomains for this domain
AvEDI	Early Development Instrument (Australia): Social Knowledge and Competence	Social-cognition	No subdomains for this domain
AVLT	Rey Auditory Verbal Learning Test Verbal Learning	Executive function	Memory
AVLT	Rey Auditory Verbal Learning Test Verbal Long-term Memory	Executive function	Memory
AVLT	Rey Auditory Verbal Learning Test Verbal Recognition Memory	Executive function	Memory
BDS	Backward Digit Span Test	Executive function	Memory
Beery-Buktenica	Beery-Buktenica Developmental Test of Visual Motor Integration	Motor function	Unable to be mapped to a subdomain
Beery-Buktenica	Beery-Buktenica Developmental Test: Motor Coordination, Grooved Pegboard Dominant Hand	Motor function	Fine motor
Beery-Buktenica	Beery-Buktenica Developmental Test: Visual Perception, Judgment of Line Orientation	Motor function	Fine motor
Blindness	Blindness Diagnosis	Clinical diagnoses and symptoms	Vision
BNT	Boston Naming Test	Language	Overall
Bourdon-vos	Bourdon-vos test	Executive function	Attention
BPVS	British Picture Vocabulary Scale	Language	Receptive language
BRIEF GEC	Behavior Rating Inventory of the Executive Functions Global Executive Composite	Executive function	Overall
BRIEF-P GEC	Behavior Rating Inventory of Executive Function, Preschool version Global Executive Composite	Executive function	Overall
BSID-II MDI	Bayley Scales of Infant Development-2nd edition Mental Development Index	Cognition	Overall
BSID-II PDI	Bayley Scales of Infant Development-2nd edition Psychomotor Development Index	Motor function	Overall
BSID-III	Bayley Scales of Infant Development-3rd edition Functional Pre-Academics	Academics	Overall
BSID-III	Bayley Scales of Infant Development-3rd edition Community Use	Adaptive behavior	Community participation and independence
BSID-III	Bayley Scales of Infant Development-3rd edition Home Living	Adaptive behavior	Daily living skills
BSID-III	Bayley Scales of Infant Development-3rd edition Health and Safety	Adaptive behavior	Community participation and independence
BSID-III	Bayley Scales of Infant Development-3rd edition Leisure	Adaptive behavior	Community participation and independence
BSID-III	Bayley Scales of Infant Development-3rd edition Self-Care	Adaptive behavior	Daily living skills
BSID-III	Bayley Scales of Infant Development-3rd edition Self-Direction	Adaptive behavior	Daily living skills
BSID-III	Bayley Scales of Infant Development-3rd edition Adaptive Behavior	Adaptive behavior	Overall
BSID-III	Bayley Scales of Infant Development-3rd edition Cognition Scaled/Composite	Cognition	Overall

© 2022 Reighard C et al. *JAMA Network Open.*

BSID-III	Bayley Scales of Infant Development-3rd edition Receptive Language	Language	Receptive language
BSID-III	Bayley Scales of Infant Development-3rd edition Expressive Language	Language	Expressive language
BSID-III	Bayley Scales of Infant Development-3rd edition Receptive and Expressive Language Composite	Language	Overall
BSID-III	Bayley Scales of Infant Development-3rd edition Communication	Language	Overall
BSID-III	Bayley Scales of Infant Development-3rd edition Fine Motor	Motor function	Fine motor
BSID-III	Bayley Scales of Infant Development-3rd edition Gross Motor	Motor function	Gross motor
BSID-III	Bayley Scales of Infant Development-3rd edition Motor Composite	Motor function	Overall
BSID-III	Bayley Scales of Infant Development-3rd edition Social-Emotional	Social-cognition	No subdomains for this domain
BSID-III	Bayley Scales of Infant Development-3rd edition Social	Social-cognition	No subdomains for this domain
CAT	California Achievement Test Math	Academics	Math
CAT	California Achievement Test Phonics	Academics	Reading
CAT	California Achievement Test Spelling	Academics	Spelling
CAT	California Achievement Test Written Language	Academics	Writing
CBCL	Child Behavior Checklist Aggressive Behavior	Behavioral problems	Externalizing problems
CBCL	Child Behavior Checklist Anxious/Depressed	Behavioral problems	Internalizing problems
CBCL	Child Behavior Checklist Delinquent Behavior	Behavioral problems	Unable to be mapped to a subdomain
CBCL	Child Behavior Checklist Emotionally Reactive	Behavioral problems	Internalizing problems
CBCL	Child Behavior Checklist Externalizing Problems	Behavioral problems	Externalizing problems
CBCL	Child Behavior Checklist Internalizing Problems	Behavioral problems	Internalizing problems
CBCL	Child Behavior Checklist Total Problems	Behavioral problems	Overall
CBCL	Child Behavior Checklist Somatic Complaints	Behavioral problems	Internalizing problems
CBCL	Child Behavior Checklist Social Problems	Behavioral problems	Social problems
CBCL	Child Behavior Checklist Thought Problems	Behavioral problems	Thought
CBCL	Child Behavior Checklist Withdrawn	Behavioral problems	Internalizing problems
CBCL	Child Behavior Checklist ADHD Problems	Clinical diagnoses and symptoms	ADHD
CBCL	Child Behavior Checklist Attention Problems	Clinical diagnoses and symptoms	ADHD
CBCL	Child Behavior Checklist Pervasive Developmental Problem Scale	Clinical diagnoses and symptoms	ASD
CBCL	Child Behavior Checklist Sleep Problems	Clinical diagnoses and symptoms	Sleep
CDI	Children's Depression Inventory	Behavioral problems	Internalizing problems
CELF	Clinical Evaluation of Language Fundamentals-Expressive Language Score	Language	Expressive language
CELF	Clinical Evaluation of Language Fundamentals-Receptive Language Score	Language	Receptive language
CELF	Clinical Evaluation of Language Fundamentals-Total Language Score	Language	Overall

© 2022 Reighard C et al. *JAMA Network Open.*

Cerebral Palsy	Cerebral Palsy Diagnosis	Clinical diagnoses and symptoms	Cerebral palsy
Child's Communication Checklist	Child's Communication Checklist	Language	Overall
CHQ	Child Health Questionnaire	General health and wellbeing	No subdomains for this domain
CHQ50	The Child Health Questionnaire 50 General Behavior	Behavioral problems	Overall
CHQ50	The Child Health Questionnaire 50 Bodily Pain	General health and wellbeing	Overall
CHQ50	The Child Health Questionnaire 50 Change of Health	General health and wellbeing	Overall
CHQ50	The Child Health Questionnaire 50 General Health	General health and wellbeing	Overall
CHQ50	The Child Health Questionnaire 50 Physical Functioning	General health and wellbeing	Overall
CHQ50	The Child Health Questionnaire 50 Family Activities	Unable to be mapped to a domain	Unable to be mapped to a subdomain
CHQ50	The Child Health Questionnaire 50 Family Cohesion	Unable to be mapped to a domain	Unable to be mapped to a subdomain
CHQ50	The Child Health Questionnaire 50 Parental Emotional Impact	Unable to be mapped to a domain	Unable to be mapped to a subdomain
CHQ50	The Child Health Questionnaire 50 Parental Time Impact	General health and wellbeing	No subdomains for this domain
CHQ50	The Child Health Questionnaire 50 Mental Health	Social-cognition	Overall
CHQ50	The Child Health Questionnaire 50 Self-esteem	Social-cognition	Overall
CHQ50	The Child Health Questionnaire 50 Social-Emotional Role	Social-cognition	Overall
CHQ50	The Child Health Questionnaire 50 Social-Physical Role	Social-cognition	Overall
CLDQ	Colorado Learning Difficulties Questionnaire Math Scale	Academics	Math
CLDQ	Colorado Learning Difficulties Questionnaire Reading Scale	Academics	Reading
CMS	Children's Memory Scale Numbers	Executive function	Memory
CMS	Children's Memory Scale Word Lists I	Executive function	Memory
CMS	Children's Memory Scale Word Lists II	Executive function	Memory
Cons IQ	Conscription IQ Test	Cognition	Overall
Counting span	Counting span task	Executive function	Memory
CPM	Raven's Colored Progressive Matrices	Nonverbal reasoning	Overall
CPRI	Child Post-Traumatic Stress Disorder Reaction Index	Clinical diagnoses and symptoms	PTSD
CPT2	Conner's Continuous Performance Test II Detectability	Executive function	Inhibition
CPT2	Conner's Continuous Performance Test II Hit Reaction Time	Executive function	Inhibition
CPT2	Conner's Continuous Performance Test II Number Commissions	Executive function	Inhibition
CPT2	Conner's Continuous Performance Test II Number Omissions	Executive function	Inhibition
CPT2	Conner's Continuous Performance Test II Preservations	Executive function	Inhibition

© 2022 Reighard C et al. *JAMA Network Open.*

CPT2	Conner's Continuous Performance Test II Variability	Executive function	Inhibition
CTOPP	Comprehensive Test of Phonological Processing: Rapid Naming Composite	Language	Overall
CTRS-R:S	Conner's' teacher Rating Scale-Revised: Short Form	Behavioral problems	Externalizing problems
CVLT-C	California Verbal Learning Test-Children Total Trials 1-5	Executive function	Memory
DAS-II	Differential Abilities Scale Second Edition	Cognition	Overall
Developmental Delay	Developmental Delay (overall, language, motor, social, and/or behavioral) diagnosis	Clinical diagnoses and symptoms	Developmental delay
DKEFS	Delis-Kaplan Executive Function System Trail Making Test Condition 1	Executive function	Flexibility
DKEFS	Delis-Kaplan Executive Function System Trail Making Test Condition 2	Executive function	Flexibility
DKEFS	Delis-Kaplan Executive Function System Trail Making Test Condition 3	Executive function	Flexibility
DKEFS	Delis-Kaplan Executive Function System Trail Making Test Condition 4	Executive function	Flexibility
DKEFS	Delis-Kaplan Executive Function System Trail Making Test Condition 5	Executive function	Flexibility
DKEFS	Delis-Kaplan Executive Function System Tower Test Total Achievement Score	Executive function	Flexibility
DKEFS	Delis-Kaplan Executive Function System Verbal Fluency: Category Fluency	Executive function	Flexibility
DSM IV	Diagnostic and Statistical Manual of Mental Disorders-4th Edition Disorder Diagnosis, 3-point scale	Clinical diagnoses and symptoms	Overall
Dyscalculia	Dyscalculia diagnosis	Clinical diagnoses and symptoms	Unable to be mapped to a subdomain
Dyslexia	Dyslexia diagnosis	Clinical diagnoses and symptoms	Unable to be mapped to a subdomain
ECBI	Eyberg Child Behavior Inventory	Behavioral problems	Externalizing problems
EDI	Early Development Instrument Language and Cognitive Development	General development	No subdomains for this domain
EDI	Early Development Instrument Total Score	General development	Overall
EDI	Early Development Instrument Multiple Challenge Index	General development	No subdomains for this domain
EDI	Early Development Instrument Early Developmental Vulnerability	General development	Overall
EDI	Early Development Instrument Physical Health and Well-Being	General health and wellbeing	No subdomains for this domain
EDI	Early Development Instrument Communication Skills and General Knowledge	Language	Overall
EDI	Early Development Instrument Emotional Health and Maturity	Social-cognition	No subdomains for this domain
EDI	Early Development Instrument Social Knowledge and Competence	Social-cognition	No subdomains for this domain
Educational Attainment	Educational Attainment	Academics	Overall
Executive Function (unspecified)	Color-Word Inhibition	Executive function	Inhibition
Executive Function (unspecified)	Color-Word Inhibition Switching	Executive function	Inhibition

© 2022 Reighard C et al. *JAMA Network Open.*

Executive Function (unspecified)	Number-letter Sequencing	Executive function	Processing speed
Executive Function (unspecified)	20 Questions	Unable to be mapped to a domain	Unable to be mapped to a subdomain
Expressive Language	Expressive language composite (Boston naming + D-KEFS)	Language	Expressive language
FDS	Forward Digit Span Test	Executive function	Memory
Fine motor	Fine motor composite (Beery Motor + grooved pegboard)	Motor function	Fine motor
G-TVPS	Gardner Test of Visual-Perceptual Skills Revised: Visual Memory Test	Executive function	Memory
GDS	Gesell Developmental Schedule Language	Language	Overall
GDS	Gesell Developmental Schedule Response to Objects	Unable to be mapped to a domain	Unable to be mapped to a subdomain
GDS	Gesell Developmental Schedule Response to People	Unable to be mapped to a domain	Unable to be mapped to a subdomain
GDS	Gesell Developmental Schedule Movement	Motor function	Overall
GMDS	Griffiths Mental Development Scale Performance	Adaptive behavior	Problem solving
GMDS	Griffiths Mental Development Scale Personal/Social	Adaptive behavior	Personal/social
GMDS	Griffiths Mental Development Scale Mental Age	Cognition	Overall
GMDS	Griffiths Mental Development Scale General Development Quotient	General development	Overall
GMDS	Griffiths Mental Development Scale Hearing and Speech	Language	Overall
GMDS	Griffiths Mental Development Scale Eye/Hand	Motor function	Fine motor
GMDS	Griffiths Mental Development Scale Motor	Motor function	Gross motor
GMDS-II	Griffiths Mental Development Scale II General Development Quotient Deficiency	General development	Overall
GMFCS	Gross Motor Function Classification System	Clinical diagnoses and symptoms	Cerebral palsy
Go/no go	Go/No go Task	Executive function	Inhibition
GPT	Grooved Pegboard Test Dominant Hand	Motor function	Fine motor
GPT	Grooved Pegboard Test Fine Motor	Motor function	Fine motor
GPT	Grooved Pegboard Test Non-Dominant Hand	Motor function	Fine motor
HAWIVA-III	Hannover-Wechsler Intelligence Scale, 3rd edition Full Scale IQ	Cognition	Overall
HAWIVA-III	Hannover-Wechsler Intelligence Scale, 3rd Processing Speed	Executive function	Processing speed
HAWIVA-III	Hannover-Wechsler Intelligence Scale, 3rd edition Verbal IQ	Language	Verbal IQ
HAWIVA-III	Hannover-Wechsler Intelligence Scale, 3rd edition Nonverbal IQ	Nonverbal reasoning	Overall
Hearing	Hearing Impairment	Clinical diagnoses and symptoms	Hearing
HK-WISC	Hong Kong-Wechsler Intelligence Scale for Children General Comprehension	Language	Verbal IQ
HKLL	Hong Kong List Learning	Executive function	Memory
IEP	Need for Individualized Education Plan	Clinical diagnoses and symptoms	Developmental delay

© 2022 Reighard C et al. *JAMA Network Open.*

IEP	Individualized Education Plan Speech/Language	Clinical diagnoses and symptoms	Developmental delay
IEP	Individualized Education Plan Emotion/Behavioral	Clinical diagnoses and symptoms	Developmental delay
Iowa	Iowa Overall Standardized Test	Academics	Overall
ITSP	Infant/toddler Sensory Profile Auditory Processing	Sensory	No subdomains for this domain
ITSP	Infant/toddler Sensory Profile Low Threshold	Sensory	No subdomains for this domain
ITSP	Infant/toddler Sensory Profile Oral Processing	Sensory	No subdomains for this domain
ITSP	Infant/toddler Sensory Profile Low Registration	Sensory	No subdomains for this domain
ITSP	Infant/toddler Sensory Profile Sensation Seeking	Sensory	No subdomains for this domain
ITSP	Infant/toddler Sensory Profile Sensory Sensitivity	Sensory	No subdomains for this domain
ITSP	Infant/toddler Sensory Profile Sensation Avoiding	Sensory	No subdomains for this domain
ITSP	Infant/toddler Sensory Profile Tactile Processing	Sensory	No subdomains for this domain
ITSP	Infant/toddler Sensory Profile Vestibular Processing	Sensory	No subdomains for this domain
ITSP	Infant/toddler Sensory Profile Visual Processing	Sensory	No subdomains for this domain
J-ASQ-3	Japanese Ages and Stages Questionnaire, Third edition Communication	Language	Overall
J-ASQ-3	Japanese Ages and Stages Questionnaire, Third edition Gross motor	Motor function	Gross motor
J-ASQ-3	Japanese Ages and Stages Questionnaire, Third edition Fine motor	Motor function	Fine motor
J-ASQ-3	Japanese Ages and Stages Questionnaire, Third edition Problem solving	Adaptive behavior	Problem solving
J-ASQ-3	Japanese Ages and Stages Questionnaire, Third edition Personal-social	Adaptive behavior	Personal/social
K-ABC	Kaufmann Assessment Battery for Children Location Memory	Executive function	Memory
K-ABC	Kaufmann Assessment Battery for Children Picture	Nonverbal reasoning	No subdomains for this domain
K-ABC	Kaufmann Assessment Battery for Children Triangle	Nonverbal reasoning	No subdomains for this domain
K-ABC	Kaufmann Assessment Battery for Children Visual Analogy	Nonverbal reasoning	No subdomains for this domain
KET-KID	Kognitiver Enwicklungstest fur das Kindergartenalter Global Score	Cognition	Overall
KET-KID	Kognitiver Enwicklungstest fur das Kindergartenalter Verbal Memory	Executive function	Memory
KET-KID	Kognitiver Enwicklungstest fur das Kindergartenalter Visual Memory	Executive function	Memory
KET-KID	Kognitiver Enwicklungstest fur das Kindergartenalter Articulation	Language	Articulation
KET-KID	Kognitiver Enwicklungstest fur das Kindergartenalter Verbal Development	Language	Overall
KET-KID	Kognitiver Enwicklungstest fur das Kindergartenalter Verbal Comprehension	Language	Receptive language

© 2022 Reighard C et al. *JAMA Network Open.*

KET-KID	Kognitiver Enwicklungstest fur das Kindergartenalter Psychomotor Development	Motor function	Overall
KET-KID	Kognitiver Enwicklungstest fur das Kindergartenalter Nonverbal Development	Nonverbal reasoning	No subdomains for this domain
KET-KID	Kognitiver Enwicklungstest fur das Kindergartenalter Visual Perception	Nonverbal reasoning	No subdomains for this domain
KET-KID	Kognitiver Enwicklungstest fur das Kindergartenalter Visuoconstruction	Nonverbal reasoning	No subdomains for this domain
Key Stage 2	Key Stage 2 English	Academics	Unable to be mapped to a subdomain
Key Stage 2	Key Stage 2 Mathematics	Academics	Math
Key Stage 2	Key Stage 2 Science	Academics	Unable to be mapped to a subdomain
Key Stage 2	Key Stage 2 non-attainment	Academics	Unable to be mapped to a subdomain
Key Stage 3	Key Stage 3 English	Academics	Unable to be mapped to a subdomain
Key Stage 3	Key Stage 3 Mathematics	Academics	Math
Key Stage 3	Key Stage 3 Science	Academics	Unable to be mapped to a subdomain
Key Stage 3	Key Stage 3 non-attainment	Academics	Unable to be mapped to a subdomain
Key Stage 4	Key Stage 4 total points	Academics	Unable to be mapped to a subdomain
Key Stage 4	Key Stage 4 exam entries	Academics	Unable to be mapped to a subdomain
Key Stage 4	Key Stage 4 English A*, A, b, or C grade	Academics	Unable to be mapped to a subdomain
Key Stage 4	Key Stage 4 mathematics A*, A, b, or C grade	Academics	Math
Key Stage 4	Key Stage 4 science 2 “good” passes (C grade or above)	Academics	Unable to be mapped to a subdomain
LD	Learning Disability/Disorder Diagnosis	Clinical diagnoses and symptoms	Developmental delay
LD	Learning Disability/Disorder Diagnosis - Reading	Clinical diagnoses and symptoms	Developmental delay
LD	Learning Disability/Disorder Diagnosis - Writing	Clinical diagnoses and symptoms	Developmental delay
LD	Learning Disability/Disorder Diagnosis - Math	Clinical diagnoses and symptoms	Developmental delay
M-ABC	Movement Assessment Battery for Children Total Score	Motor function	Overall
M-ABC	Movement Assessment Battery for Children Balance Skills	Motor function	Gross motor
M-ABC	Movement Assessment Battery for Children Ball Skills	Motor function	Gross motor
M-ABC	Movement Assessment Battery for Children Manual Dexterity	Motor function	Fine motor
M-ABC	Movement Assessment Battery for Children Heel-to-toe walking task	Motor function	Gross motor
M-ABC	Movement Assessment Battery for Children Preferred hand peg placing	Motor function	Fine motor
M-ABC	Movement Assessment Battery for Children Nonpreferred hand peg placing	Motor function	Fine motor
M-ABC	Movement Assessment Battery for Children Bean bag throwing task	Motor function	Gross motor

© 2022 Reighard C et al. *JAMA Network Open.*

MAND	McCarren Assessment of Neuromuscular Development	Motor function	Overall
McArthur--Bates	McArthur Bates Communicative Development Inventory	Language	Overall
Mental Disorders	Mental Disorder Diagnosis	Clinical diagnoses and symptoms	Overall
NAPLAN	New South Wales Department of Education National Assessment Program-Literacy and Numeracy: Numeracy	Academics	Math
NAPLAN	New South Wales Department of Education National Assessment Program-Literacy and Numeracy: Reading	Academics	Reading
National Test	National Standardized Test Score	Academics	Overall
NEPSY-II	Developmental Neuropsychological Assessment Battery-2nd Edition Auditory Attention	Executive function	Attention
NEPSY-II	Developmental Neuropsychological Assessment Battery-2nd Edition Delayed Memory for Faces	Executive function	Memory
NEPSY-II	Developmental Neuropsychological Assessment Battery-2nd Edition Fingertap Tapping Repetitions	Executive function	Inhibition
NEPSY-II	Developmental Neuropsychological Assessment Battery-2nd Edition Fingertap Tapping Sequences	Executive function	Inhibition
NEPSY-II	Developmental Neuropsychological Assessment Battery-2nd Edition Inhibition	Executive function	Inhibition
NEPSY-II	Developmental Neuropsychological Assessment Battery-2nd Edition Memory for Faces	Executive function	Memory
NEPSY-II	Developmental Neuropsychological Assessment Battery-2nd Edition Memory for Names	Executive function	Memory
NEPSY-II	Developmental Neuropsychological Assessment Battery-2nd Edition Sentence Repetition	Executive function	Memory
NEPSY-II	Developmental Neuropsychological Assessment Battery-2nd Edition Speeded Naming	Executive function	Processing speed
NEPSY-II	Developmental Neuropsychological Assessment Battery-2nd Edition Statue	Executive function	Inhibition
NEPSY-II	Developmental Neuropsychological Assessment Battery-2nd Edition Word Generation	Executive function	Processing speed
NEPSY-II	Developmental Neuropsychological Assessment Battery-2nd Edition Comprehension of Instructions	Language	Receptive language
NEPSY-II	Developmental Neuropsychological Assessment Battery-2nd Edition Design Copy	Nonverbal reasoning	No subdomains for this domain
NEPSY-II	Developmental Neuropsychological Assessment Battery-2nd Edition Affect Recognition	Social-cognition	No subdomains for this domain
NEPSY-II	Developmental Neuropsychological Assessment Battery-2nd Edition Theory of Mind	Social-cognition	No subdomains for this domain
Neuro	Neurological Diagnoses	Clinical diagnoses and symptoms	Overall
Neurodevelopmental disorder	Language/behavioral/psychomotor disorder	Clinical diagnoses and symptoms	Unable to be mapped to a subdomain
OLSAT	Stanford/Otis-Lennon School Ability Test Mathematics	Academics	Math
OLSAT	Stanford/Otis-Lennon School Ability Test Reading	Academics	Reading
OLSAT	Stanford/Otis-Lennon School Ability Test Spelling	Academics	Spelling
OLSAT	Stanford/Otis-Lennon School Ability Test Total Battery	Academics	Overall
OLSAT	Stanford/Otis-Lennon School Ability Test Language	Language	Overall

© 2022 Reighard C et al. *JAMA Network Open.*

OTB	Operant Test Battery Conditioned Position Response	Executive function	Unable to be mapped to a subdomain
OTB	Operant Test Battery Delayed Match to Sample	Executive function	Unable to be mapped to a subdomain
OTB	Operant Test Battery Incremental Repeated Acquisition	Executive function	Unable to be mapped to a subdomain
OTB	Operant Test Battery Progressive Ratio	Executive function	Unable to be mapped to a subdomain
OTB	Operant Test Battery Temporal Response Differentiation	Executive function	Unable to be mapped to a subdomain
OWLS	Oral and Written Language Scales	Language	Unable to be mapped to a subdomain
Parent Questionnaire	Parent Social-Emotional Questionnaire	Social-cognition	No subdomains for this domain
PDMS	Peabody Developmental Motor Scales	Motor function	Overall
Phoneme deletion	Phoneme deletion task	Language	Unable to be mapped to a subdomain
PPVT	Peabody Picture Vocabulary Test	Language	Receptive language
Processing Speed (unspecified)	Processing speed: Digit Symbol	Executive function	Processing speed
Processing Speed (unspecified)	Processing Speed: Color Naming	Executive function	Processing speed
Processing Speed (unspecified)	Processing Speed: Dominant Hand Speed	Executive function	Processing speed
Processing Speed (unspecified)	Processing Speed: Letter Sequencing	Executive function	Processing speed
Processing Speed (unspecified)	Processing Speed: Non-dominant Hand Speed	Executive function	Processing speed
Processing Speed (unspecified)	Processing Speed: Number Sequencing	Executive function	Processing speed
Processing Speed (unspecified)	Processing Speed: Verbal Fluency	Executive function	Processing speed
PSLE	Primary School Leaving Examination	Academics	Overall
Psychiatric Diagnosis	Psychiatric Diagnoses	Clinical diagnoses and symptoms	Overall
Psychiatric Interview	Psychiatric Interview	Clinical diagnoses and symptoms	Overall
Psychologist	Education psychologist counseling	Clinical diagnoses and symptoms	Developmental delay
PVF	Phonemic verbal fluency test	Executive function	Processing speed
RAKIT	Revised Amsterdam Intelligence Test	Cognition	Overall
RCFT	Rey Complex Figure Test and Recognition Trial: Copy	Executive function	Memory
RDLS	Reynell Developmental Language Scales: Comprehension	Language	Receptive language
RDLS	Reynell Developmental Language Scales: Expressive Language	Language	Expressive language
Reading	Real-word reading test	Academics	Reading
Reading	Non–real-word reading test	Academics	Reading
Recognition Memory	Recognition memory: Color Familiarity	Executive function	Memory

© 2022 Reighard C et al. *JAMA Network Open.*

Recognition Memory	Recognition Memory: Color Recollection	Executive function	Memory
Recognition Memory	Recognition Memory: Spatial Familiarity	Executive function	Memory
Recognition Memory	Recognition Memory: Spatial Recollection	Executive function	Memory
RSPM	Raven's Standard Progressive Matrices: IQ	Nonverbal reasoning	Overall
SAT Math	Vermont School SAT Math	Academics	Math
SAT Reading	Vermont School SAT Reading	Academics	Reading
SB-V	Stanford-Binet V FSIQ	Cognition	Overall
SB-V	Stanford-Binet V VIQ	Language	Verbal IQ
SB-V	Stanford-Binet V Fluid Reasoning	Unable to be mapped to a domain	Unable to be mapped to a subdomain
SB-V	Stanford-Binet V Knowledge	Unable to be mapped to a domain	Unable to be mapped to a subdomain
SB-V	Stanford-Binet V Quantitative Reasoning	Unable to be mapped to a domain	Unable to be mapped to a subdomain
SB-V	Stanford-Binet V Visual-spatial Processing	Unable to be mapped to a domain	Unable to be mapped to a subdomain
SB-V	Stanford-Binet V Working Memory	Executive function	Unable to be mapped to a subdomain
SB-V	Stanford-Binet V Nonverbal IQ	Nonverbal reasoning	No subdomains for this domain
School	School Performance or Grades	Academics	Overall
SDMT	Symbol Digit Modality Test: Oral	Executive function	Processing speed
SDMT	Symbol Digit Modality Test: Written	Executive function	Processing speed
SDQ	Strengths and Difficulties Questionnaire	Behavioral problems	Overall
Skuse sociocognitive dysfunction	Skuse sociocognitive dysfunction score	Behavioral problems	Social problems
Social-Emotional	Social Emotional Scale	Social-cognition	
SON-R	Hogrefe/Snijders-Oomen Non-Verbal Intelligence Test-Revised IQ	Nonverbal reasoning	Overall
Spelling (unspecified)	Spelling test	Academics	Spelling
SVF	Semantic Verbal Fluency Test	Executive function	Processing speed
TCS	Test of Cognitive Skills Total Cognitive	Cognition	Overall
TCS	Test of Cognitive Skills Memory	Executive function	Memory
TEA-Ch	Test of Everyday Attention for Children Sky search task	Executive function	Attention
TEA-Ch	Test of Everyday Attention for Children Opposite worlds task	Executive function	Attention
TEA-Ch NL	Test of Everyday Attention for Children, Dutch Version Response Inhibition	Executive function	Inhibition
TEA-Ch NL	Test of Everyday Attention for Children, Dutch Version Sustained Auditory Attention	Executive function	Attention
TEA-Ch NL	Test of Everyday Attention for Children, Dutch Version Visual Attention	Executive function	Attention
Teacher	Teacher Rating	Academics	Overall
Test	Standardized Test Score Non-Attainment	Academics	Overall

© 2022 Reighard C et al. *JAMA Network Open.*

TOWRE	Test of Word Reading Efficiency word-reading test	Academics	Reading
TOWRE	Test of Word Reading Efficiency non-word–reading test	Academics	Reading
Treatment for neurodevelopmenta l disorder	Intervention for neurodevelopmental disorder	Clinical diagnoses and symptoms	Unable to be mapped to a subdomain
VABS-I	Vineland Adaptive Behavior Scales, 1st edition Deficit in subscale	Adaptive behavior	Overall
VABS-I	Vineland Adaptive Behavior Scales, 1st edition Daily Living Skills	Adaptive behavior	Daily living skills
VABS-I	Vineland Adaptive Behavior Scales, 1st edition Socialization	Adaptive behavior	Social
VABS-II	Vineland Adaptive Behavior Scales, 2nd edition Composite	Adaptive behavior	Overall
VABS-II	Vineland Adaptive Behavior Scales, 2nd edition Daily Living	Adaptive behavior	Daily living skills
VABS-II	Vineland Adaptive Behavior Scales, 2nd edition Socialization	Adaptive behavior	Social
VABS-II	Vineland Adaptive Behavior Scales, 2nd edition Total Sum	Adaptive behavior	Overall
VABS-II	Vineland Adaptive Behavior Scales, 2nd edition Communication Standard	Language	Overall
VABS-II	Vineland Adaptive Behavior Scales, 2nd edition Motor Skills	Motor function	Overall
VPAA	Very Poor Academic Performance Reading	Academics	Reading
VPAA	Very Poor Academic Performance Math	Academics	Math
VPAA	Very Poor Academic Performance Overall	Academics	Overall
WAIS-III VIQ	Wechsler Adult Intelligence Scale 3rd edition Verbal IQ	Language	Overall
WAIS-IV	Wechsler Adult Intelligence Scale Version IV Full Scale Intelligence Quotient	Cognition	Overall
WAIS-IV	Wechsler Adult Intelligence Scale Version IV Verbal Comprehension	Language	Verbal IQ
WAIS-IV	Wechsler Adult Intelligence Scale Version IV Perceptual Reasoning	Non-verbal reasoning	No subdomains for this domain
WAIS-IV	Wechsler Adult Intelligence Scale Version IV Working Memory	Executive function	Memory
WAIS-IV	Wechsler Adult Intelligence Scale Version IV Processing Speed	Executive function	Processing speed
Wallin	Wallin B pegboard from the Merrill-Palmer Scale of Mental Test	Motor function	Fine motor
WAMSE	Western Australian Literacy and Numeracy Standardized Test Numeracy	Academics	Math
WAMSE	Western Australian Literacy and Numeracy Standardized Test Reading	Academics	Reading
WAMSE	Western Australian Literacy and Numeracy Standardized Test Spelling	Academics	Spelling
WAMSE	Western Australian Literacy and Numeracy Standardized Test Writing	Academics	Writing
WASI	Wechsler Abbreviated Scale of Intelligence Full Scale IQ	Cognition	Overall
WASI	Wechsler Abbreviated Scale of Intelligence Similarities	Language	Verbal IQ
WASI	Wechsler Abbreviated Scale of Intelligence Verbal IQ	Language	Verbal IQ
WASI	Wechsler Abbreviated Scale of Intelligence Vocabulary	Language	Verbal IQ

© 2022 Reighard C et al. *JAMA Network Open.*

WASI	Wechsler Abbreviated Scale of Intelligence Matrix Vocab	Unable to be mapped to a domain	Unable to be mapped to a subdomain
WASI	Wechsler Abbreviated Scale of Intelligence Block Design	Nonverbal reasoning	No subdomains for this domain
WASI	Wechsler Abbreviated Scale of Intelligence Matrix Reasoning	Nonverbal reasoning	No subdomains for this domain
WASI	Wechsler Abbreviated Scale of Intelligence Performance IQ	Nonverbal reasoning	Overall
WCST	Wisconsin Card Sort Test: Perseverative Errors	Executive function	Inhibition
WCST	Wisconsin Card Sort Test: Perseverative Responses	Executive function	Inhibition
WeeFIM	WeeFIM System for Functional Independence	Adaptive behavior	Daily living skills
WIAT-II	Wechsler Individual Achievement Test-2nd Edition Numerical Composite	Academics	Math
WIAT-II	Wechsler Individual Achievement Test-2nd Edition Spelling	Academics	Spelling
WIAT-II	Wechsler Individual Achievement Test-2nd Edition Word Reading	Academics	Reading
WISC-III	Wechsler Intelligence Scale for Children-3rd edition Full Scale Intelligence Quotient	Cognition	Overall
WISC-III	Wechsler Intelligence Scale for Children-3rd edition Verbal Intelligence Quotient	Language	Verbal IQ
WISC-III	Wechsler Intelligence Scale for Children-3rd edition Performance Intelligence Quotient	Nonverbal reasoning	Overall
WISC-III-NL	Wechsler Intelligence Scale for Children-3rd edition, Dutch version FSIQ	Cognition	Overall
WISC-III-NL	Wechsler Intelligence Scale for Children-3rd edition, Dutch version Verbal IQ	Language	Verbal IQ
WISC-III-NL	Wechsler Intelligence Scale for Children-3rd edition, Dutch version Performance IQ	Nonverbal reasoning	Overall
WISC-IV	Wechsler Intelligence Scale for Children-4th edition IQ	Cognition	Overall
WISC-IV	Wechsler Intelligence Scale for Children-4th edition Coding	Executive function	Processing speed
WISC-IV	Wechsler Intelligence Scale for Children-4th edition Digit Span	Executive function	Memory
WJ	Woodcock-Johnson Visual Matching Test	Executive function	Processing speed
WJ-III	Woodcock-Johnson Achievement Battery III Mathematics	Academics	Math
WJ-III	Woodcock-Johnson Achievement Battery III Reading	Academics	Reading
WJ-III ACA	Woodcock Johnson III Tests of Academic Achievement Broad Reading	Academics	Reading
WJ-III COG	Woodcock Johnson III Tests of Cognitive Abilities General Intellectual Ability/IQ	Cognition	Overall
WJ-III COG	Woodcock Johnson III Tests of Cognitive Abilities Broad Attention	Executive function	Attention
WJ-III COG	Woodcock Johnson III Tests of Cognitive Abilities Processing Speed	Executive function	Processing speed
WJ-III COG	Woodcock Johnson III Tests of Cognitive Abilities Working Memory	Executive function	Memory
WMS--CR	Wechsler Memory Scale-Chinese Revision Digit Span	Executive function	Memory
WMS--CR	Wechsler Memory Scale-Chinese Revision Image Recalling	Executive function	Memory
WMS--CR	Wechsler Memory Scale-Chinese Revision Figure Recognition	Executive function	Memory

© 2022 Reighard C et al. *JAMA Network Open.*

WMS--CR	Wechsler Memory Scale-Chinese Revision Visual Reproduction	Executive function	Memory
WMS--CR	Wechsler Memory Scale-Chinese Revision Verbal-Paired Associates	Executive function	Memory
WMS--CR	Wechsler Memory Scale-Chinese Revision Comprehension Memory	Executive function	Memory
WMS--CR	Wechsler Memory Scale-Chinese Revision Forward Counting	Executive function	Memory
WMS--CR	Wechsler Memory Scale-Chinese Revision Accumulation	Executive function	Memory
WMS--CR	Wechsler Memory Scale-Chinese Revision Backward Counting	Executive function	Memory
WOLD	Wechsler Objective Language Dimensions comprehension task	Language	Receptive language
WOLD	Wechsler Objective Language Dimensions verbal expression task	Language	Expressive language
WORD	Wechsler Objective Reading Dimensions Basic reading test	Academics	Reading
WPPSI-III	Wechsler Preschool and Primary Scale of Intelligence-3rd Edition Arithmetic	Academics	Math
WPPSI-III	Wechsler Preschool and Primary Scale of Intelligence-3rd Edition FSIQ	Cognition	Overall
WPPSI-III	Wechsler Preschool and Primary Scale of Intelligence-3rd Edition Animal House	Executive function	Processing speed
WPPSI-III	Wechsler Preschool and Primary Scale of Intelligence-3rd Edition Processing Speed	Executive function	Processing speed
WPPSI-III	Wechsler Preschool and Primary Scale of Intelligence-3rd Edition Comprehension	Language	Verbal IQ
WPPSI-III	Wechsler Preschool and Primary Scale of Intelligence-3rd Edition Information	Language	Verbal IQ
WPPSI-III	Wechsler Preschool and Primary Scale of Intelligence-3rd Edition Similarities	Language	Verbal IQ
WPPSI-III	Wechsler Preschool and Primary Scale of Intelligence-3rd Edition VIQ	Language	Overall
WPPSI-III	Wechsler Preschool and Primary Scale of Intelligence-3rd Edition Vocabulary	Language	Unable to be mapped to a subdomain
WPPSI-III	Wechsler Preschool and Primary Scale of Intelligence-3rd Edition Block Design	Nonverbal reasoning	No subdomains for this domain
WPPSI-III	Wechsler Preschool and Primary Scale of Intelligence-3rd Edition Geometric Design	Nonverbal reasoning	No subdomains for this domain
WPPSI-III	Wechsler Preschool and Primary Scale of Intelligence-3rd Edition Mazes	Nonverbal reasoning	No subdomains for this domain
WPPSI-III	Wechsler Preschool and Primary Scale of Intelligence-3rd Edition PIQ	Nonverbal reasoning	Overall
WPPSI-III	Wechsler Preschool and Primary Scale of Intelligence-3rd Edition Picture Completion	Nonverbal reasoning	No subdomains for this domain
WPPSI-IV (CR)	Wechsler Preschool and Primary Scale of Intelligence, fourth edition, Chinese version FSIQ	Cognition	Overall
WPPSI-IV (CR)	Wechsler Preschool and Primary Scale of Intelligence, fourth edition, Chinese version Verbal Comprehension Index	Language	Verbal IQ
WPPSI-IV (CR)	Wechsler Preschool and Primary Scale of Intelligence, fourth edition, Chinese version Visual-Spatial Index	Unable to be mapped to a domain	Unable to be mapped to a subdomain
WPPSI-IV (CR)	Wechsler Preschool and Primary Scale of Intelligence, fourth edition, Chinese version Fluid Reasoning Index	Unable to be mapped to a domain	Unable to be mapped to a subdomain

© 2022 Reighard C et al. *JAMA Network Open.*

WPPSI-IV (CR)	Wechsler Preschool and Primary Scale of Intelligence, fourth edition, Chinese version Working Memory Index	Executive function	Memory
WPPSI-IV (CR)	Wechsler Preschool and Primary Scale of Intelligence, fourth edition, Chinese version Processing Speed Index	Executive function	Processing speed
WPPSI-R	Wechsler Preschool and Primary Scale of Intelligence-Revised IQ	Cognition	Overall
WRAML-2	Wide Range Assessment of Memory and Learning, second edition Attention/concentration Index	Executive function	Attention
WRAML-2	Wide Range Assessment of Memory and Learning, second edition Delayed Verbal Recall Composite	Executive function	Memory
WRAML-2	Wide Range Assessment of Memory and Learning, second edition Design Memory	Executive function	Memory
WRAML-2	Wide Range Assessment of Memory and Learning, second edition Design Recognition	Executive function	Memory
WRAML-2	Wide Range Assessment of Memory and Learning, second edition Story Memory Delay Recall	Executive function	Memory
WRAML-2	Wide Range Assessment of Memory and Learning, second edition Story Memory Recognition	Executive function	Memory
WRAML-2	Wide Range Assessment of Memory and Learning, second edition Verbal Learning Delay Recall	Executive function	Memory
WRAML-2	Wide Range Assessment of Memory and Learning, second edition Verbal Learning Recognition	Executive function	Memory
WRAML-2	Wide Range Assessment of Memory and Learning, second edition Verbal Memory Index	Executive function	Memory
WRAML-2	Wide Range Assessment of Memory and Learning, second edition Verbal Recognition Composite	Executive function	Memory

eTable 3. All Neurodevelopmental Domain and Subdomains and the Classification of Outcomes into These Domains and Subdomains

Domain	Overall Measure or Subdomain	Test	Description of Score
Academics	Overall	BSID-III	Bayley Scales of Infant Development-3rd edition Functional Pre-Academics
Academics	Overall	Educational Attainment	Educational Attainment
Academics	Overall	Iowa	Iowa Overall Standardized Test
Academics	Overall	National Test	National Standardized Test Score
Academics	Overall	OLSAT	Stanford/Otis-Lennon School Ability Test Total Battery
Academics	Overall	PSLE	Primary School Leaving Examination
Academics	Overall	School	School Performance or Grades
Academics	Overall	Teacher	Teacher Rating
Academics	Overall	Test	Standardized Test Score Non-Attainment
Academics	Overall	VPAA	Very Poor Academic Performance Overall
Academics	Math	CAT	California Achievement Test Math
Academics	Math	CLDQ	Colorado Learning Difficulties Questionnaire Math Scale
Academics	Math	Key Stage 2	Key Stage 2 Mathematics

© 2022 Reighard C et al. *JAMA Network Open.*

Academics	Math	Key Stage 3	Key Stage 3 Mathematics
Academics	Math	Key Stage 4	Key Stage 4 mathematics A*, A, b, or C grade
Academics	Math	NAPLAN	New South Wales Department of Education National Assessment Program-Literacy and Numeracy: Numeracy
Academics	Math	OLSAT	Stanford/Otis-Lennon School Ability Test Mathematics
Academics	Math	SAT Math	Vermont School SAT Math
Academics	Math	VPAA	Very Poor Academic Performance Math
Academics	Math	WAMSE	Western Australian Literacy and Numeracy Standardized Test Numeracy
Academics	Math	WIAT-II	Wechsler Individual Achievement Test-2nd Edition Numerical Composite
Academics	Math	WJ-III	Woodcock-Johnson Achievement Battery III Mathematics
Academics	Math	WPPSI-III	Wechsler Preschool and Primary Scale of Intelligence-3rd Edition Arithmetic
Academics	Reading	CAT	California Achievement Test Phonics
Academics	Reading	CLDQ	Colorado Learning Difficulties Questionnaire Reading Scale
Academics	Reading	NAPLAN	New South Wales Department of Education National Assessment Program-Literacy and Numeracy: Reading
Academics	Reading	OLSAT	Stanford/Otis-Lennon School Ability Test Reading
Academics	Reading	Reading	Real-word reading test
Academics	Reading	Reading	Non–real-word reading test
Academics	Reading	SAT Reading	Vermont School SAT Reading
Academics	Reading	TOWRE	Test of Word Reading Efficiency word-reading test
Academics	Reading	TOWRE	Test of Word Reading Efficiency non-word–reading test
Academics	Reading	VPAA	Very Poor Academic Performance Reading
Academics	Reading	WAMSE	Western Australian Literacy and Numeracy Standardized Test Reading
Academics	Reading	WIAT-II	Wechsler Individual Achievement Test-2nd Edition Word Reading
Academics	Reading	WJ-III	Woodcock-Johnson Achievement Battery III Reading
Academics	Reading	WJ-III ACA	Woodcock Johnson III Tests of Academic Achievement Broad Reading
Academics	Reading	WORD	Wechsler Objective Reading Dimensions Basic reading test
Academics	Spelling	CAT	California Achievement Test Spelling
Academics	Spelling	OLSAT	Stanford/Otis-Lennon School Ability Test Spelling
Academics	Spelling	Spelling (unspecified)	Spelling test
Academics	Spelling	WAMSE	Western Australian Literacy and Numeracy Standardized Test Spelling
Academics	Spelling	WIAT-II	Wechsler Individual Achievement Test-2nd Edition Spelling
Academics	Unable to be mapped to a subdomain	Key Stage 2	Key Stage 2 English
Academics	Unable to be mapped to a subdomain	Key Stage 2	Key Stage 2 Science
Academics	Unable to be mapped to a subdomain	Key Stage 2	Key Stage 2 non-attainment

Academics	Unable to be mapped to a subdomain	Key Stage 3	Key Stage 3 English
Academics	Unable to be mapped to a subdomain	Key Stage 3	Key Stage 3 Science
Academics	Unable to be mapped to a subdomain	Key Stage 3	Key Stage 3 non-attainment
Academics	Unable to be mapped to a subdomain	Key Stage 4	Key Stage 4 total points
Academics	Unable to be mapped to a subdomain	Key Stage 4	Key Stage 4 exam entries
Academics	Unable to be mapped to a subdomain	Key Stage 4	Key Stage 4 English A*, A, b, or C grade
Academics	Unable to be mapped to a subdomain	Key Stage 4	Key Stage 4 science 2 “good” passes (C grade or above)
Academics	Writing	CAT	California Achievement Test Written Language
Academics	Writing	WAMSE	Western Australian Literacy and Numeracy Standardized Test Writing
Adaptive behavior	Overall	ABAS-II	Adaptive Behavior Assessment System-2nd edition Conceptual Composite Score
Adaptive behavior	Overall	ABAS-II	Adaptive Behavior Assessment System-2nd edition General Adaptive Composite Score
Adaptive behavior	Overall	ABAS-II	Adaptive Behavior Assessment System-2nd edition Practical Composite Score
Adaptive behavior	Overall	ABS	Adaptive Behavior Scale Conceptual
Adaptive behavior	Overall	ABS	Adaptive Behavior Scale General Adaptive Composite
Adaptive behavior	Overall	ABS	Adaptive Behavior Scale Practical
Adaptive behavior	Overall	BSID-III	Bayley Scales of Infant Development-3rd edition Adaptive Behavior
Adaptive behavior	Overall	VABS-I	Vineland Adaptive Behavior Scales, 1st edition Deficit in subscale
Adaptive behavior	Overall	VABS-II	Vineland Adaptive Behavior Scales, 2nd edition Composite
Adaptive behavior	Overall	VABS-II	Vineland Adaptive Behavior Scales, 2nd edition Total Sum
Adaptive behavior	Community participation and independence	BSID-III	Bayley Scales of Infant Development-3rd edition Community Use
Adaptive behavior	Community participation and independence	BSID-III	Bayley Scales of Infant Development-3rd edition Health and Safety
Adaptive behavior	Community participation and independence	BSID-III	Bayley Scales of Infant Development-3rd edition Leisure
Adaptive behavior	Daily living skills	BSID-III	Bayley Scales of Infant Development-3rd edition Home Living
Adaptive behavior	Daily living skills	BSID-III	Bayley Scales of Infant Development-3rd edition Self-Care
Adaptive behavior	Daily living skills	BSID-III	Bayley Scales of Infant Development-3rd edition Self-Direction
Adaptive behavior	Daily living skills	VABS-I	Vineland Adaptive Behavior Scales, 1st edition Daily Living Skills
Adaptive behavior	Daily living skills	VABS-II	Vineland Adaptive Behavior Scales, 2nd edition Daily Living
Adaptive behavior	Daily living skills	WeeFIM	WeeFIM System for Functional Independence

© 2022 Reighard C et al. *JAMA Network Open.*

Adaptive behavior	Personal/social	ASQ	Ages & Stages Questionnaire Personal/Social
Adaptive behavior	Personal/social	GMDS	Griffiths Mental Development Scale Personal/Social
Adaptive behavior	Personal/social	J-ASQ-3	Japanese Ages and Stages Questionnaire, Third edition Personal-social
Adaptive behavior	Problem solving	ASQ	Ages & Stages Questionnaire Problem Solving
Adaptive behavior	Problem solving	GMDS	Griffiths Mental Development Scale Performance
Adaptive behavior	Problem solving	J-ASQ-3	Japanese Ages and Stages Questionnaire, Third edition Problem solving
Adaptive behavior	Social	ABAS-II	Adaptive Behavior Assessment System-2nd edition Social Composite Score
Adaptive behavior	Social	ABS	Adaptive Behavior Scale Social
Adaptive behavior	Social	VABS-I	Vineland Adaptive Behavior Scales, 1st edition Socialization
Adaptive behavior	Social	VABS-II	Vineland Adaptive Behavior Scales, 2nd edition Socialization
Behavioral problems	Overall	CBCL	Child Behavior Checklist Total Problems
Behavioral problems	Overall	CHQ50	The Child Health Questionnaire 50 General Behavior
Behavioral problems	Overall	SDQ	Strengths and Difficulties Questionnaire
Behavioral problems	Externalizing problems	CBCL	Child Behavior Checklist Aggressive Behavior
Behavioral problems	Externalizing problems	CBCL	Child Behavior Checklist Externalizing Problems
Behavioral problems	Externalizing problems	CTRS-R:S	Conner's' teacher Rating Scale-Revised: Short Form
Behavioral problems	Externalizing problems	ECBI	Eyberg Child Behavior Inventory
Behavioral problems	Internalizing problems	CBCL	Child Behavior Checklist Anxious/Depressed
Behavioral problems	Internalizing problems	CBCL	Child Behavior Checklist Emotionally Reactive
Behavioral problems	Internalizing problems	CBCL	Child Behavior Checklist Internalizing Problems
Behavioral problems	Internalizing problems	CBCL	Child Behavior Checklist Somatic Complaints
Behavioral problems	Internalizing problems	CBCL	Child Behavior Checklist Withdrawn
Behavioral problems	Internalizing problems	CDI	Children's Depression Inventory
Behavioral problems	Social problems	CBCL	Child Behavior Checklist Social Problems
Behavioral problems	Social problems	Skuse sociocognitive dysfunction	Skuse sociocognitive dysfunction score
Behavioral problems	Thought	CBCL	Child Behavior Checklist Thought Problems
Behavioral problems	Unable to be mapped to a subdomain	CBCL	Child Behavior Checklist Delinquent Behavior
Clinical diagnoses and symptoms	Overall	DSM IV	Diagnostic and Statistical Manual of Mental Disorders-4th Edition Disorder Diagnosis, 3-point scale
Clinical diagnoses and symptoms	Overall	Mental Disorders	Mental Disorder Diagnosis
Clinical diagnoses and symptoms	Overall	Neuro	Neurological Diagnoses
Clinical diagnoses and symptoms	Overall	Psychiatric Diagnosis	Psychiatric Diagnoses

© 2022 Reighard C et al. *JAMA Network Open.*

Clinical diagnoses and symptoms	Overall	Psychiatric Interview	Psychiatric Interview
Clinical diagnoses and symptoms	ADHD	ADHD	Attention Deficit Hyperactivity Disorder Diagnosis
Clinical diagnoses and symptoms	ADHD	ADHD	ADHD medication use
Clinical diagnoses and symptoms	ADHD	CBCL	Child Behavior Checklist ADHD Problems
Clinical diagnoses and symptoms	ADHD	CBCL	Child Behavior Checklist Attention Problems
Clinical diagnoses and symptoms	ASD	A-TAC	The Autism - Tics, ADHD and other Comorbidities Inventory: Autism Spectrum Disorder
Clinical diagnoses and symptoms	ASD	ASD	Autism Spectrum Disorder Diagnoses
Clinical diagnoses and symptoms	ASD	CBCL	Child Behavior Checklist Pervasive Developmental Problem Scale
Clinical diagnoses and symptoms	Cerebral palsy	Cerebral Palsy	Cerebral Palsy Diagnosis
Clinical diagnoses and symptoms	Cerebral palsy	GMFCS	Gross Motor Function Classification System
Clinical diagnoses and symptoms	Developmental delay	A-TAC	The Autism - Tics, ADHD and other Comorbidities Inventory: Learning Disabilities
Clinical diagnoses and symptoms	Developmental delay	Developmental Delay	Developmental Delay (overall, language, motor, social, and/or behavioral) diagnosis
Clinical diagnoses and symptoms	Developmental delay	IEP	Need for Individualized Education Plan
Clinical diagnoses and symptoms	Developmental delay	IEP	Individualized Education Plan Speech/Language
Clinical diagnoses and symptoms	Developmental delay	IEP	Individualized Education Plan Emotion/Behavioral
Clinical diagnoses and symptoms	Developmental delay	LD	Learning Disability/Disorder Diagnosis
Clinical diagnoses and symptoms	Developmental delay	LD	Learning Disability/Disorder Diagnosis - Reading
Clinical diagnoses and symptoms	Developmental delay	LD	Learning Disability/Disorder Diagnosis - Writing
Clinical diagnoses and symptoms	Developmental delay	LD	Learning Disability/Disorder Diagnosis - Math
Clinical diagnoses and symptoms	Developmental delay	Psychologist	Education psychologist counseling
Clinical diagnoses and symptoms	Hearing	Hearing	Hearing Impairment
Clinical diagnoses and symptoms	PTSD	CPRI	Child Post-Traumatic Stress Disorder Reaction Index
Clinical diagnoses and symptoms	Sleep	CBCL	Child Behavior Checklist Sleep Problems
Clinical diagnoses and symptoms	Unable to be mapped to a subdomain	Dyscalculia	Dyscalculia diagnosis
Clinical diagnoses and symptoms	Unable to be mapped to a subdomain	Dyslexia	Dyslexia diagnosis
Clinical diagnoses and symptoms	Unable to be mapped to a subdomain	Neurodevelopmental disorder	Language/behavioral/psychomotor disorder
Clinical diagnoses and symptoms	Unable to be mapped to a subdomain	Treatment for neurodevelopmental disorder	Intervention for neurodevelopmental disorder

© 2022 Reighard C et al. *JAMA Network Open.*

Clinical diagnoses and symptoms	ADHD	A-TAC	The Autism - Tics, ADHD and other Comorbidities Inventory: Attention Deficit Hyperactivity Disorder
Clinical diagnoses and symptoms	ADHD	ADHD RS IV	ADHD Rating Scale IV Preschool Version Inattention
Clinical diagnoses and symptoms	ADHD	ADHD RS IV	ADHD Rating Scale IV Preschool Version Impulsivity
Clinical diagnoses and symptoms	Vision	Blindness	Blindness Diagnosis
Cognition	Overall	BSID-II MDI	Bayley Scales of Infant Development-2nd edition Mental Development Index
Cognition	Overall	BSID-III	Bayley Scales of Infant Development-3rd edition Cognition Scaled/Composite
Cognition	Overall	Cons IQ	Conscription IQ Test
Cognition	Overall	DAS-II	Differential Abilities Scale Second Edition
Cognition	Overall	GMDS	Griffiths Mental Development Scale Mental Age
Cognition	Overall	HAWIVA-III	Hannover-Wechsler Intelligence Scale, 3rd edition Full Scale IQ
Cognition	Overall	KET-KID	Kognitiver Enwicklungstest fur das Kindergartenalter Global Score
Cognition	Overall	RAKIT	Revised Amsterdam Intelligence Test
Cognition	Overall	SB-V	Stanford-Binet V FSIQ
Cognition	Overall	TCS	Test of Cognitive Skills Total Cognitive
Cognition	Overall	WAIS-IV	Wechsler Adult Intelligence Scale Version IV Full Scale Intelligence Quotient
Cognition	Overall	WASI	Wechsler Abbreviated Scale of Intelligence Full Scale IQ
Cognition	Overall	WISC-III	Wechsler Intelligence Scale for Children-3rd edition Full Scale Intelligence Quotient
Cognition	Overall	WISC-III-NL	Wechsler Intelligence Scale for Children-3rd edition, Dutch version FSIQ
Cognition	Overall	WISC-IV	Wechsler Intelligence Scale for Children-4th edition IQ
Cognition	Overall	WJ-III COG	Woodcock Johnson III Tests of Cognitive Abilities General Intellectual Ability/IQ
Cognition	Overall	WPPSI-III	Wechsler Preschool and Primary Scale of Intelligence-3rd Edition FSIQ
Cognition	Overall	WPPSI-IV (CR)	Wechsler Preschool and Primary Scale of Intelligence, fourth edition, Chinese version FSIQ
Cognition	Overall	WPPSI-R	Wechsler Preschool and Primary Scale of Intelligence-Revised IQ
Executive function	Overall	BRIEF GEC	Behavior Rating Inventory of the Executive Functions Global Executive Composite
Executive function	Overall	BRIEF-P GEC	Behavior Rating Inventory of Executive Function, Preschool version Global Executive Composite
Executive function	Attention	Attention (unspecified)	Attention: Spatial Forward
Executive function	Attention	Bourdon-vos	Bourdon-vos test
Executive function	Attention	NEPSY-II	Developmental Neuropsychological Assessment Battery-2nd Edition Auditory Attention
Executive function	Attention	TEA-Ch	Test of Everyday Attention for Children Sky search task
Executive function	Attention	TEA-Ch	Test of Everyday Attention for Children Opposite worlds task
Executive function	Attention	TEA-Ch NL	Test of Everyday Attention for Children, Dutch Version Sustained Auditory Attention

© 2022 Reighard C et al. *JAMA Network Open.*

Executive function	Attention	TEA-Ch NL	Test of Everyday Attention for Children, Dutch Version Visual Attention
Executive function	Attention	WJ-III COG	Woodcock Johnson III Tests of Cognitive Abilities Broad Attention
Executive function	Attention	WRAML-2	Wide Range Assessment of Memory and Learning, second edition Attention/concentration Index
Executive function	Flexibility	DKEFS	Delis-Kaplan Executive Function System Trail Making Test Condition 1
Executive function	Flexibility	DKEFS	Delis-Kaplan Executive Function System Trail Making Test Condition 2
Executive function	Flexibility	DKEFS	Delis-Kaplan Executive Function System Trail Making Test Condition 3
Executive function	Flexibility	DKEFS	Delis-Kaplan Executive Function System Trail Making Test Condition 4
Executive function	Flexibility	DKEFS	Delis-Kaplan Executive Function System Trail Making Test Condition 5
Executive function	Flexibility	DKEFS	Delis-Kaplan Executive Function System Tower Test Total Achievement Score
Executive function	Flexibility	DKEFS	Delis-Kaplan Executive Function System Verbal Fluency: Category Fluency
Executive function	Inhibition	CPT2	Conner's Continuous Performance Test II Detectability
Executive function	Inhibition	CPT2	Conner's Continuous Performance Test II Hit Reaction Time
Executive function	Inhibition	CPT2	Conner's Continuous Performance Test II Number Commissions
Executive function	Inhibition	CPT2	Conner's Continuous Performance Test II Number Omissions
Executive function	Inhibition	CPT2	Conner's Continuous Performance Test II Preservations
Executive function	Inhibition	CPT2	Conner's Continuous Performance Test II Variability
Executive function	Inhibition	Executive Function (unspecified)	Color-Word Inhibition
Executive function	Inhibition	Executive Function (unspecified)	Color-Word Inhibition Switching
Executive function	Inhibition	Go/no go	Go/No go Task
Executive function	Inhibition	NEPSY-II	Developmental Neuropsychological Assessment Battery-2nd Edition Fingertap Tapping Repetitions
Executive function	Inhibition	NEPSY-II	Developmental Neuropsychological Assessment Battery-2nd Edition Fingertap Tapping Sequences
Executive function	Inhibition	NEPSY-II	Developmental Neuropsychological Assessment Battery-2nd Edition Inhibition
Executive function	Inhibition	NEPSY-II	Developmental Neuropsychological Assessment Battery-2nd Edition Statue
Executive function	Inhibition	TEA-Ch NL	Test of Everyday Attention for Children, Dutch Version Response Inhibition
Executive function	Inhibition	WCST	Wisconsin Card Sort Test: Perseverative Errors
Executive function	Inhibition	WCST	Wisconsin Card Sort Test: Perseverative Responses
Executive function	Memory	AVLT	Rey Auditory Verbal Learning Test Verbal Learning
Executive function	Memory	AVLT	Rey Auditory Verbal Learning Test Verbal Long-term Memory
Executive function	Memory	AVLT	Rey Auditory Verbal Learning Test Verbal Recognition Memory
Executive function	Memory	BDS	Backward Digit Span Test
Executive function	Memory	CMS	Children's Memory Scale Numbers

© 2022 Reighard C et al. *JAMA Network Open.*

Executive function	Memory	CMS	Children's Memory Scale Word Lists I
Executive function	Memory	CMS	Children's Memory Scale Word Lists II
Executive function	Memory	Counting span	Counting span task
Executive function	Memory	CVLT-C	California Verbal Learning Test-Children Total Trials 1-5
Executive function	Memory	FDS	Forward Digit Span Test
Executive function	Memory	G-TVPS	Gardner Test of Visual-Perceptual Skills Revised: Visual Memory Test
Executive function	Memory	HKLL	Hong Kong List Learning
Executive function	Memory	K-ABC	Kaufmann Assessment Battery for Children Location Memory
Executive function	Memory	KET-KID	Kognitiver Enwicklungstest fur das Kindergartenalter Verbal Memory
Executive function	Memory	KET-KID	Kognitiver Enwicklungstest fur das Kindergartenalter Visual Memory
Executive function	Memory	NEPSY-II	Developmental Neuropsychological Assessment Battery-2nd Edition Delayed Memory for Faces
Executive function	Memory	NEPSY-II	Developmental Neuropsychological Assessment Battery-2nd Edition Memory for Faces
Executive function	Memory	NEPSY-II	Developmental Neuropsychological Assessment Battery-2nd Edition Memory for Names
Executive function	Memory	NEPSY-II	Developmental Neuropsychological Assessment Battery-2nd Edition Sentence Repetition
Executive function	Memory	RCFT	Rey Complex Figure Test and Recognition Trial: Copy
Executive function	Memory	Recognition Memory	Recognition memory: Color Familiarity
Executive function	Memory	Recognition Memory	Recognition Memory: Color Recollection
Executive function	Memory	Recognition Memory	Recognition Memory: Spatial Familiarity
Executive function	Memory	Recognition Memory	Recognition Memory: Spatial Recollection
Executive function	Memory	TCS	Test of Cognitive Skills Memory
Executive function	Memory	WAIS-IV	Wechsler Adult Intelligence Scale Version IV Working Memory
Executive function	Memory	WISC-IV	Wechsler Intelligence Scale for Children-4th edition Digit Span
Executive function	Memory	WJ-III COG	Woodcock Johnson III Tests of Cognitive Abilities Working Memory
Executive function	Memory	WMS--CR	Wechsler Memory Scale-Chinese Revision Digit Span
Executive function	Memory	WMS--CR	Wechsler Memory Scale-Chinese Revision Image Recalling
Executive function	Memory	WMS--CR	Wechsler Memory Scale-Chinese Revision Figure Recognition
Executive function	Memory	WMS--CR	Wechsler Memory Scale-Chinese Revision Visual Reproduction
Executive function	Memory	WMS--CR	Wechsler Memory Scale-Chinese Revision Verbal-Paired Associates
Executive function	Memory	WMS--CR	Wechsler Memory Scale-Chinese Revision Comprehension Memory
Executive function	Memory	WMS--CR	Wechsler Memory Scale-Chinese Revision Forward Counting
Executive function	Memory	WMS--CR	Wechsler Memory Scale-Chinese Revision Accumulation
Executive function	Memory	WMS--CR	Wechsler Memory Scale-Chinese Revision Backward Counting

© 2022 Reighard C et al. *JAMA Network Open.*

Executive function	Memory	WPPSI-IV (CR)	Wechsler Preschool and Primary Scale of Intelligence, fourth edition, Chinese version Working Memory Index
Executive function	Memory	WRAML-2	Wide Range Assessment of Memory and Learning, second edition Delayed Verbal Recall Composite
Executive function	Memory	WRAML-2	Wide Range Assessment of Memory and Learning, second edition Design Memory
Executive function	Memory	WRAML-2	Wide Range Assessment of Memory and Learning, second edition Design Recognition
Executive function	Memory	WRAML-2	Wide Range Assessment of Memory and Learning, second edition Story Memory Delay Recall
Executive function	Memory	WRAML-2	Wide Range Assessment of Memory and Learning, second edition Story Memory Recognition
Executive function	Memory	WRAML-2	Wide Range Assessment of Memory and Learning, second edition Verbal Learning Delay Recall
Executive function	Memory	WRAML-2	Wide Range Assessment of Memory and Learning, second edition Verbal Learning Recognition
Executive function	Memory	WRAML-2	Wide Range Assessment of Memory and Learning, second edition Verbal Memory Index
Executive function	Memory	WRAML-2	Wide Range Assessment of Memory and Learning, second edition Verbal Recognition Composite
Executive function	Processing speed	Executive Function (unspecified)	Number-letter Sequencing
Executive function	Processing speed	HAWIVA-III	Hannover-Wechsler Intelligence Scale, 3rd Processing Speed
Executive function	Processing speed	NEPSY-II	Developmental Neuropsychological Assessment Battery-2nd Edition Speeded Naming
Executive function	Processing speed	NEPSY-II	Developmental Neuropsychological Assessment Battery-2nd Edition Word Generation
Executive function	Processing speed	Processing Speed (unspecified)	Processing speed: Digit Symbol
Executive function	Processing speed	Processing Speed (unspecified)	Processing Speed: Color Naming
Executive function	Processing speed	Processing Speed (unspecified)	Processing Speed: Dominant Hand Speed
Executive function	Processing speed	Processing Speed (unspecified)	Processing Speed: Letter Sequencing
Executive function	Processing speed	Processing Speed (unspecified)	Processing Speed: Non-dominant Hand Speed
Executive function	Processing speed	Processing Speed (unspecified)	Processing Speed: Number Sequencing
Executive function	Processing speed	Processing Speed (unspecified)	Processing Speed: Verbal Fluency
Executive function	Processing speed	PVF	Phonemic verbal fluency test
Executive function	Processing speed	SDMT	Symbol Digit Modality Test: Oral
Executive function	Processing speed	SDMT	Symbol Digit Modality Test: Written
Executive function	Processing speed	SVF	Semantic Verbal Fluency Test
Executive function	Processing speed	WAIS-IV	Wechsler Adult Intelligence Scale Version IV Processing Speed
Executive function	Processing speed	WISC-IV	Wechsler Intelligence Scale for Children-4th edition Coding
Executive function	Processing speed	WJ	Woodcock-Johnson Visual Matching Test
Executive function	Processing speed	WJ-III COG	Woodcock Johnson III Tests of Cognitive Abilities Processing Speed
Executive function	Processing speed	WPPSI-III	Wechsler Preschool and Primary Scale of Intelligence-3rd Edition Animal House

© 2022 Reighard C et al. *JAMA Network Open.*

Executive function	Processing speed	WPPSI-III	Wechsler Preschool and Primary Scale of Intelligence-3rd Edition Processing Speed
Executive function	Processing speed	WPPSI-IV (CR)	Wechsler Preschool and Primary Scale of Intelligence, fourth edition, Chinese version Processing Speed Index
Executive function	Unable to be mapped to a subdomain	OTB	Operant Test Battery Conditioned Position Response
Executive function	Unable to be mapped to a subdomain	OTB	Operant Test Battery Delayed Match to Sample
Executive function	Unable to be mapped to a subdomain	OTB	Operant Test Battery Incremental Repeated Acquisition
Executive function	Unable to be mapped to a subdomain	OTB	Operant Test Battery Progressive Ratio
Executive function	Unable to be mapped to a subdomain	OTB	Operant Test Battery Temporal Response Differentiation
Executive function	Unable to be mapped to a subdomain	SB-V	Stanford-Binet V Working Memory
General development	Overall	AvEDI	Early Development Instrument (Australia version) Developmentally High Risk
General development	Overall	EDI	Early Development Instrument Total Score
General development	Overall	EDI	Early Development Instrument Early Developmental Vulnerability
General development	Overall	GMDS	Griffiths Mental Development Scale General Development Quotient
General development	Overall	GMDS-II	Griffiths Mental Development Scale II General Development Quotient Deficiency
General development	No subdomains for this domain	EDI	Early Development Instrument Language and Cognitive Development
General development	No subdomains for this domain	EDI	Early Development Instrument Multiple Challenge Index
General health and wellbeing	Overall	CHQ50	The Child Health Questionnaire 50 Bodily Pain
General health and wellbeing	Overall	CHQ50	The Child Health Questionnaire 50 Change of Health
General health and wellbeing	Overall	CHQ50	The Child Health Questionnaire 50 General Health
General health and wellbeing	Overall	CHQ50	The Child Health Questionnaire 50 Physical Functioning
General health and wellbeing	No subdomains for this domain	AvEDI	Early Development Instrument (Australia): Physical Health and Well-Being
General health and wellbeing	No subdomains for this domain	CHQ	Child Health Questionnaire
General health and wellbeing	No subdomains for this domain	CHQ50	The Child Health Questionnaire 50 Parental Time Impact
General health and wellbeing	No subdomains for this domain	EDI	Early Development Instrument Physical Health and Well-Being
Language	Overall	ASQ	Ages & Stages Questionnaire Communication
Language	Overall	BNT	Boston Naming Test
Language	Overall	BSID-III	Bayley Scales of Infant Development-3rd edition Receptive and Expressive Language Composite
Language	Overall	BSID-III	Bayley Scales of Infant Development-3rd edition Communication
Language	Overall	CELF	Clinical Evaluation of Language Fundamentals-Total Language Score

Language	Overall	Child's Communication Checklist	Child's Communication Checklist
Language	Overall	CTOPP	Comprehensive Test of Phonological Processing: Rapid Naming Composite
Language	Overall	EDI	Early Development Instrument Communication Skills and General Knowledge
Language	Overall	GDS	Gesell Developmental Schedule Language
Language	Overall	GMDS	Griffiths Mental Development Scale Hearing and Speech
Language	Overall	J-ASQ-3	Japanese Ages and Stages Questionnaire, Third edition Communication
Language	Overall	KET-KID	Kognitiver Enwicklungstest fur das Kindergartenalter Verbal Development
Language	Overall	McArthur--Bates	McArthur Bates Communicative Development Inventory
Language	Overall	OLSAT	Stanford/Otis-Lennon School Ability Test Language
Language	Overall	VABS-II	Vineland Adaptive Behavior Scales, 2nd edition Communication Standard
Language	Overall	WAIS-III VIQ	Wechsler Adult Intelligence Scale 3rd edition Verbal IQ
Language	Overall	WPPSI-III	Wechsler Preschool and Primary Scale of Intelligence-3rd Edition VIQ
Language	Articulation	KET-KID	Kognitiver Enwicklungstest fur das Kindergartenalter Articulation
Language	Expressive language	BSID-III	Bayley Scales of Infant Development-3rd edition Expressive Language
Language	Expressive language	CELF	Clinical Evaluation of Language Fundamentals-Expressive Language Score
Language	Expressive language	Expressive Language	Expressive language composite (Boston naming + D-KEFS)
Language	Expressive language	RDLS	Reynell Developmental Language Scales: Expressive Language
Language	Expressive language	WOLD	Wechsler Objective Language Dimensions verbal expression task
Language	Receptive language	BPVS	British Picture Vocabulary Scale
Language	Receptive language	BSID-III	Bayley Scales of Infant Development-3rd edition Receptive Language
Language	Receptive language	CELF	Clinical Evaluation of Language Fundamentals-Receptive Language Score
Language	Receptive language	KET-KID	Kognitiver Enwicklungstest fur das Kindergartenalter Verbal Comprehension
Language	Receptive language	NEPSY-II	Developmental Neuropsychological Assessment Battery-2nd Edition Comprehension of Instructions
Language	Receptive language	PPVT	Peabody Picture Vocabulary Test
Language	Receptive language	RDLS	Reynell Developmental Language Scales: Comprehension
Language	Receptive language	WOLD	Wechsler Objective Language Dimensions comprehension task
Language	Unable to be mapped to a subdomain	AvEDI	Early Development Instrument (Australia): Language and Cognitive Development
Language	Unable to be mapped to a subdomain	AvEDI	Early Development Instrument (Australia): Communication Skills and General Knowledge
Language	Unable to be mapped to a subdomain	OWLS	Oral and Written Language Scales

© 2022 Reighard C et al. *JAMA Network Open.*

Language	Unable to be mapped to a subdomain	Phoneme deletion	Phoneme deletion task
Language	Unable to be mapped to a subdomain	WPPSI-III	Wechsler Preschool and Primary Scale of Intelligence-3rd Edition Vocabulary
Language	Verbal IQ	HAWIVA-III	Hannover-Wechsler Intelligence Scale, 3rd edition Verbal IQ
Language	Verbal IQ	HK-WISC	Hong Kong-Wechsler Intelligence Scale for Children General Comprehension
Language	Verbal IQ	SB-V	Stanford-Binet V VIQ
Language	Verbal IQ	WAIS-IV	Wechsler Adult Intelligence Scale Version IV Verbal Comprehension
Language	Verbal IQ	WASI	Wechsler Abbreviated Scale of Intelligence Similarities
Language	Verbal IQ	WASI	Wechsler Abbreviated Scale of Intelligence Verbal IQ
Language	Verbal IQ	WASI	Wechsler Abbreviated Scale of Intelligence Vocabulary
Language	Verbal IQ	WISC-III	Wechsler Intelligence Scale for Children-3rd edition Verbal Intelligence Quotient
Language	Verbal IQ	WISC-III-NL	Wechsler Intelligence Scale for Children-3rd edition, Dutch version Verbal IQ
Language	Verbal IQ	WPPSI-III	Wechsler Preschool and Primary Scale of Intelligence-3rd Edition Comprehension
Language	Verbal IQ	WPPSI-III	Wechsler Preschool and Primary Scale of Intelligence-3rd Edition Information
Language	Verbal IQ	WPPSI-III	Wechsler Preschool and Primary Scale of Intelligence-3rd Edition Similarities
Language	Verbal IQ	WPPSI-IV (CR)	Wechsler Preschool and Primary Scale of Intelligence, fourth edition, Chinese version Verbal Comprehension Index
Motor function	Overall	ABAS-II	Adaptive Behavior Assessment System-2nd edition Motor Composite Score
Motor function	Overall	AIMS	Alberta Infant Motor Scale
Motor function	Overall	BSID-II PDI	Bayley Scales of Infant Development-2nd edition Psychomotor Development Index
Motor function	Overall	BSID-III	Bayley Scales of Infant Development-3rd edition Motor Composite
Motor function	Overall	GDS	Gesell Developmental Schedule Movement
Motor function	Overall	KET-KID	Kognitiver Enwicklungstest fur das Kindergartenalter Psychomotor Development
Motor function	Overall	M-ABC	Movement Assessment Battery for Children Total Score
Motor function	Overall	MAND	McCarren Assessment of Neuromuscular Development
Motor function	Overall	PDMS	Peabody Developmental Motor Scales
Motor function	Overall	VABS-II	Vineland Adaptive Behavior Scales, 2nd edition Motor Skills
Motor function	Fine motor	ASQ	Ages & Stages Questionnaire Fine Motor
Motor function	Fine motor	Beery-Buktenica	Beery-Buktenica Developmental Test: Motor Coordination, Grooved Pegboard Dominant Hand
Motor function	Fine motor	Beery-Buktenica	Beery-Buktenica Developmental Test: Visual Perception, Judgment of Line Orientation
Motor function	Fine motor	BSID-III	Bayley Scales of Infant Development-3rd edition Fine Motor
Motor function	Fine motor	Fine motor	Fine motor composite (Beery Motor + grooved pegboard)
Motor function	Fine motor	GMDS	Griffiths Mental Development Scale Eye/Hand

© 2022 Reighard C et al. *JAMA Network Open.*

Motor function	Fine motor	GPT	Grooved Pegboard Test Dominant Hand
Motor function	Fine motor	GPT	Grooved Pegboard Test Fine Motor
Motor function	Fine motor	GPT	Grooved Pegboard Test Non-Dominant Hand
Motor function	Fine motor	J-ASQ-3	Japanese Ages and Stages Questionnaire, Third edition Fine motor
Motor function	Fine motor	M-ABC	Movement Assessment Battery for Children Manual Dexterity
Motor function	Fine motor	M-ABC	Movement Assessment Battery for Children Preferred hand peg placing
Motor function	Fine motor	M-ABC	Movement Assessment Battery for Children Nonpreferred hand peg placing
Motor function	Fine motor	Wallin	Wallin B pegboard from the Merrill-Palmer Scale of Mental Test
Motor function	Gross motor	Ambulation	Ability to ambulate
Motor function	Gross motor	ASQ	Ages & Stages Questionnaire Gross Motor
Motor function	Gross motor	BSID-III	Bayley Scales of Infant Development-3rd edition Gross Motor
Motor function	Gross motor	GMDS	Griffiths Mental Development Scale Motor
Motor function	Gross motor	J-ASQ-3	Japanese Ages and Stages Questionnaire, Third edition Gross motor
Motor function	Gross motor	M-ABC	Movement Assessment Battery for Children Balance Skills
Motor function	Gross motor	M-ABC	Movement Assessment Battery for Children Ball Skills
Motor function	Gross motor	M-ABC	Movement Assessment Battery for Children Heel-to-toe walking task
Motor function	Gross motor	M-ABC	Movement Assessment Battery for Children Bean bag throwing task
Motor function	Unable to be mapped to a subdomain	Beery-Buktenica	Beery-Buktenica Developmental Test of Visual Motor Integration
Non-verbal reasoning	No subdomains for this domain	WAIS-IV	Wechsler Adult Intelligence Scale Version IV Perceptual Reasoning
Nonverbal reasoning	Overall	CPM	Raven's Colored Progressive Matrices
Nonverbal reasoning	Overall	HAWIVA-III	Hannover-Wechsler Intelligence Scale, 3rd edition Nonverbal IQ
Nonverbal reasoning	Overall	RSPM	Raven's Standard Progressive Matrices: IQ
Nonverbal reasoning	Overall	SON-R	Hogrefe/Snijders-Oomen Non-Verbal Intelligence Test-Revised IQ
Nonverbal reasoning	Overall	WASI	Wechsler Abbreviated Scale of Intelligence Performance IQ
Nonverbal reasoning	Overall	WISC-III	Wechsler Intelligence Scale for Children-3rd edition Performance Intelligence Quotient
Nonverbal reasoning	Overall	WISC-III-NL	Wechsler Intelligence Scale for Children-3rd edition, Dutch version Performance IQ
Nonverbal reasoning	Overall	WPPSI-III	Wechsler Preschool and Primary Scale of Intelligence-3rd Edition PIQ
Nonverbal reasoning	No subdomains for this domain	K-ABC	Kaufmann Assessment Battery for Children Picture
Nonverbal reasoning	No subdomains for this domain	K-ABC	Kaufmann Assessment Battery for Children Triangle
Nonverbal reasoning	No subdomains for this domain	K-ABC	Kaufmann Assessment Battery for Children Visual Analogy
Nonverbal reasoning	No subdomains for this domain	KET-KID	Kognitiver Enwicklungstest fur das Kindergartenalter Nonverbal Development

© 2022 Reighard C et al. *JAMA Network Open.*

Nonverbal reasoning	No subdomains for this domain	KET-KID	Kognitiver Enwicklungstest fur das Kindergartenalter Visual Perception
Nonverbal reasoning	No subdomains for this domain	KET-KID	Kognitiver Enwicklungstest fur das Kindergartenalter Visuoconstruction
Nonverbal reasoning	No subdomains for this domain	NEPSY-II	Developmental Neuropsychological Assessment Battery-2nd Edition Design Copy
Nonverbal reasoning	No subdomains for this domain	SB-V	Stanford-Binet V Nonverbal IQ
Nonverbal reasoning	No subdomains for this domain	WASI	Wechsler Abbreviated Scale of Intelligence Block Design
Nonverbal reasoning	No subdomains for this domain	WASI	Wechsler Abbreviated Scale of Intelligence Matrix Reasoning
Nonverbal reasoning	No subdomains for this domain	WPPSI-III	Wechsler Preschool and Primary Scale of Intelligence-3rd Edition Block Design
Nonverbal reasoning	No subdomains for this domain	WPPSI-III	Wechsler Preschool and Primary Scale of Intelligence-3rd Edition Geometric Design
Nonverbal reasoning	No subdomains for this domain	WPPSI-III	Wechsler Preschool and Primary Scale of Intelligence-3rd Edition Mazes
Nonverbal reasoning	No subdomains for this domain	WPPSI-III	Wechsler Preschool and Primary Scale of Intelligence-3rd Edition Picture Completion
Sensory	No subdomains for this domain	ITSP	Infant/toddler Sensory Profile Auditory Processing
Sensory	No subdomains for this domain	ITSP	Infant/toddler Sensory Profile Low Threshold
Sensory	No subdomains for this domain	ITSP	Infant/toddler Sensory Profile Oral Processing
Sensory	No subdomains for this domain	ITSP	Infant/toddler Sensory Profile Low Registration
Sensory	No subdomains for this domain	ITSP	Infant/toddler Sensory Profile Sensation Seeking
Sensory	No subdomains for this domain	ITSP	Infant/toddler Sensory Profile Sensory Sensitivity
Sensory	No subdomains for this domain	ITSP	Infant/toddler Sensory Profile Sensation Avoiding
Sensory	No subdomains for this domain	ITSP	Infant/toddler Sensory Profile Tactile Processing
Sensory	No subdomains for this domain	ITSP	Infant/toddler Sensory Profile Vestibular Processing
Sensory	No subdomains for this domain	ITSP	Infant/toddler Sensory Profile Visual Processing
Social-cognition	Overall	CHQ50	The Child Health Questionnaire 50 Mental Health
Social-cognition	Overall	CHQ50	The Child Health Questionnaire 50 Self-esteem
Social-cognition	Overall	CHQ50	The Child Health Questionnaire 50 Social-Emotional Role
Social-cognition	Overall	CHQ50	The Child Health Questionnaire 50 Social-Physical Role
Social-cognition	No subdomains for this domain	AvEDI	Early Development Instrument (Australia): Emotional Health and Maturity
Social-cognition	No subdomains for this domain	AvEDI	Early Development Instrument (Australia): Social Knowledge and Competence
Social-cognition	No subdomains for this domain	BSID-III	Bayley Scales of Infant Development-3rd edition Social-Emotional
Social-cognition	No subdomains for this domain	BSID-III	Bayley Scales of Infant Development-3rd edition Social
Social-cognition	No subdomains for this domain	EDI	Early Development Instrument Emotional Health and Maturity

© 2022 Reighard C et al. *JAMA Network Open.*

Social-cognition	No subdomains for this domain	EDI	Early Development Instrument Social Knowledge and Competence
Social-cognition	No subdomains for this domain	NEPSY-II	Developmental Neuropsychological Assessment Battery-2nd Edition Affect Recognition
Social-cognition	No subdomains for this domain	NEPSY-II	Developmental Neuropsychological Assessment Battery-2nd Edition Theory of Mind
Social-cognition	No subdomains for this domain	Parent Questionnaire	Parent Social-Emotional Questionnaire
Social-cognition		Social-Emotional	Social Emotional Scale
Unable to be mapped to a domain	Unable to be mapped to a subdomain	CHQ50	The Child Health Questionnaire 50 Family Activities
Unable to be mapped to a domain	Unable to be mapped to a subdomain	CHQ50	The Child Health Questionnaire 50 Family Cohesion
Unable to be mapped to a domain	Unable to be mapped to a subdomain	CHQ50	The Child Health Questionnaire 50 Parental Emotional Impact
Unable to be mapped to a domain	Unable to be mapped to a subdomain	Executive Function (unspecified)	20 Questions
Unable to be mapped to a domain	Unable to be mapped to a subdomain	GDS	Gesell Developmental Schedule Response to Objects
Unable to be mapped to a domain	Unable to be mapped to a subdomain	GDS	Gesell Developmental Schedule Response to People
Unable to be mapped to a domain	Unable to be mapped to a subdomain	SB-V	Stanford-Binet V Fluid Reasoning
Unable to be mapped to a domain	Unable to be mapped to a subdomain	SB-V	Stanford-Binet V Knowledge
Unable to be mapped to a domain	Unable to be mapped to a subdomain	SB-V	Stanford-Binet V Quantitative Reasoning
Unable to be mapped to a domain	Unable to be mapped to a subdomain	SB-V	Stanford-Binet V Visual-spatial Processing
Unable to be mapped to a domain	Unable to be mapped to a subdomain	WASI	Wechsler Abbreviated Scale of Intelligence Matrix Vocab
Unable to be mapped to a domain	Unable to be mapped to a subdomain	WPPSI-IV (CR)	Wechsler Preschool and Primary Scale of Intelligence, fourth edition, Chinese version Visual-Spatial Index
Unable to be mapped to a domain	Unable to be mapped to a subdomain	WPPSI-IV (CR)	Wechsler Preschool and Primary Scale of Intelligence, fourth edition, Chinese version Fluid Reasoning Index

© 2022 Reighard C et al. *JAMA Network Open.*

eTable 4. Outcomes From Each of the 108 Reviewed Studies

Study	List of outcomes
Ludman et al,[23] 1990	GMDS development quotient, GMDS eye/hand, GMDS hearing and speech, GMDS mental age, GMDS motor, GMDS performance, GMDS personal/social
Ludman et al,[24] 1993	BPVS, GMDS development quotient, GMDS eye/hand, GMDS performance, RDLS comprehension, RDLS expressive language, Wallin B pegboard
The Victorian Infant Collaboration[25] 1996	Blindness, Cerebral palsy diagnosis, Hearing impairment, WISC-III FSIQ, WPPSI-R IQ
Kayaalp et al,[26] 2006	CBCL aggressive behavior, CBCL anxious/depressed, CBCL attention problems, CBCL delinquent behavior, CBCL externalizing, CBCL internalizing, CBCL social problems, CBCL somatic complaints, CBCL thought problems, CBCL total problems, CBCL withdrawn, CDI
Bartels et al,[27] 2009	CTRS-R:S, National standardized test score
DiMaggio et al,[28] 2009	Psychiatric diagnoses
Kalkman et al,[29] 2009	CBCL externalizing, CBCL internalizing, CBCL total problems, Psychiatric interview
Majnemer et al,[30] 2009	CBCL total problems, CHQ, PDMS, PPVT, VABS-I daily living, VABS-I socialization, WeeFIM, WPPSI-R IQ,
Wilder et al,[31] 2009	Learning disability diagnosis
Fan et al,[32] 2010	GDS language, GDS movement, GDS response to objects, GDS response to people
Walker et al,[33] 2010	BSID-III cognition, BSID-III expressive language, BSID-III fine motor, BSID-III gross motor, BSID-III receptive language, Parent social-emotional questionnaire
DiMaggio et al,[34] 2011	Psychiatric diagnoses
Flick et al,[35] 2011	CAT math, CAT phonics, CAT spelling, CAT written language, IEP Emotion/Behavior, IEP Speech/Language, Learning disability diagnosis, TCS memory, TCS total cognitive
Hansen et al,[36] 2011	National standardized test scores, Teacher rating, Test non-attainment
Andropoulos et al,[37] 2012	ABS conceptual, ABS general adaptive, ABS practical, ABS social, BSID-III cognition, BSID-III language, BSID-III motor, Social-emotional scale
Block et al,[38] 2012	IOWA standardized test, Very poor academic performance overall
Filan et al,[39] 2012	BSID-II MDI, BSID-II PDI
Ing et al,[40] 2012	CBCL externalizing, CBCL internalizing, CBCL total problems, CELF-expressive, CELF-receptive, CELF-total, CPM, MAND, PPVT, SDMT oral, SDMT written
Long et al a,[41] 2012	AIMS
Long et al b,[42] 2012	BSID-III cognition, BSID-III expressive language, BSID-III fine motor, BSID-III gross motor, BSID-III language composite, BSID-III motor composite, BSID-III receptive language
Rocha et al,[43] 2012	GMDS eye/hand, GMDS hearing and speech, GMDS performance, GMFCS, Hearing impairment
Sananes et al,[44] 2012	ASQ communication, ASQ fine motor, ASQ gross motor, ASQ personal/social, ASQ problem solving, BSID-II MDI, PDMS
Sprung et al,[45] 2012	ADHD Diagnosis
Walker et al,[46] 2012	BSID-III cognition, BSID-III expressive language, BSID-III fine motor, BSID-III gross motor, BSID-III receptive language
Yang et al,[47] 2012	K-ABC location memory, K-ABC picture, K-ABC triangle, K-ABC visual analogy
Bong et al,[48] 2013	Learning disability diagnosis, PSLE

© 2022 Reighard C et al. *JAMA Network Open.*

Fan et al,[49] 2013	WPPSI-III animal house, WPPSI-III arithmetic, WPPSI-III block design, WPPSI-III comprehension, WPPSI-III FSIQ, WPPSI-III geometric design, WPPSI-III information, WPPSI-III mazes, WPPSI-III picture completion, WPPSI-III PIQ, WPPSI-III similarities, WPPSI-III vocabulary, WPPSI-III VIQ
Hansen et al,[50] 2013	National standardized test, teacher rating, test score non-attainment
Minutillo et al,[51] 2013	Blindness, Hearing impairment, Cerebral palsy diagnosis, GMDS development quotient
Andropoulos et al,[52] 2014	BSID-III cognitive, BSID-III language, BSID-III motor
Cheng et al,[53] 2014	BSID-II PDI, BSID-II MDI
Garcia Guerra et al,[54] 2014	ABAS-II general adaptive composite, Beery VMI, WPPSI-III FSIQ, WPPSI-III PIQ, WPPSI-III VIQ
Gaynor et al,[55] 2014	ADHD rating scale IV impulsivity, ADHD rating scale IV inattention, Beery VMI, CBCL pervasive developmental problem scale, WPPSI-III FSIQ, WPPSI-III PIQ, WPPSI-III processing speed, WPPSI-III VIQ, Woodcock-Johnson III mathematics, Woodcock-Johnson III reading
Ing et al a,[56] 2014	CELF-expressive CELF-receptive, CELF-total, CPM, ICD-9 CM mental disorder diagnosis, WAMSE numeracy, WAMSE reading, WAMSE spelling, WAMSE writing
Ing et al b,[57] 2014	CBCL externalizing, CBCL internalizing, CBCL total problems, CELF-expressive, CELF-receptive, CELF-total, CPM, MAND, PPVT, SDMT oral, SDMT written
Ko et al,[58] 2014	ADHD Diagnosis
Morriss et al,[59] 2014	Blindness, BSID-III cognition, BSID-II MDI, BSID-II PDI, GMFCS, Hearing impairment
Stratmann et al,[60] 2014	CBCL total problems, Recognition memory color familiarity, Recognition memory color recognition, Recognition memory spatial familiarity, Recognition memory spatial recollection, WASI FSIQ, WASI PIQ, WASI VIQ
Williams et al,[61] 2014	IEP need, Vermont SAT math, Vermont SAT reading, Very poor academic performance math, Very poor academic performance reading
Yin et al,[62] 2014	WMS-CR accumulation, WMS-CR backward counting, WMS-CR comprehension memory, WMS-CR digit span, WMS-CR figure recognition, WMS-CR forward counting, WMS-CR image recalling, WMS-CR verbal-paired associates, WMS-CR visual reproduction
Backeljauw et al,[63] 2015	OWLS, WISC-III FSIQ, WISC-III PIQ, WISC-III VIQ
Bakri et al,[64] 2015	CBCL aggressive behavior, CBCL anxious/depressed, CBCL emotionally reactive, CBCL externalizing, CBCL internalizing, CBCL sleep problems, CBCL somatic complaints, CBCL withdrawn, DSM-IV 3-point scale
Gano et al,[65] 2015	WPPSI-III FSIQ, WPPSI-III PIQ, WPPSI-III VIQ
Hansen et al,[66] 2015	National standardized test, Teacher evaluations, Test-score non-attainment
Ko et al,[67] 2015	Diagnosis of ASD
Naguib et al,[68] 2015	ABAS-II conceptual, ABAS-II general adaptive composite, ABAS-II motor, ABAS-II practical, ABAS-II social, CBCL internalizing, CBCL externalizing, CBCL total problems, SB-V fluid reasoning, SB-V FSIQ, SB-V knowledge, SB-V nonverbal IQ, SB-V quantitative reasoning, SB-V VIQ, SB-V visual-spatial processing, SB-V working memory
Petrácková et al,[69] 2015	CHQ 50 bodily pain, CHQ 50 change of health, CHQ 50 family activities, CHQ 50 family cohesion, CHQ 50 general behavior, CHQ 50 general health, CHQ 50 mental health, CHQ 50 parental emotional impact, CHQ 50 parental time impact, CHQ 50 physical functioning, CHQ 50 self-esteem, CHQ 50 social-emotional role, CHQ 50 social-physical role, SON-R
Taghon et al,[70] 2015	Go/No go task
Aun et al,[71] 2016	G-TVPS, HK-WISC general comprehension, HKLL, Woodcock-Johnson Visual Matching Test

© 2022 Reighard C et al. *JAMA Network Open.*

Davidson et al,[72] 2016	BSID-III adaptive behavior, BSID-III cognition, BSID-III communication, BSID-III community use, BSID-III expressive language, BSID-III fine motor, BSID-III functional pre-academics, BSID-III gross motor, BSID-III health and safety, BSID-III home living, BSID-III language composite, BSID-III leisure, BSID-III motor composite, BSID-III receptive language, BSID-III self-care, BSID-III self-direction, BSID-III social, BSID-III social-emotional, McArthur–Bates Communicative Development Inventory
Diaz et al,[73] 2016	WPPSI-III FSIQ, WPPSI-III PIQ, WPPSI-III processing speed, WPPSI-III VIQ
Djurhuus et al,[74] 2016	School performance/grades, Test score non-attainment
Doberschuetz et al,[75] 2016	BSID-II PDI, BSID MDI
Graham et al,[76] 2016	EDI communication skills/general knowledge, EDI emotional maturity, EDI language and cognitive development, EDI physical well-being, EDI social competence, EDI total score
Hansen et al,[77] 2016	GMDS hearing and speech, HAWIVA-III FSIQ, HAWIVA-III nonverbal IQ, HAWIVA-III processing speed, HAWIVA-III VIQ, KET-KID articulation, KET-KID global score, KET-KID nonverbal development, KET-KID psychomotor development, KET-KID verbal comprehension, KET-KID verbal development, KET-KID verbal memory, KET-KID visual memory, KET-KID visual perception, KET-KID visual construction
Hoffman et al,[78] 2016	BSID-III cognition, BSID-III language composite, BSID-III motor composite
O'Leary et al,[79] 2016	EDI communication skills/general knowledge, EDI early developmental vulnerability, EDI emotional maturity, EDI language and cognitive development, EDI multiple challenge index, EDI physical well-being, EDI social competence
Poor Zamany Nejat Kermany et al,[80] 2016	Backward digit span, Forward digit span, Phonetic verbal fluency, Semantic verbal fluency
Seltzer et al,[81] 2016	VABS-II communication standard, VABS-II composite, VABS-II daily living, VABS-II motor skills, VABS-II socialization, VABS-II total sum
Sun et al,[82] 2016	ABAS-II conceptual, ABAS-II general adaptive composite, ABAS-II practical, ABAS-II social, BRIEF GEC, CBCL internalizing, CBCL externalizing, CBCL total problems, CPT2 number commissions, CPT2 number omission, CVLTC total trials 1-5, DKEFS TMT conditions 1, 2, 3, 4, & 5, Grooved pegboard dominant hand, Grooved pegboard nondominant hand, NEPSY-II comprehension of instructions, NEPSY-II delayed memory for faces, NEPSY-II memory for faces, NEPSY-II speeded naming, NEPSY-II word generation, WASI block design, WASI FSIQ, WASI matrix reasoning, WASI PIQ, WASI similarities, WASI VIQ, WASI vocabulary, WISC-IV coding, WISC-IV digit span
Aly et al,[83] 2017	BSID II MDI, BSID-II PDI
Birajdar et al,[84] 2017	Blindness, Hearing impairment, GMDS development quotient, GMFCS
Clausen et al,[85] 2017	National standardized exam, Test score non-attainment
Conrad et al,[86] 2017	WAIS-III VIQ, WISC-III VIQ
de Heer et al,[87] 2017	SON-R
Glatz et al,[88] 2017	Conscription IQ test, school performance/grades
Harmsen et al,[89] 2017	Bourdon-Vos test sustained attention, M-ABC total score, RAKIT, WISC-III NL FSIQ, WISC-III NL PIQ, WISC-III NL VIQ
Hu et al,[90] 2017	ADHD diagnosis, IEP Emotional/Behavior, IEP Speech/Language, Learning disability diagnosis, OLSAT language, OLSAT mathematics, OLSAT reading, OLSAT spelling, OLSAT total battery
Ing et al a,[91] 2017	ADHD diagnosis, Developmental delay diagnosis, ICD-9 mental disorder diagnosis
Ing et al b,[92] 2017	CELF-expressive, CELF-receptive, CELF-total, CPM
Ing et al c,[93] 2017	CBCL internalizing, CBCL externalizing, CBCL total problems, CELF-expressive, CELF-receptive, CELF-total, CPM, MAND, SDMT oral , SDMT written

© 2022 Reighard C et al. *JAMA Network Open.*

Lap et al,[94] 2017	AVLT verbal learning, AVLT verbal long-term memory, AVLT verbal recognition memory, BRIEF GEC, CBCL total problems, M-ABC balance skills, M-ABC ball skills, M-ABC manual dexterity, M-ABC total score, NEPSY-II design copy, TEA-Ch NL response inhibition, TEA-Ch NL sustained auditory attention, TEA-Ch NL visual attention, WISC-III-NL FSIQ, WISC-III-NL PIQ, WISC-III-NL VIQ
Nestor et al,[95] 2017	Developmental delay, Neurological diagnoses, Psychiatric diagnoses
Terushkin et al,[96] 2017	Psychiatric diagnoses, School performance/grades
Zhang et al,[97] 2017	RSPM IQ
Berghmans et al,[98] 2018	ITSP low threshold (quadrant 3+4), ITSP oral sensory processing, ITSP quadrant 1 low registration, ITSP quadrant 2 sensation seeking, ITSP quadrant 3 sensory sensitivity, ITSP quadrant 4 sensation, ITSP sensory auditory processing, ITSP tactile processing, ITSP vestibular processing, ITSP visual processing
Castellheim et al,[99] 2018	A-TAC ADHD, A-TAC ASD, A-TAC learning disabilities
Hunt et al,[100] 2018	Blindness, Cerebral palsy diagnosis, DAS-II, GMFCS, Hearing impairment, WISC-III FSIQ, WISC-IV IQ
Kozanhan et al,[101] 2018	CPRI
Lv et al,[102] 2018	BSID-II MDI, BSID-II PDI
Schneuer et al,[103] 2018	AvEDI communication skills and general knowledge, AvEDI emotional health and maturity, AvEDI language and cognitive development, AvEDI physical health and well-being, AvEDI social knowledge and competence, AvEDI developmentally high risk, NAPLAN numeracy, NAPLAN reading
Tsai et al,[104] 2018	ICD-9-CM ADHD diagnosis
Warner et al,[105] 2018	Beery motor coordination, Beery visual perception, Beery VMI, Boston Naming Test, BRIEF GEC, CBCL ADHD problems, CBCL externalizing problems, CBCL Internalizing, CBCL total problems, CLDQ math scale, CLDQ reading scale, CPT2 detectability, CPT2 hit reaction time, CPT2 number of commissions, CPT2 number of omission, CPT2 variability, CTOPP rapid naming composite score, D-KEFS trail making test (conditions 1-5), D-KEFS tower test, D-KEFS verbal fluency: categorical fluency, Expressive language composite, Fine motor composite, Grooved pegboard dominant hand, Grooved pegboard fine motor, Grooved pegboard nondominant hand, WASI FSIQ, WASI matrix reasoning, WASI matrix vocab, WASI vocabulary, Wisconsin Card Sort: preservative responses, Wisconsin Card Sort: preservative errors, WRAML-2: attention/concentration index, WRAML-2 delayed verbal recall composite, WRAML-2 design memory, WRAML-2 design recognition, WRAML-2 story memory delay recall, WRAML-2 story memory recognition, WRAML-2 verbal learning delay recall, WRAML-2 verbal learning recognition, WRAML-2 verbal memory index, WRAML-2 verbal recognition composite
Banerjee et al,[106] 2019	Attention spatial forward, BDS, CPT2 number commissions, CPT2 number omission, CPT2 preservations, Educational attainment, Executive function 20 questions, Executive function color word inhibition, Executive function color word inhibition switching, Executive function number-letter sequencing, FDS, Processing speed color naming, Processing speed digit symbol, Processing speed dominant hand speed, Processing speed letter sequencing, Processing speed non-dominant hand speed, Processing speed number sequencing, Processing speed verbal fluency, RCFT, WASI block design
Khochfe et al,[107] 2019	ECBI

© 2022 Reighard C et al. *JAMA Network Open.*

McCann et al,[108] 2019	ABAS-II general adaptive composite, ADHD diagnosis, ASD diagnosis, Blindness, BRIEF-P GEC, CBCL externalizing, CBCL internalizing, CBCL total problems, Cerebral palsy diagnosis, CMS numbers, CMS word lists I, CMS word lists II, Developmental delay, Hearing impairment, NEPSY-II affect recognition, NEPSY-II auditory attention, NEPSY-II design copy, NEPSY-II fingertip tapping repetitions, NEPSY-II fingertip tapping sequences, NEPSY-II inhibition, NEPSY-II memory for names, NEPSY-II sentence repetition, NEPSY-II speed naming, NEPSY-II statue, NEPSY-II theory of mind, NEPSY-II word generation, WIAT-II numerical composite, WIAT-II word reading, WIAT-II spelling, , WPPSI-III FSIQ, WPPSI-III performance IQ, WPPSI-III processing speed, WPPSI-III verbal IQ
O'Leary et al,[109] 2019	EDI communication skills and general knowledge, EDI early developmental vulnerability, EDI emotional healthy and maturity, EDI language and cognitive development, EDI multiple challenge index, EDI physical health and well-being, EDI social knowledge and competence
Sun et al,[110] 2019	BSID-II MDI, BSID-II PDI
Vedovelli et al,[111] 2019	Beery VMI, CPM, VABS-I deficiency in subscale, WISC-IV coding
Warner et al,[112] 2019	OTB conditioned position response, OTB delayed match to sample, OTB incremental repeated acquisition, OTB progressive ratio, OTB temporal response differentiation
Zaccariello et al,[113] 2019	Beery motor coordination, Beery visual perception, Beery VMI, BRIEF GEC, CBCL ADHD problems, CBCL externalizing, CBCL internalizing, CBCL total problems, CLDQ math scale, CLDQ reading scale, CPT2 detectability, CPT2 hit reaction time, CPT2 number of commissions, CPT2 number of omission, CPT2 variability, CTOPP rapid naming composite score, D-KEFS trail making test (conditions 1-5), D-KEFS tower test, D-KEFS verbal fluency: categorical fluency, Grooved pegboard dominant hand, Grooved pegboard nondominant hand, WASI FSIQ, WASI matrix reasoning, WASI vocabulary, Wisconsin Card Sort: preservative responses, Wisconsin Card Sort: preservative errors, WRAML-2: attention/concentration index, WRAML-2 delayed verbal recall composite, WRAML-2 design memory, WRAML-2 design recognition, WRAML-2 story memory delay recall, WRAML-2 story memory recognition, WRAML-2 verbal learning delay recall, WRAML-2 verbal learning recognition, WRAML-2 verbal memory index, WRAML-2 verbal recognition composite
Han et al,[114] 2020	Ambulation, Blindness, Cerebral palsy diagnosis, Educational attainment, Hearing impairment
Batta et al,[115] 2020	GMDS-II GQ deficiency, cerebral palsy, blindness, sensorineural deafness
Feng et al,[116] 2020	Developmental delay diagnosis
Hakanson et al,[117] 2020	ADHD diagnosis, educational attainment
Ing et al,[118] 2020	ADHD medication use
Jacola et al,[119] 2020	Woodcock-Johnson III Test of Academic Achievement Broad Reading, Woodcock-Johnson III test of Cognitive Abilities: General Intellectual Ability/IQ, Broad Attention, Processing Speed, Working Memory
Kobayashi et al,[120] 2020	Japanese Ages and Stages Questionnaire, Third Edition (J-ASQ-3): Communication, Gross motor, Fine motor, Problem solving, Personal-social
Sedighnejad et al,[121] 2020	ADHD diagnosis
Walkden et al,[122] 2020	Key stage 2 English, Key stage 2 Mathematics, Key stage 2 Science, Key stage 3 English, Key stage 3 Mathematics, Key stage 3 Science, Key stage 4 total points, Key Stage 4 exam entries, Key Stage 2 non-attainment, Key Stage 3 non-attainment, Key stage 4 English A*, A, b, or C grade, Key stage 4 mathematics A*, A, b, or C grade, Key stage 4 science 2 “good” passes (C grade or above), Wechsler Intelligence Scale for Children global intelligence quotient, Wechsler Intelligence Scale for Children verbal intelligence quotient, Test of Everyday Attention for Children: Sky search task (selective attention), Test of Everyday Attention for Children: Opposite worlds task (attentional control/switching), Counting span task (working memory), Wechsler Abbreviated Scale of Intelligence global intelligence quotient, M-ABC Heel-to-toe walking task , M-ABC Preferred hand peg placing, M-ABC Nonpreferred hand peg placing, M-ABC Bean bag

© 2022 Reighard C et al. *JAMA Network Open.*

	throwing task, Strengths and Difficulties Questionnaire, Skuse sociocognitive dysfunction score, Child's Communication Checklist, Wechsler Objective Reading Dimensions Basic reading test , Spelling test, Phoneme deletion task, Wechsler Objective Language Dimensions comprehension task, Wechsler Objective Language Dimensions verbal expression task, Real-word reading test, Non–real-word reading test, Test of Word Reading Efficiency word-reading test, Test of Word Reading Efficiency non-word–reading test
Gleich et al,[123] 2021	ADHD diagnosis, LD diagnosis
Ing et al,[124] 2021	CPM, MAND, PPVT, SDMT written, SDMT oral, CELF-Expressive, CELF-Receptive, CELF-Total, CBCL Internalizing, CBCL externalizing, CBCL Total problems
Lauritzen et al,[125] 2021	Education attainment, dyslexia, dyscalculia, special needs teaching, educational psychologist counseling, Wechsler Adult Intelligence Scale Version IV (WAIS-IV): FSIQ, verbal comprehension, perceptual reasoning, working memory, processing speed
Partanen et al,[126] 2021	Woodcock-Johnson III test of Cognitive Abilities: General Intellectual Ability/IQ, Broad Attention, and Processing Speed
van Hoorn et al,[127] 2021	M-ABC (Total Impairment Score)
Walsh et al,[128] 2021	BSID-III cognitive, BSID-III language, BSID-III motor
Warner et al,[129] 2021	ADHD diagnosis, LD diagnosis, LD reading, LD math, LD writing, IEP speech/language, IEP emotion/behavior
Zhou et al,[130] 2021	cerebral palsy diagnosis, developmental delay, hearing or vision impairment, language/behavioral/psychomotor disorder, intervention for "neurodevelopmental problem", WPPSI-IV-CR (Chinese version): FSIQ, Verbal Comprehension Index (VCI), Visual-Spatial Index (VSI), Fluid Reasoning Index (FRI), Working Memory Index (WMI), and Processing Speed Index (PSI)
Abbreviations: ABAS-II: Adaptive Behavior Assessment System-2nd Edition, ABS: Adaptive Behavior Scale, ADHD: Attention-deficit/hyperactivity Disorder, AIMS: Alberta Infant Motor Scale, ASD: Autism Spectrum Disorder, ASQ: Ages & Stages Questionnaire, A-TAC: The Autism-Tics, ADHD, and other Comorbidities Inventory, AvEDI: Early Development Instrument (Australia), AVLT: Rey Auditory Verbal Learning Test, BDS: Backward Digit Span Test, BPVS: British Picture Vocabulary Scale, BRIEF-GEC: Behavior Rating Inventory of the Executive Functions Global Executive Composite, BRIEF-P GEC: Behavior Rating Inventory of Executive Function, Preschool version Global Executive Composite, BSID-II Bayley Scales of Infant Development-2nd Edition, BSID-III: Bayley Scales of Infant Development-3rd Edition, CAT: California Achievement Test, CBCL: Child Behavior Checklist, CDI: Children's Depression Inventory, CELF: Clinical Evaluation of Language Fundamentals, CHQ: Child Health Questionnaire, CLDQ: Colorado Learning Difficulties Questionnaire, CMS: Children's Memory Scale, CPM: Raven's Colored Progressive Matrices, CPRI: Child Post-Traumatic Stress Disorder Reaction Index, CPT2: Conner's Continuous Performance Test II, CTOPP: Comprehensive Test of Phonological Processing, CTRS-R:S: Conner's' Teacher Rating Scale--Revised: Short Form, CVLT-C: California Verbal Learning Test--Children, DAS-II: Differential Abilities Scale Second Edition, DKEFS: Delis-Kaplan Executive Function System, DSM IV: Diagnostic and Statistical Manual of Mental Disorders--4th Edition, ECBI: Eyberg Child Behavior Inventory, EDI: Early Development Instrument, FDS: Forward Digit Span Test, FSIQ: Full Scale Intelligence Quotient, GDS: Gesell Developmental Schedule, GMDS: Griffiths Mental Development Scale, GMFCS: Gross Motor Function Classification System, GQ: General Development Quotient, G-TVPS: Gardner Test of Visual-Perceptual Skills Revised, HAWIVA-III: Hannover--Wechsler Intelligence Scale, 3rd Edition, HKLL: Hong Kong List Learning, HK-WISC: Hong Kong-Wechsler Intelligence Scale for Children, ICD-9: International Classification of Disease--9th Edition, ICD-9 CM: International Classification of Disease--9th Edition, Clinical Modification, IEP: Individualized Education Plan, IQ: Intelligence Quotient, ITSP: Infant/toddler Sensory Profile, K-ABC: Kaufmann Assessment Battery for Children, KET-KID: Kognitiver Enwicklungstest fur das Kindergartenalter, M-ABC: Movement Assessment Battery for Children, MAND: McCarren Assessment of Neuromuscular Development, MDI: Mental Development Index, NAPLAN: National Assessment Program-Literacy and Numeracy, NEPSY-II: Developmental Neuropsychological Assessment Battery-2nd Edition, OLSAT: Stanford/Otis-Lennon School Ability Test, OTB: Operant Test Battery, OWLS: Oral and Written Language Scales, PDI: Psychomotor Development Index, PDMS: Peabody Developmental Motor Scales, PPVT: Peabody Picture Vocabulary Test, PIQ: Performance Intelligence Quotient, PSLE: Primary School Leaving Examination, RAKIT: Revised Amsterdam Intelligence Test, RCFT: Rey Complex Figure Test and Recognition Trial, RDLS: Reynell Developmental Language Scales, RSPM: Raven's Standard Progressive Matrices, SB-V: Stanford-Binet V, SDMT: Symbol Digit Modality Test, SON-R: Hogrefe/Snijders-Oomen Non-Verbal Intelligence Test--Revised, TCS: Test of Cognitive Skills, TEA-Ch NL: Test of Everyday Attention for Children, Dutch Version, TMT: Trial Making Test, VABS-I: Vineland Adaptive Behavior Scales, 1st Edition, VABS-II: Vineland Adaptive Behavior Scales, 2nd Edition, VIQ: Verbal Intelligence Quotient, VMI: Visual-motor Integration, WAIS-III: Wechsler Adult Intelligence Scale 3rd Edition, WAMSE: Western Australian Literacy and Numeracy	

© 2022 Reighard C et al. *JAMA Network Open.*

Standardized Test, WASI: Wechsler Abbreviated Scale of Intelligence, WIAT-II: Wechsler Individual Achievement Test-2nd Edition, WISC-III: Wechsler Intelligence Scale for Children-3rd Edition, WISC-III-NL: Wechsler Intelligence Scale for Children-3rd Edition, Dutch Version, WISC-IV: Wechsler Intelligence Scale for Children-4th Edition, WMS-CR: Wechsler Memory Scale--Chinese Revision, WPPSI-III: Wechsler Preschool and Primary Scale of Intelligence-3rd Edition, WPPSI-IV-CR: Wechsler Preschool and Primary Scale of Intelligence-Fourth Edition Chinese Version, WPPSI-R: Wechsler Preschool and Primary Scale of Intelligence-Revised, WRAML-2: Wide Range Assessment of Memory and Learning, 2nd Edition

© 2022 Reighard C et al. *JAMA Network Open.*

eTable 5. Characteristics of All 108 Reviewed Studies

Study	Study Design	Unexposed controls	Number of exposures	Type of Procedure	Major comorbidities	Age at exposure
Ludman et al,[23] 1990	PO	Yes	NS	Emergency neonatal surgery	Emergency neonatal surgery	<30 days
Ludman et al,[24] 1993	PO	Yes	Comb	Emergency neonatal surgery	Emergency neonatal surgery	<30 days
The Victorian Infant Collaboration[25] 1996	PO	Yes	Comb	Various specified	Extreme prematurity	Birth hospitalization
Kayaalp et al,[26] 2006	Ret	No	S/M	Esophageal dilation	Caustic ingestion	4-18 years
Bartels et al,[27] 2009	PO	Yes	NS	Various unspecified	No	Variable
DiMaggio et al,[28] 2009	Ret	Yes	SPM	Inguinal hernia repair	No	12-48 months
Kalkman et al,[29] 2009	Ret	No	Comb	Urologic surgery	No	Variable
Majnemer et al,[30] 2009	PO	No	SPM	Heart surgery	Congenital heart disease	<6 months
Wilder et al,[31] 2009	Ret	Yes	S/M	Various specified	No	<4 years
Fan et al,[32] 2010	PO	No	SPM	Heart surgery	Congenital heart disease	<4 years
Walker et al,[33] 2010	PO	Yes	SPM	Pyloromyotomy	No	10-77 days
DiMaggio et al,[34] 2011	Ret	Yes	Comb	Various specified	No	<3 years
Flick et al,[35] 2011	Ret	Yes	S/M	Various unspecified	No	<2 years
Hansen et al,[36] 2011	Ret	Yes	SPM	Inguinal hernia repair	No	<1 year
Andropoulos et al,[37] 2012	PO	No	SPM	Heart surgery	Congenital heart disease	<1 month
Block et al,[38] 2012	Ret	No	SPM	Various specified	No	<1 year
Filan et al,[39] 2012	Ret	Yes	Comb	Various specified	Extreme prematurity	26-41 weeks PMA
Ing et al,[40] 2012	Ret	Yes	S/M/Comb	Various specified	No	<3 years
Long et al a,[41] 2012	PO	No	SPM	Heart surgery	Congenital heart disease	<8 weeks
Long et al b,[42] 2012	PO	No	NS	Heart surgery	Congenital heart disease	<8 weeks
Rocha et al,[43] 2012	PO	No	NS	Congenital diaphragmatic hernia repair	Congenital diaphragmatic hernia	<1 month
Sananes et al,[44] 2012	PO	No	SPM	Heart surgery	Congenital heart disease	<3 months
Sprung et al,[45] 2012	Ret	Yes	S/M	Various unspecified	No	<2 years
Walker et al,[46] 2012	PO	Yes	NS	Cardiac and major non-cardiac	Congenital heart disease and other	<3 months
Yang et al,[47] 2012	PO	No	SPM	Strabismus surgery	No	5-10 years
Bong et al,[48] 2013	Ret	Yes	SPM	Minor surgery	No	<1 year

© 2022 Reighard C et al. *JAMA Network Open.*

Fan et al,[49] 2013	PO	No	SPM	Strabismus surgery	No	<4 years
Hansen et al,[50] 2013	Ret	Yes	SPM	Pyloromyotomy	No	<3 months
Minutillo et al,[51] 2013	Ret	No	Comb	Gastroschisis repair	Gastroschisis	<1 month
Andropoulos et al,[52] 2014	Ret	No	Comb	Heart surgery	Congenital heart disease	<30 days
Cheng et al,[53] 2014	PO	No	SPM	Heart surgery	Congenital heart disease	<3 years
Garcia Guerra et al,[54] 2014	PO	No	SPM	Heart surgery	Congenital heart disease	<6 weeks
Gaynor et al,[55] 2014	Ret	No	SPM	Heart surgery	Congenital heart disease	<6 months
Ing et al a,[56] 2014	Ret	Yes	Comb	Various specified	No	<3 years
Ing et al b,[57] 2014	Ret	Yes	Comb	Various specified	No	<10 years
Ko et al,[58] 2014	Ret	Yes	S/M/Comb	Various unspecified	No	<3 years
Morriss et al,[59] 2014	Ret	Yes	Comb	Various specified	Extreme prematurity	Variable
Stratmann et al,[60] 2014	Amb	Yes	Comb	Various specified	No	<1 year
Williams et al,[61] 2014	Ret	Yes	S	Various specified	No	<1 year
Yin et al,[62] 2014	PO	No	SPM	Inguinal hernia repair	No	7-13 years
Backeljauw et al,[63] 2015	Ret	Yes	Comb	Various unspecified	No	1 day to 3.8 years
Bakri et al,[64] 2015	Ret	Yes	M	Various unspecified	No	1.5-5 years
Gano et al,[65] 2015	PO	No	S/M	Various specified	Prematurity	<42 weeks PMA
Hansen et al,[66] 2015	Ret	Yes	SPM	Neurosurgery	Neurosurgical condition	Not specified
Ko et al,[67] 2015	Ret	Yes	S/M/Comb	Various unspecified	No	<2 years
Naguib et al,[68] 2015	PO	Yes	SPM	Heart surgery	Congenital heart disease	<1 year
Petrácková et al,[69] 2015	PO	No	S	Cleft lip repair	No	8 days
Taghon et al,[70] 2015	Amb	Yes	Comb	Various specified	No	0-2 years
Aun et al,[71] 2016	PO	Yes	S	Various specified	No	5-12 years
Davidson et al,[72] 2016	RCT	Yes	SPM	Inguinal hernia repair	No	<60 weeks PMA
Diaz et al,[73] 2016	Ret	No	Comb	Heart surgery	Congenital heart disease	<12 months
Djurhuus et al,[74] 2016	Ret	Yes	S/M	Cholesteatoma surgery	No	Mean age 9.1 years
Doberschuetz et al,[75] 2016	Amb	Yes	Comb	Gastrointestinal malformation repair	Gastrointestinal malformation	<7 days
Graham et al,[76] 2016	Ret	Yes	S/M	Various specified	No	<4 years
Hansen et al,[77] 2016	Amb	No	M	Heart surgery	Congenital heart disease	<5 years
Hoffman et al,[78] 2016	Ret	No	Comb	cardiac procedures	Congenital heart disease	Variable
O'Leary et al,[79] 2016	Ret	Yes	Comb	Various unspecified	No	Before age 5.7 years
Poor Zamany Nejat Kermany et al,[80] 2016	Amb	Yes	S/M	Glaucoma surgery	No	<3 years
Seltzer et al,[81] 2016	Amb	No	SPM	Heart surgery	Congenital heart disease	<1 month

© 2022 Reighard C et al. *JAMA Network Open.*

Sun et al,[82] 2016	Amb	Yes	SPM	Inguinal hernia repair	No	<3 years
Aly et al,[83] 2017	PO	No	SPM	Heart surgery	Congenital heart disease	<1 month
Birajdar et al,[84] 2017	Ret	No	SPM	Laparotomy for malrotation	No	<5 days (mean)
Clausen et al,[85] 2017	Ret	Yes	Comb	Oral cleft repair	No	2 months to 2 years
Conrad et al,[86] 2017	Ret	No	S/M	Cleft lip and palate	No	<7 years
de Heer et al,[87] 2017	SA	Yes	NS	Various unspecified	No	<5 years
Glatz et al,[88] 2017	Ret	Yes	S/M	Various unspecified	No	<4 years
Harmsen et al,[89] 2017	Amb	No	Comb	Esophageal atresia repair	Esophageal atresia	<2 years
Hu et al,[90] 2017	Ret	Yes	S/M	Various unspecified	No	<3 years
Ing et al a,[91] 2017	Ret	Yes	S	Various unspecified	No	<5 years
Ing et al b,[92] 2017	Ret	Yes	Comb	Various unspecified	No	< 3 years
Ing et al c,[93] 2017	Ret	Yes	Comb	Various specified	No	<3 years
Lap et al,[94] 2017	Ret	Yes	Comb	Gastroschisis repair	Gastroschisis	Newborn
Nestor et al,[95] 2017	Ret	Yes	S/M	Various unspecified	No	<1 year
Terushkin et al,[96] 2017	Amb	No	M	Laser procedures	No	<4 years
Zhang et al,[97] 2017	PO	Yes	S	Orthopedic surgery	No	6-12 years
Berghmans et al,[98] 2018	PO	No	S	Circumcision	No	18-30 months
Castellheim et al,[99] 2018	Ret	Yes	Comb	Various unspecified	No	0-12 years
Hunt et al,[100] 2018	Ret	Yes	NS	Various specified	Extreme prematurity	Birth hospitalization
Kozanhan et al,[101] 2018	PO	Yes	S	Circumcision	No	7-12 years
Lv et al,[102] 2018	PO	Yes	S	Palatoplasty	No	<2 year
Schneuer et al,[103] 2018	Ret	Yes	S/M/Comb	Various unspecified	No	<4 years
Tsai et al,[104] 2018	Ret	Yes	S/M/Comb	Various unspecified	No	<3 years
Warner et al,[105] 2018	Amb	Yes	S/M	Various specified	No	<3 years
Banerjee et al,[106] 2019	Amb	No	M	Various unspecified	Acute Lymphoblastic Leukemia	Multiple exposures, various ages
Khochfe et al,[107] 2019	Amb	Yes	S	Various unspecified	No	< 2 years
McCann et al,[108] 2019	RCT	Yes	SPM	inguinal hernia	No	<60 weeks
O'Leary et al,[109] 2019	Ret	Yes	Comb	Various unspecified	No	<6 years
Sun et al,[110] 2019	PO	No	SPM	Liver transplant	Biliary atresia	5 months to 2 years
Vedovelli et al,[111] 2019	PO	No	SPM	Heart surgery	Congenital heart disease	<5 years
Warner et al,[112] 2019	Amb	Yes	S/M	Various unspecified	No	<3 years
Zaccariello et al,[113] 2019	SA	Yes	S/M	Various unspecified	No	<3 years

© 2022 Reighard C et al. *JAMA Network Open.*

Han et al,[114] 2020	Ret	No	NS	Surgery for necrotizing enterocolitis	Necrotizing enterocolitis	Infancy
Batta et al,[115] 2020	Ret	No	Comb	Gastrointestinal surgery	Congenital gastrointestinal disease	Neonatal
Feng et al,[116] 2020	Ret	Yes	S/M/Comb	Various specified	No	< 2 years
Hakanson et al,[117] 2020	Ret	Yes	SPM	Open laparotomy	Congenital gastrointestinal disease	< 1 year
Ing et al,[118] 2020	Ret	Yes	S	Various specified	No	< 5 years
Jacola et al,[119] 2020	SA	No	M	Various unspecified	Medulloblastoma	Mean age 10.2 years
Kobayashi et al,[120] 2020	Ret	Yes	S/M	Various unspecified	No	< 1 year
Sedighnejad et al,[121] 2020	Ret	Yes	Comb	Various unspecified	No	< 4 years
Walkden et al,[122] 2020	Ret	Yes	S/M	Various specified	No	< 4 years
Gleich et al,[123] 2021	SA	No	M	Various unspecified	No	< 3 years
Ing et al,[124] 2021	Ret	Yes	SPM	Various specified	No	Prenatal
Lauritzen et al,[125] 2021	SA	No	SPM	Atrial or ventricular septal defect repair	Congenital cardiac disease	Mean age 2.8 years
Partanen et al,[126] 2021	SA	No	M	Various unspecified	Medulloblastoma	Mean age 10.2 years
van Hoorn et al,[127] 2021	Ret	No	Comb	Esophageal atresia repair	Esophageal atresia	Neonatal
Walsh et al,[128] 2021	SA	Yes	Comb	Various specified	Prematurity	Preterm
Warner et al,[129] 2021	SA	Yes	S/M	Various unspecified	No	< 3 years
Zhou et al,[130] 2021	PO	Yes	S/M/Comb	Dental procedures	No	< 7 years

Abbreviations: PO: Prospective observational, Ret: Retrospective, Amb: Ambidirectional, SA: Secondary analysis, RCT: Randomized controlled trial, S: Single exposure, SPM: Single with possible multiple exposures, M: Multiple exposures, Comb: Combined single and multiple exposures, NS: Not specified, PMA: Postmenstrual age

© 2022 Reighard C et al. *JAMA Network Open.*

eTable 6. Outcomes of Duplicate Studies or Studies That Did Not Report Outcome Scores That Could Be Evaluated

		Outcomes Not Used	
**Domain**	**Study**	**Outcome**	**Reason for not using**
Academic	Hansen et al,[50] 2013	Test score (standardized test and teacher rating) test non-attainment	Same control cohort used in Hansen et al. 2011[36]
	Djurhuus et al,[74] 2016	Test score (standardized test and teacher rating) test non-attainment	Same control cohort used in Hansen et al. 2011[36]
	Clausen et al,[85] 2017	Test score (standardized test and teacher rating) test non-attainment	Same control cohort used in Hansen et al. 2011[36]
Behavioral problems	Ing et al b,[57] 2014	CBCL Total Problems, Internalizing, Externalizing	Same control cohort used in Ing et al. 2012[40]
	Ing et al,[118] 2020	CBCL Total Problems, Internalizing, Externalizing	Same control cohort used in Ing et al. 2012[40]
Clinical diagnoses and symptoms	DiMaggio et al,[28] 2009	ICD-9 behavioral and developmental disorders	Cohort used in Ing et al. 2017[92] with larger sample size and longer follow-up
	DiMaggio et al,[34] 2011	ICD-9 behavioral and developmental disorders	Cohort used in Ing et al. 2017[92] with larger sample size and longer follow-up
	Ko et al,[58] 2014	ICD-9 ADHD diagnosis	Cohort used in Tsai et al. 2018[104] with longer follow-up
	Ko et al,[67] 2015	ICD-9 ASD diagnosis	Cohort used in Tsai et al. 2018[104] with longer follow-up
	Feng et al,[116] 2020	ICD-9 DD diagnosis	Cohort used in Tsai et al. 2018[104] with more specific diagnoses
	Ing et al,[118] 2020	ADHD medication use	Cohort used in Ing et al. 2017a[91] with more outcomes assessed
	Sedighnejad et al,[121] 2020	ADHD Diagnosis	Risk ratios cannot be calculated from case-control studies
	Warner et al,[129] 2021	ADHD, Reading, written, and math LD, IEP Speech/Language, IEP Emotion/Behavior	Cohort used in Wilder et al. 2009[31], Flick et al. 2011[35], Sprung et al. 2012[45], and Hu et al. 2017[90]
Executive function	Ing et al b,[57] 2014	SDMT oral, SDMT written	Not overall scores, and cohort used in Ing et al. 2012[40]
	Ing et al,[124] 2021	SDMT oral, SDMT written	Not overall scores, and control cohort used in Ing et al. 2012[40]
General development	O'Leary et al,[109] 2019	EDI Early Developmental Vulnerability, EDI Multiple Challenge Index, Language and Cognitive Development	This study is a subanalysis of cohort in O'Leary et al. 2016[79]
General health and wellbeing	Schneuer et al,[103] 2018	AvEDI Physical Health	Not overall score and actual score not reported
	O'Leary et al,[109] 2019	EDI Physical Health	Not overall score, and this study is a subanalysis of cohort in O'Leary et al. 2016[79]

© 2022 Reighard C et al. *JAMA Network Open.*

Language	Ing et al a,[56] 2014	CELF total score, CELF expressive, CELF receptive	This study is a subanalysis of cohort in Ing et al. 2012[40]
	Ing et al b,[57] 2014	CELF total score, CELF expressive, CELF receptive	Same control cohort used in Ing et al. 2012[40]
	Aun et al,[71] 2016	HK-WISC General Comprehension,	No score data reported, only lists if improvement or decline
	Ing et al b,[92] 2017	CELF total score, CELF expressive, CELF receptive	Same control cohort used in Ing et al. 2012[40]
	O'Leary et al,[109] 2019	EDI communication	Not overall score, and this study is a subanalysis of cohort in O'Leary et al. 2016[79]
	Ing et al,[124] 2021	CELF total score, CELF expressive, CELF receptive	Same control cohort used in Ing et al. 2012[40]
Motor function	Ing et al b ,[57] 2014	MAND	Same control cohort used in Ing et al. 2012[40]
	Ing et al,[124] 2021	MAND	Same control cohort used in Ing et al. 2012[40]
Nonverbal reasoning	Ing et al a,[56] 2014	CPM	Same cohort was used in Ing et al. 2012[40]
	Ing et al b,[57] 2014	CPM	Same control cohort used in Ing et al. 2012[40]
	Ing et al b,[92] 2017	CPM	Same control cohort used in Ing et al. 2012[40]
	Zhang et al,[97] 2017	RSPM	No score data reported, only that there were no differences in IQ
Social-cognition	Walker et al,[33] 2010	Parental Social Emotional Questionnaire	No score data reported
	Schneuer et al,[103] 2018	AvEDI Emotional Health, Social Knowledge	Not overall scores and scores not available
	O'Leary et al,[109] 2019	EDI Emotional Health, EDI Social Knowledge	Not overall scores, and this study is a subanalysis of cohort in O'Leary et al. 2016[79]

Abbreviations: ADHD: Attention-deficit/hyperactivity Disorder, ASD: Autism Spectrum Disorder, AvEDI: Early Development Instrument (Australia), CBCL: Child Behavior Checklist, CELF: Clinical Evaluation of Language Fundamentals, CPM: Raven's Colored Progressive Matrices, EDI: Early Development Instrument, HK-WISC: Hong Kong-Wechsler Intelligence Scale for Children, ICD-9: International Classification of Disease--9th Edition, MAND: McCarren Assessment of Neuromuscular Development RSPM: Raven's Standard Progressive Matrices, SDMT: Symbol Digit Modality Test

© 2022 Reighard C et al. *JAMA Network Open.*

eTable 7. Exposure Data Used From Each of the 31 Included Studies

Study	Any exposure	Single or possible multiple	Single	Multiple
Bartels et al,[27] 2009	Not specified	NA	NA	NA
Wilder et al,[31] 2009	Single	Single	Single	Multiple (2 exposures)
Walker et al,[33] 2010	Single with possible multiple	Single with possible multiple	NA	NA
Flick et al,[35] 2011	Single	Single	Single	Multiple
Hansen et al,[36] 2011	Single with possible multiple	Single with possible multiple	NA	NA
Ing et al,[40] 2012	Combined	Single/NA	Single/NA	Multiple/NA
Sprung et al,[45] 2012	Single	Single	Single	Multiple
Bong et al,[48] 2013	Single with possible multiple	Single with possible multiple	NA	NA
Ing et al a,[56] 2014	Combined	NA	NA	NA
Stratmann et al,[60] 2014	Combined	NA	NA	NA
Williams et al,[61] 2014	Single	Single	Single	NA
Backeljauw et al,[63] 2015	Combined	NA	NA	NA
Bakri et al,[64] 2015	Multiple	NA	NA	Multiple
Davidson et al,[72] 2016	Single with possible multiple	Single with possible multiple	NA	NA
Graham et al,[76] 2016	Single	Single	Single	Multiple
O'Leary et al,[79] 2016	Combined	Single	Single	Multiple (2 exposures)
Sun et al,[82] 2016	Single with possible multiple	Single with possible multiple	NA	NA
de Heer et al,[87] 2017	Not specified	NA	NA	NA
Glatz et al,[88] 2017	Single	Single	Single	Multiple (2 exposures)
Hu et al,[90] 2017	Single	Single	Single	Multiple
Ing et al a,[91] 2017	Single	Single	Single	NA
Nestor et al,[95] 2017	Single	Single	Single	Multiple
Lv et al,[102] 2018	Single	Single	Single	NA
Schneuer et al,[103] 2018	Combined	Single	Single	Multiple (2 exposures)
Tsai et al,[104] 2018	Combined	Single	Single	Multiple
Warner et al,[105] 2018	Single	Single	Single	Multiple
Khochfe et al,[107] 2019	Single	Single	Single	NA
McCann et al,[108] 2019	Single with possible multiple	Single with possible Multiple	NA	NA
Kobayashi et al,[120] 2020	Single	Single	Single	Multiple (2 exposures)
Walkden et al,[122] 2020	Single	Single	Single	Multiple
Zhou et al,[130] 2021	Single	Single	Single	NA
NA: Not applicable				

© 2022 Reighard C et al. *JAMA Network Open.*

eTable 8. Cochrane Risk of Bias Assessment in Randomized Trial

Study	Domain 1: Random sequence generation	Domain 2: Allocation concealment	Domain 3: Blinding of participants and personnel	Domain 4: Blinding of outcome assessment	Domain 5: Incomplete outcome data	Domain 6: Selective reporting
Davidson et al,[72] 2016	Low	Low	High	High	High	Low
McCann et al,[108] 2019	Low	Low	High	High	High	Low

© 2022 Reighard C et al. *JAMA Network Open.*

eTable 9. Risk of Bias In Nonrandomized Studies or Interventions (ROBINS-I) Assessment of Eligible Nonrandomized Studies

Study	Domain 1: Confounding	Domain : Selection	Domain 3: Classification of intervention	Domain 4: Deviation from interventions	Domain 5: Missing data	Domain 6: Measurement of outcomes	Domain 7: Selection of reported result	ROBINS-I overall
Bartels et al, [27] 2009	Moderate	Low	Serious	Moderate	Serious	Low	Low	Serious
Wilder et al,[31] 2009	Serious	Low	Moderate	Moderate	Serious	Low	Low	Serious
Walker et al,[33] 2010	Serious	Low	Low	Moderate	Low	Low	Low	Serious
Flick et al,[35] 2011	Moderate	Low	Moderate	Moderate	Serious	Low	Low	Serious
Hansen et al,[36] 2011	Moderate	Low	Moderate	Moderate	Low	Low	Low	Moderate
Ing et al,[40] 2012	Moderate	Low	Moderate	Moderate	Low	Low	Low	Moderate
Sprung et al,[45] 2012	Moderate	Low	Moderate	Moderate	Serious	Low	Low	Serious
Bong et al,[48] 2013	Moderate	Low	Moderate	Moderate	Moderate	Low	Low	Moderate
Ing et al a,[56] 2014	Moderate	Low	Moderate	Moderate	Low	Low	Low	Moderate
Stratmann et al,[60] 2014	Serious	Serious	Moderate	Moderate	Low	Moderate	Low	Serious
Williams et al,[61] 2014	Moderate	Low	Moderate	Moderate	Low	Low	Low	Moderate
Backeljauw et al,[63] 2015	Moderate	Moderate	Moderate	Moderate	Low	Low	Moderate	Moderate
Bakri et al,[64] 2015	Serious	Moderate	Moderate	Moderate	Low	Moderate	Low	Serious
Graham et al,[76] 2016	Moderate	Moderate	Moderate	Moderate	Moderate	Low	Moderate	Moderate
O'Leary et al,[79] 2016	Moderate	Moderate	Moderate	Moderate	Moderate	Low	Low	Moderate
Sun et al,[82] 2016	Moderate	Moderate	Moderate	Moderate	Low	Moderate	Low	Moderate
de Heer et al,[87] 2017	Serious	Serious	Serious	Moderate	Serious	Low	Moderate	Serious
Glatz et al,[88] 2017	Moderate	Low	Moderate	Moderate	Low	Low	Low	Moderate
Hu et al,[90] 2017	Serious	Low	Moderate	Moderate	Low	Low	Low	Serious
Ing et al a,[91] 2017	Moderate	Low	Moderate	Moderate	Moderate	Moderate	Low	Moderate
Nestor et al,[95] 2017	Serious	Moderate	Moderate	Moderate	Moderate	None	Low	Serious
Lv et al,[102] 2018	Serious	None	Moderate	Moderate	None	Serious	Low	Serious
Schneuer et al,[103] 2018	Serious	Moderate	Moderate	Moderate	Low	Moderate	Moderate	Serious
Tsai et al,[104] 2018	Serious	Low	Moderate	Moderate	Moderate	Moderate	Low	Serious
Warner et al,[105] 2018	Moderate	Moderate	Moderate	Moderate	Low	Moderate	Low	Moderate
Khochfe et al,[107] 2019	Serious	Moderate	Moderate	Moderate	None	Serious	Low	Serious
Kobayashi et al,[120] 2020	Moderate	Low	Moderate	Moderate	Moderate	Moderate	Low	Moderate

© 2022 Reighard C et al. *JAMA Network Open.*

Walkden et al,[122] 2020	Moderate	Low	Moderate	Moderate	Moderate	Low	Low	Moderate
Zhou et al,[130] 2021	Serious	Low	Low	Low	Moderate	Low	Low	Serious

© 2022 Reighard C et al. *JAMA Network Open.*

eFigure 1. Domain-Specific Meta-analysis of Scores After Single With Possible Multiple Exposure to Surgery and Anesthesia

Study or Subgroup	Std. Mean Difference	SE	Exposed Total	Unexposed Total	Weight	Std. Mean Difference IV, Random, 95% CI
**2.1.1 Academics**						
Flick et al,[35] 2011	0.092	0.075	244	638	10.5%	0.09 [−0.05, 0.24]
Hansen et al,[36] 2011	−0.038	0.024	2016	11850	14.6%	−0.04 [−0.09, 0.01]
Bong et al,[48] 2013	0.074	0.142	96	104	5.8%	0.07 [−0.20, 0.35]
Williams et al,[61] 2014	−0.587	0.073	246	738	10.7%	−0.59 [−0.73, −0.44]
Glatz et al,[88] 2017	−0.017	0.006	33514	159619	15.2%	−0.02 [−0.03, −0.01]
Hu et al,[90] 2017	−0.02	0.076	345	351	10.4%	−0.02 [−0.17, 0.13]
Warner et al,[105] 2018	−0.298	0.072	380	411	10.8%	−0.30 [−0.44, −0.16]
McCann et al,[108] 2019	−0.011	0.09	275	220	9.3%	−0.01 [−0.19, 0.17]
Walkden et al,[122] 2020	0	0.051	830	8792	12.6%	0.00 [−0.10, 0.10]
**Subtotal (95% CI)**			**37946**	**182723**	**100.0%**	**−0.09 [−0.18, −0.01]**
Heterogeneity: $Tau^2 = 0.01$; $Chi^2 = 78.70$, df = 8 (P < 0.001); $I^2 = 90\%$						
Test for overall effect: Z = 2.11 (P = 0.03)						
**2.1.2 Behavioral problems**						
Sun et al,[82] 2016	−0.365	0.142	101	101	12.7%	−0.36 [−0.64, −0.09]
Warner et al,[105] 2018	−0.106	0.071	380	411	27.6%	−0.11 [−0.25, 0.03]
McCann et al,[108] 2019	−0.133	0.079	356	287	25.3%	−0.13 [−0.29, 0.02]
Walkden et al,[122] 2020	0	0.05	850	7042	34.4%	0.00 [−0.10, 0.10]
**Subtotal (95% CI)**			**1687**	**7841**	**100.0%**	**−0.11 [−0.23, 0.01]**
Heterogeneity: $Tau^2 = 0.01$; $Chi^2 = 7.15$, df = 3 (P = 0.07); $I^2 = 58\%$						
Test for overall effect: Z = 1.83 (P = 0.07)						
**2.1.3 Cognition**						
Walker et al,[33] 2010	−0.379	0.168	43	211	0.4%	−0.38 [−0.71, −0.05]
Flick et al,[35] 2011	−0.04	0.083	200	529	1.6%	−0.04 [−0.20, 0.12]
Sun et al,[82] 2016	0.02	0.138	105	105	0.6%	0.02 [−0.25, 0.29]
Glatz et al,[88] 2017	−0.027	0.011	9198	45115	88.3%	−0.03 [−0.05, −0.01]
Lv et al,[102] 2018	0.139	0.147	86	100	0.5%	0.14 [−0.15, 0.43]
Warner et al,[105] 2018	−0.028	0.071	380	411	2.1%	−0.03 [−0.17, 0.11]
McCann et al,[108] 2019	−0.013	0.079	356	287	1.7%	−0.01 [−0.17, 0.14]
Walkden et al,[122] 2020	0	0.051	648	5073	4.1%	0.00 [−0.10, 0.10]
Zhou et al,[130] 2021	−0.053	0.121	129	144	0.7%	−0.05 [−0.29, 0.18]
**Subtotal (95% CI)**			**11145**	**51975**	**100.0%**	**−0.03 [−0.05, −0.01]**
Heterogeneity: $Tau^2 = 0.00$; $Chi^2 = 6.16$, df = 8 (P = 0.63); $I^2 = 0\%$						
Test for overall effect: Z = 2.54 (P = 0.01)						
**2.1.4 Executive function**						
Sun et al,[82] 2016	−0.061	0.139	104	104	15.4%	−0.06 [−0.33, 0.21]
Warner et al,[105] 2018	−0.284	0.072	380	411	45.1%	−0.28 [−0.43, −0.14]
McCann et al,[108] 2019	−0.165	0.079	356	287	39.4%	−0.17 [−0.32, −0.01]
**Subtotal (95% CI)**			**840**	**802**	**100.0%**	**−0.20 [−0.32, −0.09]**
Heterogeneity: $Tau^2 = 0.00$; $Chi^2 = 2.53$, df = 2 (P = 0.28); $I^2 = 21\%$						
Test for overall effect: Z = 3.52 (P < 0.001)						
**2.1.5 General development**						
Graham et al,[76] 2016	−0.117	0.018	3850	13586	32.7%	−0.12 [−0.15, −0.08]
O'Leary et al,[79] 2016	−0.022	0.011	22812	55910	35.0%	−0.02 [−0.04, −0.00]
Schneuer et al,[103] 2018	−0.087	0.019	11006	69308	32.3%	−0.09 [−0.12, −0.05]
**Subtotal (95% CI)**			**37668**	**138804**	**100.0%**	**−0.07 [−0.14, −0.01]**
Heterogeneity: $Tau^2 = 0.00$; $Chi^2 = 23.70$, df = 2 (P < 0.001); $I^2 = 92\%$						
Test for overall effect: Z = 2.36 (P = 0.02)						
**2.1.6 Language**						
Ing et al,[40] 2012	−0.473	0.133	141	1054	7.4%	−0.47 [−0.73, −0.21]
Sun et al,[82] 2016	−0.05	0.138	105	105	7.0%	−0.05 [−0.32, 0.22]
Hu et al,[90] 2017	−0.142	0.074	358	370	14.9%	−0.14 [−0.29, 0.00]
Warner et al,[105] 2018	0.018	0.071	380	411	15.4%	0.02 [−0.12, 0.16]
McCann et al,[108] 2019	−0.042	0.079	356	287	14.0%	−0.04 [−0.20, 0.11]
Kobayashi et al,[120] 2020	−0.016	0.082	746	63234	13.5%	−0.02 [−0.18, 0.14]
Walkden et al,[122] 2020	−0.1	0.051	403	3192	19.5%	−0.10 [−0.20, −0.00]
Zhou et al,[130] 2021	0.062	0.121	129	144	8.4%	0.06 [−0.18, 0.30]
**Subtotal (95% CI)**			**2618**	**68797**	**100.0%**	**−0.08 [−0.16, 0.00]**
Heterogeneity: $Tau^2 = 0.01$; $Chi^2 = 13.74$, df = 7 (P = 0.06); $I^2 = 49\%$						
Test for overall effect: Z = 1.87 (P = 0.06)						
**2.1.7 Nonverbal reasoning**						
Ing et al,[40] 2012	−0.302	0.143	141	1413	26.9%	−0.30 [−0.58, −0.02]
Sun et al,[82] 2016	0.043	0.138	105	105	28.0%	0.04 [−0.23, 0.31]
McCann et al,[108] 2019	0.011	0.079	356	287	45.1%	0.01 [−0.14, 0.17]
**Subtotal (95% CI)**			**602**	**1805**	**100.0%**	**−0.06 [−0.26, 0.13]**
Heterogeneity: $Tau^2 = 0.01$; $Chi^2 = 4.14$, df = 2 (P = 0.13); $I^2 = 52\%$						
Test for overall effect: Z = 0.66 (P = 0.51)						

Std. Mean Difference IV, Random, 95% CI

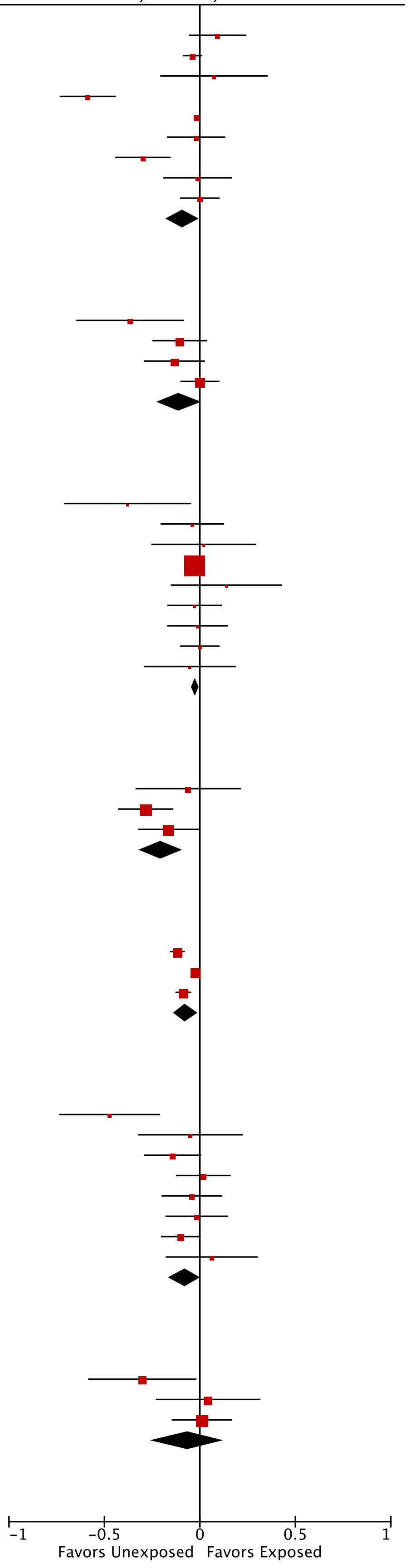

© 2022 Reighard C et al. *JAMA Network Open.*

eFigure 2. Meta-analysis of Hazard and Risk of Clinical Diagnoses and Symptoms After Single With Possible Multiple Exposure to Surgery and Anesthesia

A

Study or Subgroup	log[Hazard Ratio]	SE	Exposed Total	Unexposed Total	Weight	Hazard Ratio IV, Random, 95% CI
**2.2.1 Clinical diagnoses and symptoms – neurodevelopmental disorders**						
Wilder et al,[31] 2009	0	0.121	449	4764	15.4%	1.00 [0.79, 1.27]
Flick et al,[35] 2011	0.058	0.163	138	700	9.7%	1.06 [0.77, 1.46]
Sprung et al,[45] 2012	0.166	0.206	286	5007	6.5%	1.18 [0.79, 1.77]
Hu et al,[90] 2017	0.058	0.199	457	463	6.9%	1.06 [0.72, 1.57]
Ing et al,[91] 2017a	0.231	0.016	38493	192465	52.5%	1.26 [1.22, 1.30]
Tsai et al,[104] 2018	0	0.173	804	3438	8.8%	1.00 [0.71, 1.40]
**Subtotal (95% CI)**			**40627**	**206837**	**100.0%**	**1.15 [1.03, 1.29]**

Heterogeneity: $Tau^2 = 0.01$; $Chi^2 = 7.09$, df = 5 (P = 0.21); $I^2 = 30\%$
Test for overall effect: Z = 2.53 (P = 0.01)

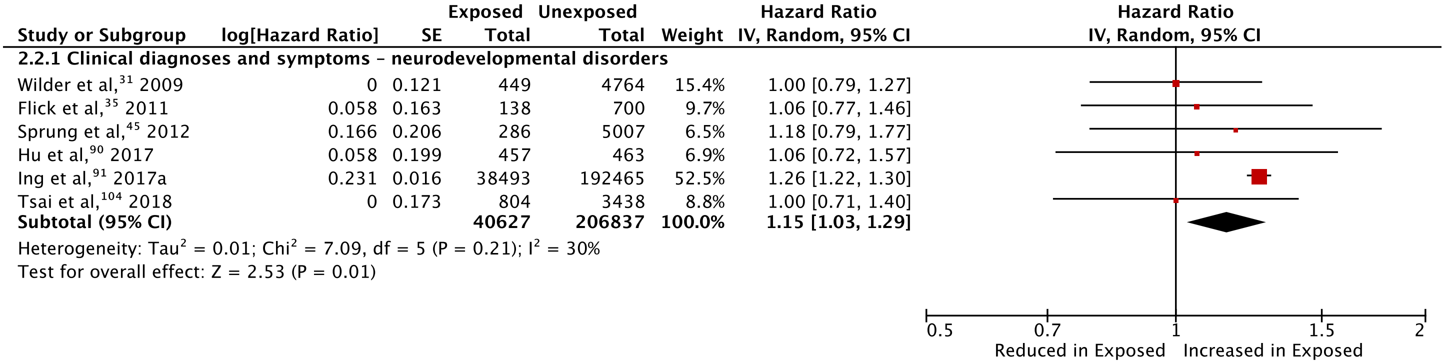

B

Study or Subgroup	log[Risk Ratio]	SE	Exposed Total	Unexposed Total	Weight	Risk Ratio IV, Random, 95% CI
**2.3.1 Clinical diagnoses and symptoms – neurodevelopmental disorders**						
Bong et al,[48] 2013	1.38	0.545	100	106	16.9%	3.97 [1.37, 11.57]
Nestor et al,[95] 2017	0.699	0.279	171	129	58.0%	2.01 [1.16, 3.48]
McCann et al,[108] 2019	0.357	0.443	251	211	25.1%	1.43 [0.60, 3.41]
**Subtotal (95% CI)**			**522**	**446**	**100.0%**	**2.07 [1.32, 3.24]**

Heterogeneity: $Tau^2 = 0.01$; $Chi^2 = 2.14$, df = 2 (P = 0.34); $I^2 = 7\%$
Test for overall effect: Z = 3.19 (P = 0.001)

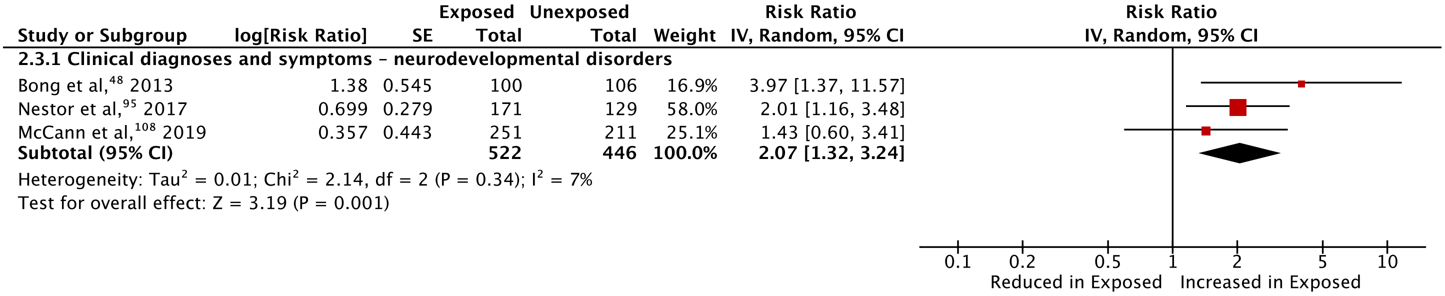

Legend: Panel A: Studies reporting hazard ratios for neurodevelopmental disorder diagnoses after *Single with possible multiple exposure* to surgery and anesthesia. Panel B: Studies reporting odds or risk ratios for neurodevelopmental disorder diagnoses after *Single with possible multiple exposure* to surgery and anesthesia

© 2022 Reighard C et al. *JAMA Network Open.*

eFigure 3. Domain-Specific Meta-analysis of Subdomain Scores and Hazard of ADHD After Single With Possible Multiple Exposure to Surgery and Anesthesia

A

Study or Subgroup	Std. Mean Difference	SE	Exposed Total	Unexposed Total	Weight	Std. Mean Difference IV, Random, 95% CI
**2.4.1 Internalizing behavioral problems**						
Sun et al,[82] 2016	−0.421	0.142	102	102	21.7%	−0.42 [−0.70, −0.14]
Warner et al,[105] 2018	−0.067	0.071	380	411	40.5%	−0.07 [−0.21, 0.07]
McCann et al,[108] 2019	−0.121	0.079	356	287	37.9%	−0.12 [−0.28, 0.03]
**Subtotal (95% CI)**			**838**	**800**	**100.0%**	**−0.16 [−0.33, 0.00]**
Heterogeneity: Tau^2 = 0.01; Chi^2 = 5.00, df = 2 (P = 0.08); I^2 = 60%						
Test for overall effect: Z = 1.96 (P = 0.05)						
**2.4.2 Externalizing behavioral problems**						
Sun et al,[82] 2016	−0.277	0.141	101	101	23.9%	−0.28 [−0.55, −0.00]
Warner et al,[105] 2018	−0.125	0.071	380	411	28.4%	−0.13 [−0.26, 0.01]
Khochfe et al,[107] 2019	−1.11	0.198	168	226	19.8%	−1.11 [−1.50, −0.72]
McCann et al,[108] 2019	−0.119	0.079	356	287	27.9%	−0.12 [−0.27, 0.04]
**Subtotal (95% CI)**			**1005**	**1025**	**100.0%**	**−0.35 [−0.65, −0.06]**
Heterogeneity: Tau^2 = 0.07; Chi^2 = 23.59, df = 3 (P < 0.001); I^2 = 87%						
Test for overall effect: Z = 2.37 (P = 0.02)						
**2.4.3 Fine motor function**						
Walker et al,[33] 2010	−0.494	0.169	43	211	5.0%	−0.49 [−0.83, −0.16]
Davidson et al,[72] 2016	−0.037	0.088	287	234	15.3%	−0.04 [−0.21, 0.14]
Sun et al,[82] 2016	−0.079	0.14	102	102	7.0%	−0.08 [−0.35, 0.20]
Warner et al,[105] 2018	−0.073	0.071	380	411	20.8%	−0.07 [−0.21, 0.07]
Kobayashi et al,[120] 2020	−0.032	0.07	746	63234	21.2%	−0.03 [−0.17, 0.11]
Walkden et al,[122] 2020	−0.1	0.051	359	3012	30.8%	−0.10 [−0.20, −0.00]
**Subtotal (95% CI)**			**1917**	**67204**	**100.0%**	**−0.09 [−0.17, −0.01]**
Heterogeneity: Tau^2 = 0.00; Chi^2 = 6.84, df = 5 (P = 0.23); I^2 = 27%						
Test for overall effect: Z = 2.25 (P = 0.02)						
**2.4.4 Gross motor function**						
Walker et al,[33] 2010	−0.465	0.169	43	211	9.5%	−0.47 [−0.80, −0.13]
Davidson et al,[72] 2016	−0.04	0.089	279	234	22.6%	−0.04 [−0.21, 0.13]
Kobayashi et al,[120] 2020	−0.205	0.057	746	63234	32.9%	−0.20 [−0.32, −0.09]
Walkden et al,[122] 2020	−0.1	0.051	359	3029	35.1%	−0.10 [−0.20, −0.00]
**Subtotal (95% CI)**			**1427**	**66708**	**100.0%**	**−0.16 [−0.27, −0.04]**
Heterogeneity: Tau^2 = 0.01; Chi^2 = 6.86, df = 3 (P = 0.08); I^2 = 56%						
Test for overall effect: Z = 2.68 (P = 0.007)						

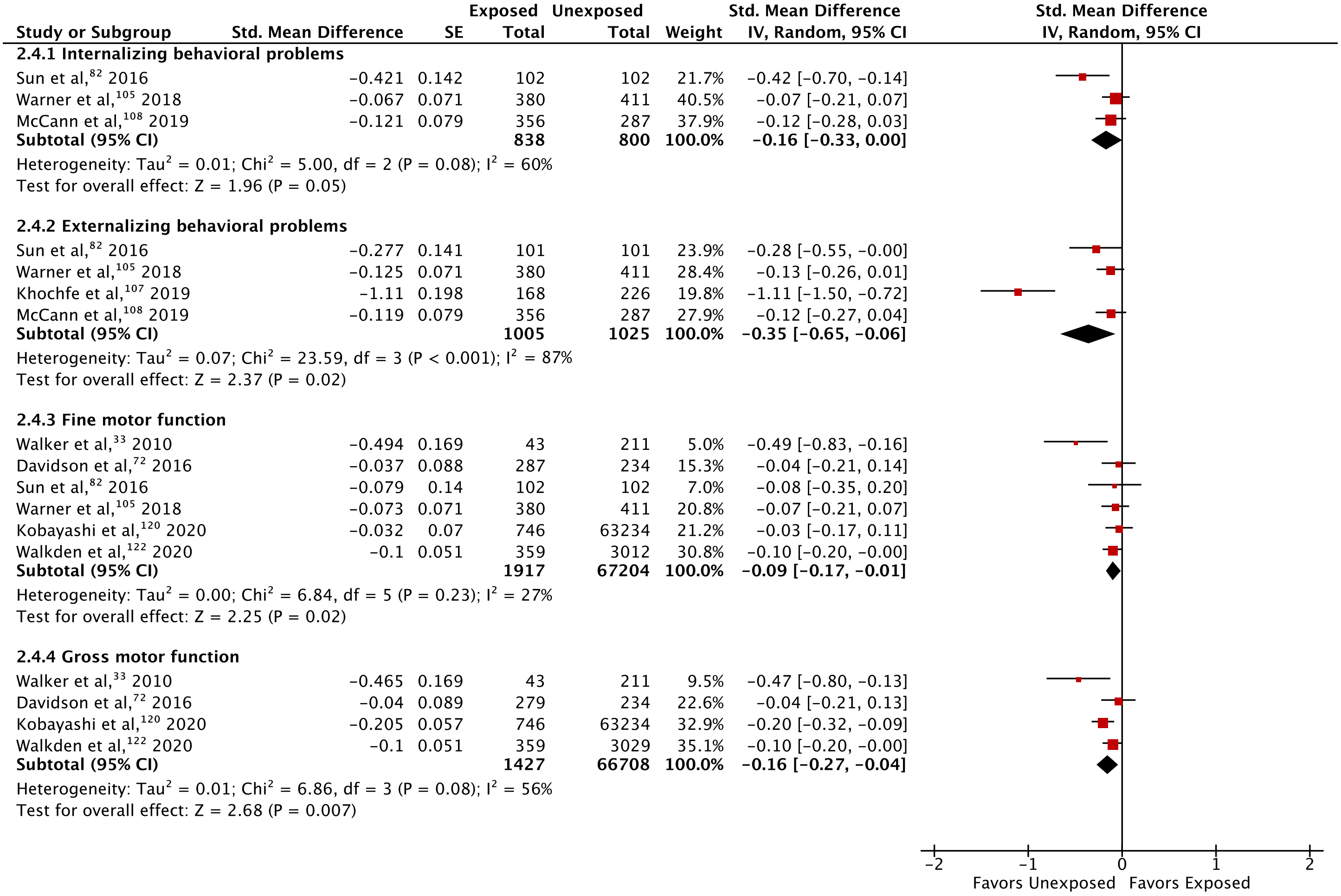

B

Study or Subgroup	log[Hazard Ratio]	SE	Exposed Total	Unexposed Total	Weight	Hazard Ratio IV, Random, 95% CI
**2.5.1 Diagnosis of attention deficit hyperactivity disorder**						
Sprung et al,[45] 2012	0.166	0.206	286	5007	1.2%	1.18 [0.79, 1.77]
Hu et al,[90] 2017	0.285	0.19	457	463	1.4%	1.33 [0.92, 1.93]
Ing et al,[91] 2017a	0.27	0.023	42381	211905	95.5%	1.31 [1.25, 1.37]
Tsai et al,[104] 2018	0.095	0.162	804	3438	1.9%	1.10 [0.80, 1.51]
**Subtotal (95% CI)**			**43928**	**220813**	**100.0%**	**1.30 [1.25, 1.36]**
Heterogeneity: Tau^2 = 0.00; Chi^2 = 1.39, df = 3 (P = 0.71); I^2 = 0%						
Test for overall effect: Z = 11.82 (P < 0.001)						

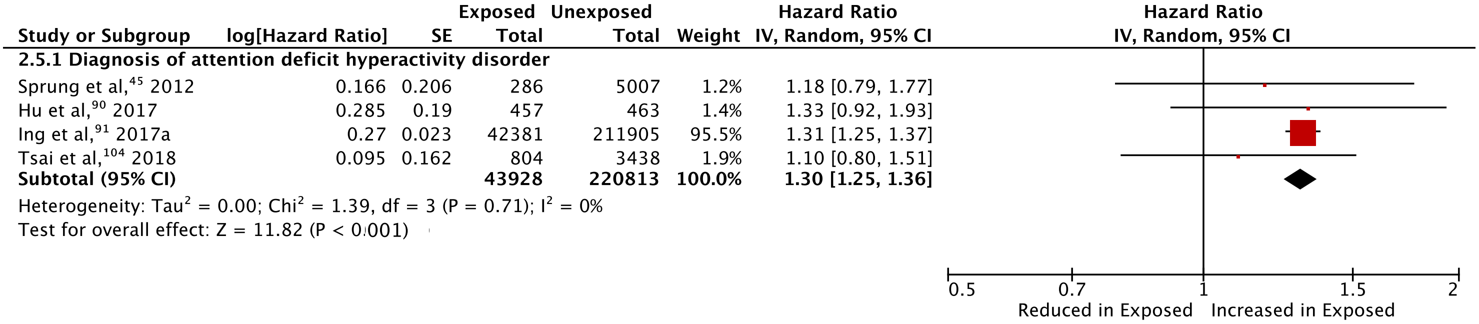

Legend: Panel A: Studies reporting standardized mean differences of subdomains of behavioral and motor problems after *Single with possible multiple exposure* to surgery and anesthesia. Panel B: Studies reporting hazard ratios for ADHD diagnosis after *Single with possible multiple exposure* to surgery and anesthesia

© 2022 Reighard C et al. *JAMA Network Open.*

eFigure 4. Domain-Specific Meta-analysis of Scores and Hazard of Clinical Diagnoses and Symptoms After Single Exposure to Surgery and Anesthesia

A

Study or Subgroup	Std. Mean Difference	SE	Exposed Total	Unexposed Total	Weight	Std. Mean Difference IV, Random, 95% CI
**3.1.1 Academics**						
Flick et al,[35] 2011	0.092	0.075	244	638	16.0%	0.09 [−0.05, 0.24]
Williams et al,[61] 2014	−0.587	0.073	246	738	16.1%	−0.59 [−0.73, −0.44]
Glatz et al,[88] 2017	−0.017	0.006	33514	159619	18.6%	−0.02 [−0.03, −0.01]
Hu et al,[90] 2017	−0.02	0.076	345	351	15.9%	−0.02 [−0.17, 0.13]
Warner et al,[105] 2018	−0.298	0.072	380	411	16.1%	−0.30 [−0.44, −0.16]
Walkden et al,[122] 2020	0	0.051	830	8792	17.3%	0.00 [−0.10, 0.10]
**Subtotal (95% CI)**			**35559**	**170549**	**100.0%**	**−0.13 [−0.29, 0.02]**
Heterogeneity: Tau^2 = 0.03; Chi^2 = 77.79, df = 5 (P < 0.001); I^2 = 94%						
Test for overall effect: Z = 1.70 (P = 0.09)						
**3.1.2 Cognition**						
Flick et al,[35] 2011	−0.04	0.083	200	529	1.6%	−0.04 [−0.20, 0.12]
Glatz et al,[88] 2017	−0.027	0.011	9198	45115	90.7%	−0.03 [−0.05, −0.01]
Lv et al,[102] 2018	0.139	0.147	86	100	0.5%	0.14 [−0.15, 0.43]
Warner et al,[105] 2018	−0.028	0.071	380	411	2.2%	−0.03 [−0.17, 0.11]
Walkden et al,[122] 2020	0	0.051	648	5073	4.2%	0.00 [−0.10, 0.10]
Zhou et al,[130] 2021	−0.053	0.121	129	144	0.7%	−0.05 [−0.29, 0.18]
**Subtotal (95% CI)**			**10641**	**51372**	**100.0%**	**−0.03 [−0.05, −0.00]**
Heterogeneity: Tau^2 = 0.00; Chi^2 = 1.60, df = 5 (P = 0.90); I^2 = 0%						
Test for overall effect: Z = 2.43 (P = 0.02)						
**3.1.3 General development**						
Graham et al,[76] 2016	−0.117	0.018	3850	13586	32.7%	−0.12 [−0.15, −0.08]
O'Leary et al,[79] 2016	−0.022	0.011	22812	55910	35.0%	−0.02 [−0.04, −0.00]
Schneuer et al,[103] 2018	−0.087	0.019	11006	69308	32.3%	−0.09 [−0.12, −0.05]
**Subtotal (95% CI)**			**37668**	**138804**	**100.0%**	**−0.07 [−0.14, −0.01]**
Heterogeneity: Tau^2 = 0.00; Chi^2 = 23.70, df = 2 (P < 0.001); I^2 = 92%						
Test for overall effect: Z = 2.36 (P = 0.02)						
**3.1.4 Language**						
Ing et al,[40] 2012	−0.473	0.13320551	141	1054	10.5%	−0.47 [−0.73, −0.21]
Hu et al,[90] 2017	−0.142	0.074	358	370	18.6%	−0.14 [−0.29, 0.00]
Warner et al,[105] 2018	0.018	0.071	380	411	19.1%	0.02 [−0.12, 0.16]
Kobayashi et al,[120] 2020	−0.016	0.082	746	63234	17.3%	−0.02 [−0.18, 0.14]
Walkden et al,[122] 2020	−0.1	0.051	403	3192	22.7%	−0.10 [−0.20, −0.00]
Zhou et al,[130] 2021	0.062	0.121	129	144	11.8%	0.06 [−0.18, 0.30]
**Subtotal (95% CI)**			**2157**	**68405**	**100.0%**	**−0.09 [−0.20, 0.02]**
Heterogeneity: Tau^2 = 0.01; Chi^2 = 13.45, df = 5 (P = 0.02); I^2 = 63%						
Test for overall effect: Z = 1.66 (P = 0.10)						

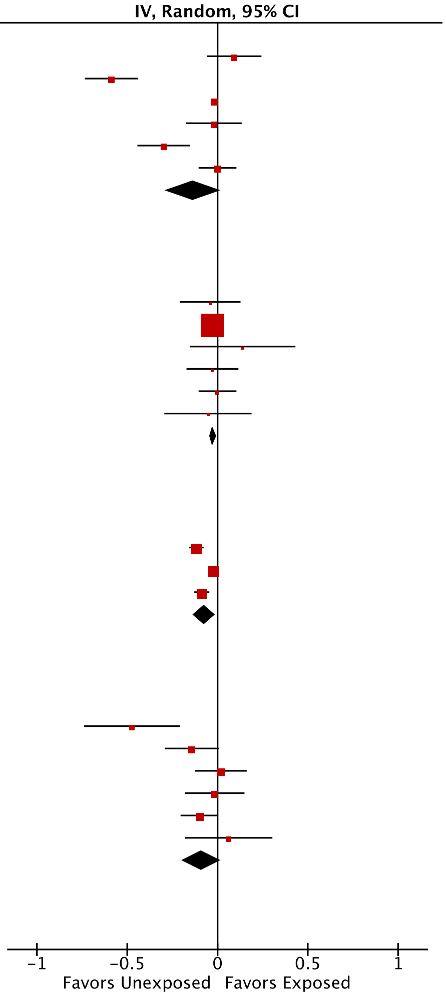

B

Study or Subgroup	log[Hazard Ratio]	SE	Exposed Total	Unexposed Total	Weight	Hazard Ratio IV, Random, 95% CI
**3.2.1 Clinical diagnoses and symptoms – neurodevelopmental disorders**						
Wilder et al,[31] 2009	0	0.121	449	4764	15.4%	1.00 [0.79, 1.27]
Flick et al,[35] 2011	0.058	0.163	138	700	9.7%	1.06 [0.77, 1.46]
Sprung et al,[45] 2012	0.166	0.206	286	5007	6.5%	1.18 [0.79, 1.77]
Hu et al,[90] 2017	0.058	0.199	457	463	6.9%	1.06 [0.72, 1.57]
Ing et al,[91] 2017a	0.231	0.016	38493	192465	52.5%	1.26 [1.22, 1.30]
Tsai et al,[104] 2018	0	0.173	804	3438	8.8%	1.00 [0.71, 1.40]
**Subtotal (95% CI)**			**40627**	**206837**	**100.0%**	**1.15 [1.03, 1.29]**
Heterogeneity: Tau^2 = 0.01; Chi^2 = 7.09, df = 5 (P = 0.21); I^2 = 30%						
Test for overall effect: Z = 2.53 (P = 0.01)						

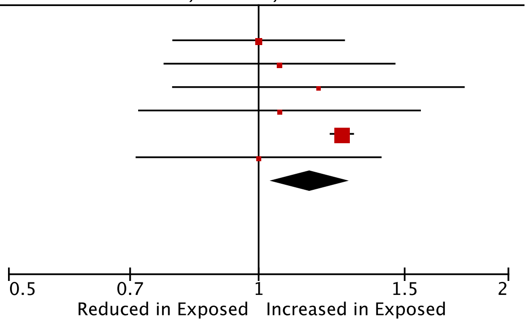

Legend: Panel A: Studies reporting standardized mean differences of domain scores after Single exposure to surgery and anesthesia. Panel B: Studies reporting hazard ratios for neurodevelopmental disorder diagnoses after Single exposure to surgery and anesthesia

© 2022 Reighard C et al. *JAMA Network Open.*

eFigure 5. Domain-Specific Meta-analysis of Subdomain Scores and Hazard of ADHD After Single Exposure to Surgery and Anesthesia

A

Study or Subgroup	Std. Mean Difference	SE	Exposed Total	Unexposed Total	Weight	Std. Mean Difference IV, Random, 95% CI
**3.3.1 Fine motor function**						
Warner et al,[105] 2018	−0.073	0.071	380	411	25.2%	−0.07 [−0.21, 0.07]
Kobayashi et al,[120] 2020	−0.032	0.07	746	63234	25.9%	−0.03 [−0.17, 0.11]
Walkden et al,[122] 2020	−0.1	0.051	359	3012	48.9%	−0.10 [−0.20, −0.00]
**Subtotal (95% CI)**			**1485**	**66657**	**100.0%**	**−0.08 [−0.15, −0.01]**

Heterogeneity: Tau^2 = 0.00; Chi^2 = 0.62, df = 2 (P = 0.73); I^2 = 0%
Test for overall effect: Z = 2.12 (P = 0.03)

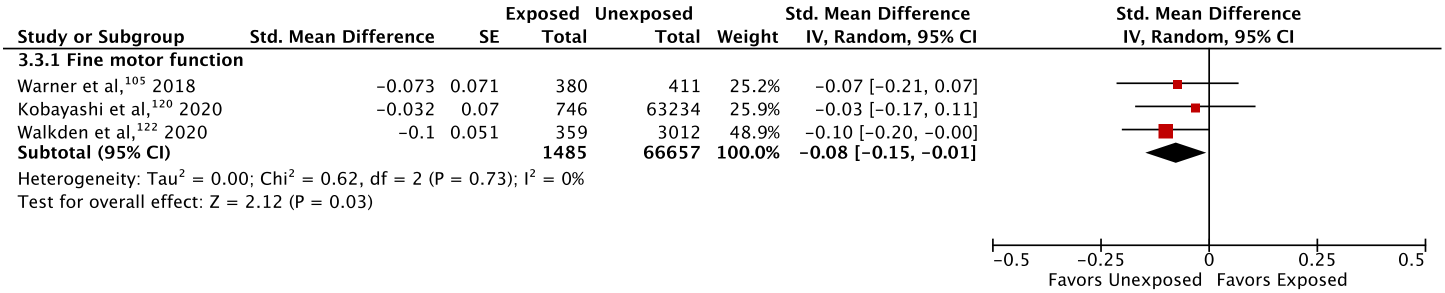

B

Study or Subgroup	log[Hazard Ratio]	SE	Exposed Total	Unexposed Total	Weight	Hazard Ratio IV, Random, 95% CI
**3.4.1 Diagnosis of attention deficit hyperactivity disorder**						
Sprung et al,[45] 2012	0.166	0.206	286	5007	1.2%	1.18 [0.79, 1.77]
Hu et al,[90] 2017	0.285	0.19	457	463	1.4%	1.33 [0.92, 1.93]
Ing et al,[91] 2017a	0.27	0.023	42381	211905	95.7%	1.31 [1.25, 1.37]
Tsai et al,[104] 2018	0	0.173	804	3438	1.7%	1.00 [0.71, 1.40]
**Subtotal (95% CI)**			**43928**	**220813**	**100.0%**	**1.30 [1.25, 1.36]**

Heterogeneity: Tau^2 = 0.00; Chi^2 = 2.63, df = 3 (P = 0.45); I^2 = 0%
Test for overall effect: Z = 11.75 (P < 0.001)

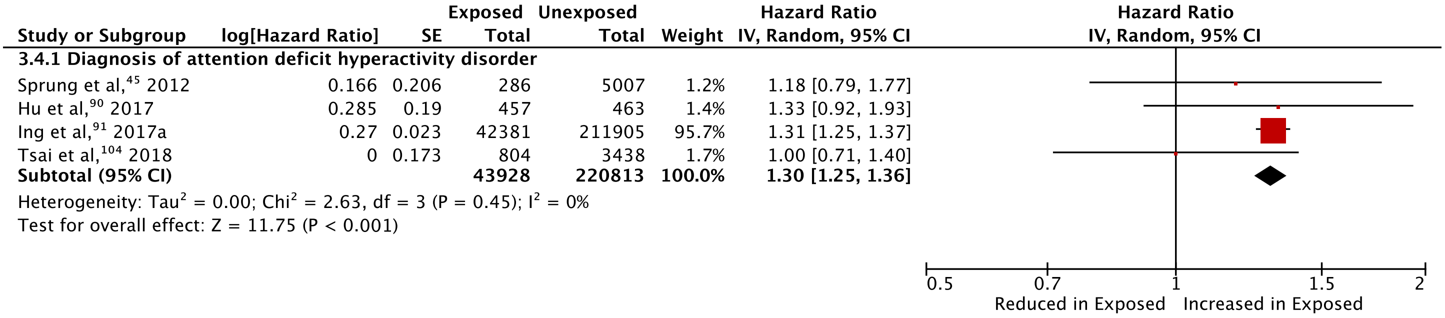

Legend: Panel A: Studies reporting standardized mean differences of subdomains of motor problems after *Single exposure* to surgery and anesthesia. Panel B: Studies reporting hazard ratios for ADHD diagnosis after *Single exposure* to surgery and anesthesia

© 2022 Reighard C et al. *JAMA Network Open.*

eFigure 6. Domain-Specific Meta-analysis of Scores and Hazard of Clinical Diagnoses and Symptoms After Multiple Exposure to Surgery and Anesthesia

A

Study or Subgroup	Std. Mean Difference	SE	Exposed Total	Unexposed Total	Weight	Std. Mean Difference IV, Random, 95% CI
**4.1.1 Academics**						
Flick et al,[35] 2011	−0.312	0.142	54	638	11.1%	−0.31 [−0.59, −0.03]
Hu et al,[90] 2017	−0.271	0.117	92	351	14.2%	−0.27 [−0.50, −0.04]
Glatz et al,[88] 2017	−0.063	0.021	2841	14205	33.7%	−0.06 [−0.10, −0.02]
Warner et al,[105] 2018	−0.271	0.086	206	411	19.6%	−0.27 [−0.44, −0.10]
Walkden et al,[122] 2020	−0.05	0.077	156	8792	21.5%	−0.05 [−0.20, 0.10]
**Subtotal (95% CI)**			**3349**	**24397**	**100.0%**	**−0.16 [−0.27, −0.05]**
Heterogeneity: $Tau^2 = 0.01$; $Chi^2 = 11.05$, df = 4 (P = 0.03); $I^2 = 64\%$						
Test for overall effect: Z = 2.77 (P = 0.006)						
**4.1.2 Cognition**						
Flick et al,[35] 2011	−0.304	0.164	40	529	4.0%	−0.30 [−0.63, 0.02]
Glatz et al,[88] 2017	−0.028	0.034	887	45115	65.2%	−0.03 [−0.09, 0.04]
Warner et al,[105] 2018	−0.086	0.085	206	411	14.0%	−0.09 [−0.25, 0.08]
Walkden et al,[122] 2020	0	0.077	130	5073	16.8%	0.00 [−0.15, 0.15]
**Subtotal (95% CI)**			**1263**	**51128**	**100.0%**	**−0.04 [−0.11, 0.02]**
Heterogeneity: $Tau^2 = 0.00$; $Chi^2 = 3.28$, df = 3 (P = 0.35); $I^2 = 8\%$						
Test for overall effect: Z = 1.29 (P = 0.20)						
**4.1.3 General development**						
Graham et al,[76] 2016	−0.158	0.041	620	13586	31.6%	−0.16 [−0.24, −0.08]
O'Leary et al,[79] 2016	−0.032	0.024	4167	55910	40.3%	−0.03 [−0.08, 0.02]
Schneuer et al,[103] 2018	−0.077	0.048	1515	69308	28.2%	−0.08 [−0.17, 0.02]
**Subtotal (95% CI)**			**6302**	**138804**	**100.0%**	**−0.08 [−0.16, −0.01]**
Heterogeneity: $Tau^2 = 0.00$; $Chi^2 = 7.09$, df = 2 (P = 0.03); $I^2 = 72\%$						
Test for overall effect: Z = 2.10 (P = 0.04)						
**4.1.4 Language**						
Ing et al,[40] 2012	−0.544	0.258	40	1054	9.1%	−0.54 [−1.05, −0.04]
Hu et al,[90] 2017	−0.328	0.115	97	370	21.9%	−0.33 [−0.55, −0.10]
Warner et al,[105] 2018	−0.073	0.085	206	411	26.0%	−0.07 [−0.24, 0.09]
Kobayashi et al,[120] 2020	−0.087	0.203	90	63234	12.6%	−0.09 [−0.48, 0.31]
Walkden et al,[122] 2020	−0.4	0.051	88	3192	30.5%	−0.40 [−0.50, −0.30]
**Subtotal (95% CI)**			**521**	**68261**	**100.0%**	**−0.27 [−0.45, −0.09]**
Heterogeneity: $Tau^2 = 0.02$; $Chi^2 = 12.94$, df = 4 (P = 0.01); $I^2 = 69\%$						
Test for overall effect: Z = 3.01 (P = 0.003)						

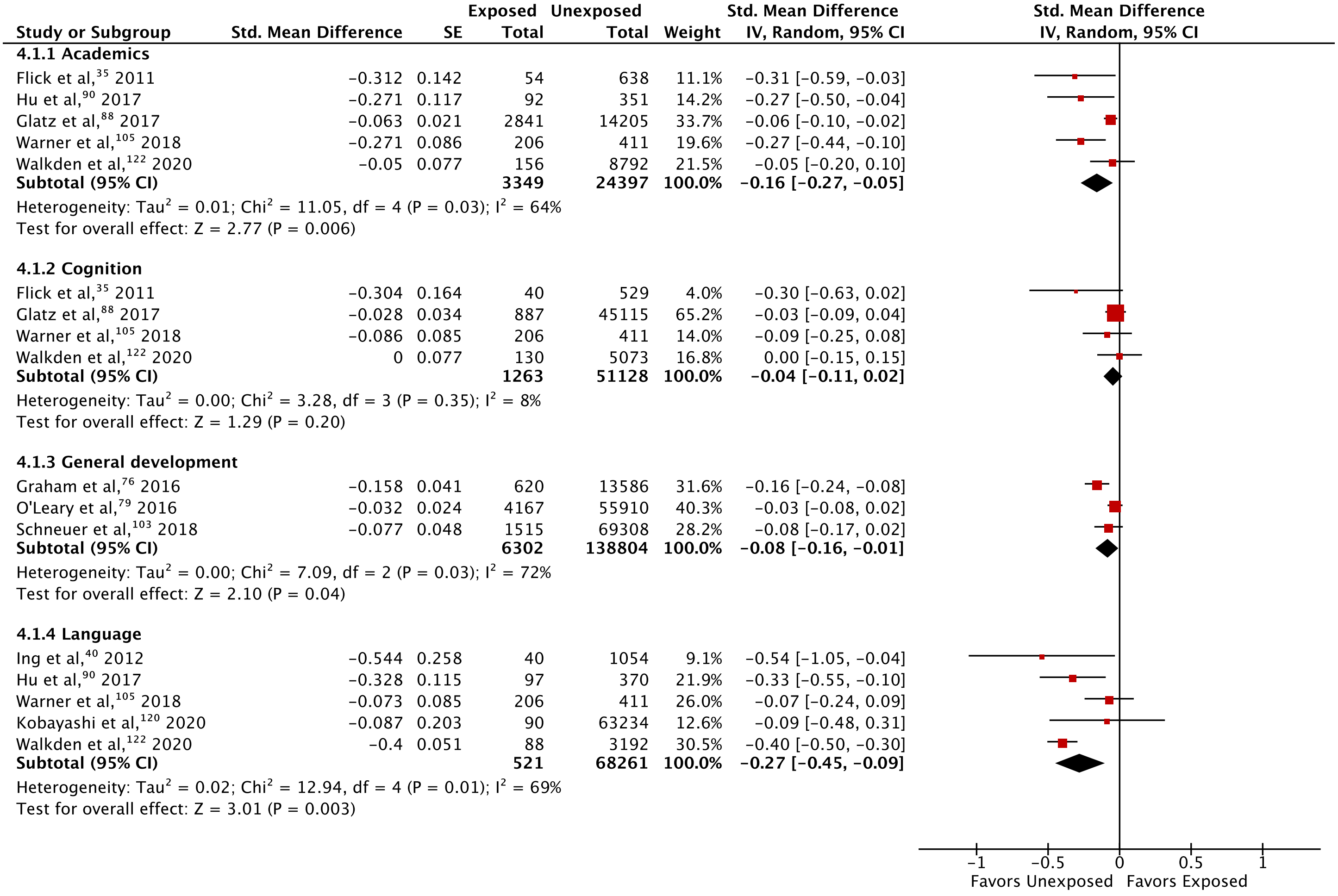

B

Study or Subgroup	log[Hazard Ratio]	SE	Exposed Total	Unexposed Total	Weight	Hazard Ratio IV, Random, 95% CI
**4.2.1 Clinical diagnoses and symptoms – neurodevelopmental disorders**						
Wilder et al,[31] 2009	0.464	0.205	100	4764	31.0%	1.59 [1.06, 2.38]
Flick et al,[35] 2011	0.751	0.264	64	700	18.7%	2.12 [1.26, 3.56]
Sprung et al,[45] 2012	0.668	0.327	64	5007	12.2%	1.95 [1.03, 3.70]
Hu et al,[90] 2017	0.775	0.255	116	463	20.1%	2.17 [1.32, 3.58]
Tsai et al,[104] 2018	0.536	0.269	342	3438	18.0%	1.71 [1.01, 2.90]
**Subtotal (95% CI)**			**686**	**14372**	**100.0%**	**1.86 [1.48, 2.32]**
Heterogeneity: $Tau^2 = 0.00$; $Chi^2 = 1.31$, df = 4 (P = 0.86); $I^2 = 0\%$						
Test for overall effect: Z = 5.41 (P < 0.001)						

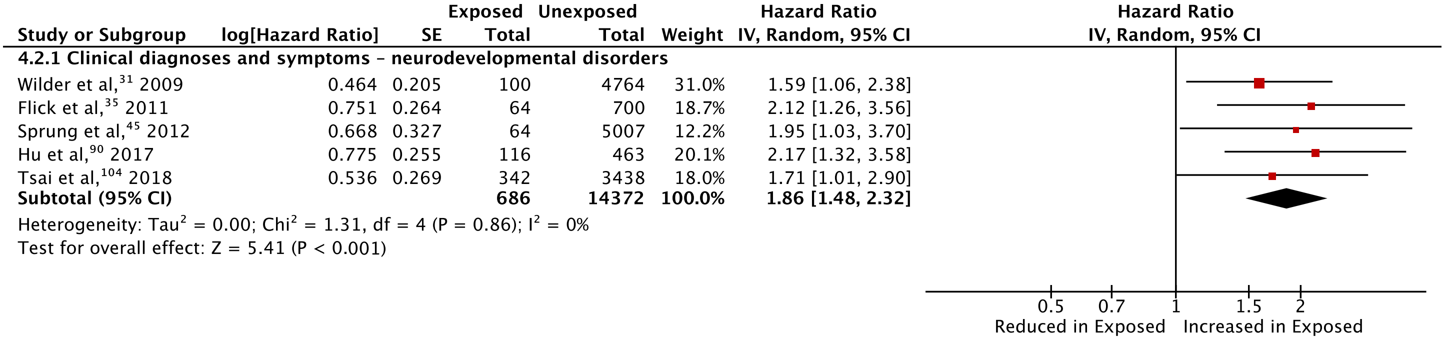

Legend: Panel A: Studies reporting standardized mean differences of domain scores after *Multiple exposure* to surgery and anesthesia. Panel B: Studies reporting hazard ratios for neurodevelopmental disorder diagnoses after *Multiple exposure* to surgery and anesthesia

© 2022 Reighard C et al. *JAMA Network Open.*

eFigure 7. Domain-Specific Meta-analysis of Subdomain Scores and Hazard of ADHD After Multiple Exposure to Surgery and Anesthesia

A

Study or Subgroup	Std. Mean Difference	SE	Exposed Total	Unexposed Total	Weight	Std. Mean Difference IV, Random, 95% CI
**4.3.1 Fine motor function**						
Warner et al,[105] 2018	−0.321	0.086	206	411	37.8%	−0.32 [−0.49, −0.15]
Kobayashi et al,[120] 2020	−0.492	0.143	90	63234	13.7%	−0.49 [−0.77, −0.21]
Walkden et al,[122] 2020	−0.3	0.076	80	3012	48.5%	−0.30 [−0.45, −0.15]
**Subtotal (95% CI)**			**376**	**66657**	**100.0%**	**−0.33 [−0.44, −0.23]**

Heterogeneity: $Tau^2 = 0.00$; $Chi^2 = 1.44$, df = 2 (P = 0.49); $I^2 = 0\%$
Test for overall effect: Z = 6.32 (P < 0.001)

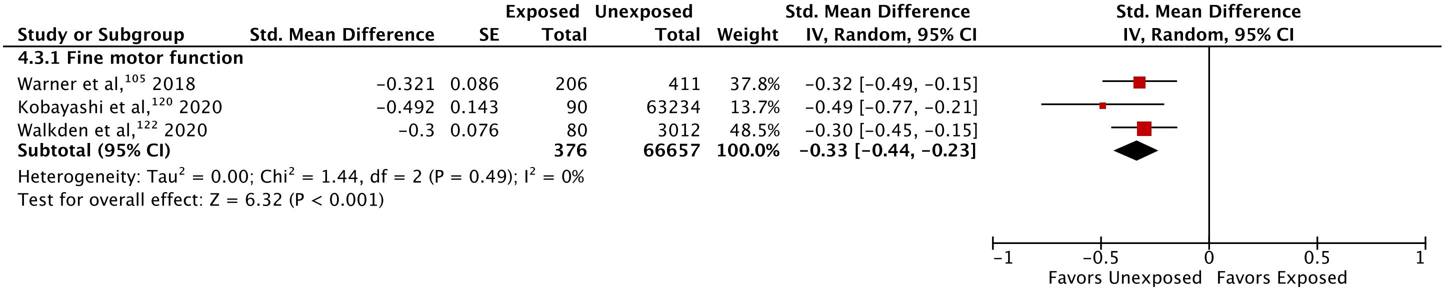

B

Study or Subgroup	log[Hazard Ratio]	SE	Exposed Total	Unexposed Total	Weight	Hazard Ratio IV, Random, 95% CI
**4.4.1 Diagnosis of attention deficit hyperactivity disorder**						
Sprung et al,[45] 2012	0.668	0.327	64	5007	23.7%	1.95 [1.03, 3.70]
Hu et al,[90] 2017	0.952	0.248	116	463	41.2%	2.59 [1.59, 4.21]
Tsai et al,[104] 2018	0.536	0.269	342	3438	35.0%	1.71 [1.01, 2.90]
**Subtotal (95% CI)**			**522**	**8908**	**100.0%**	**2.09 [1.53, 2.86]**

Heterogeneity: $Tau^2 = 0.00$; $Chi^2 = 1.35$, df = 2 (P = 0.51); $I^2 = 0\%$
Test for overall effect: Z = 4.64 (P < 0.001)

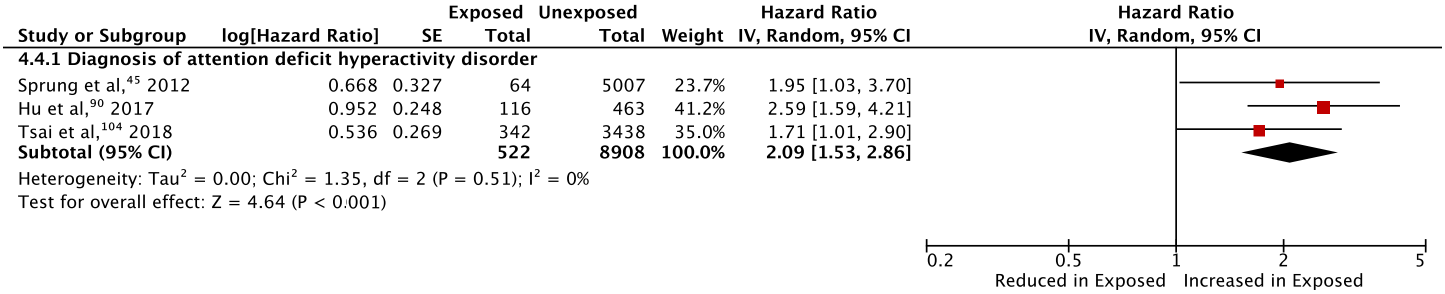

Legend: Panel A: Studies reporting standardized mean differences of subdomains of motor problems after *Multiple exposure* to surgery and anesthesia. Panel B: Studies reporting hazard ratios for ADHD diagnosis after *Multiple exposure* to surgery and anesthesia

© 2022 Reighard C et al. *JAMA Network Open.*

eFigure 8. ROBINS-I Risk of Bias Assessment Figure

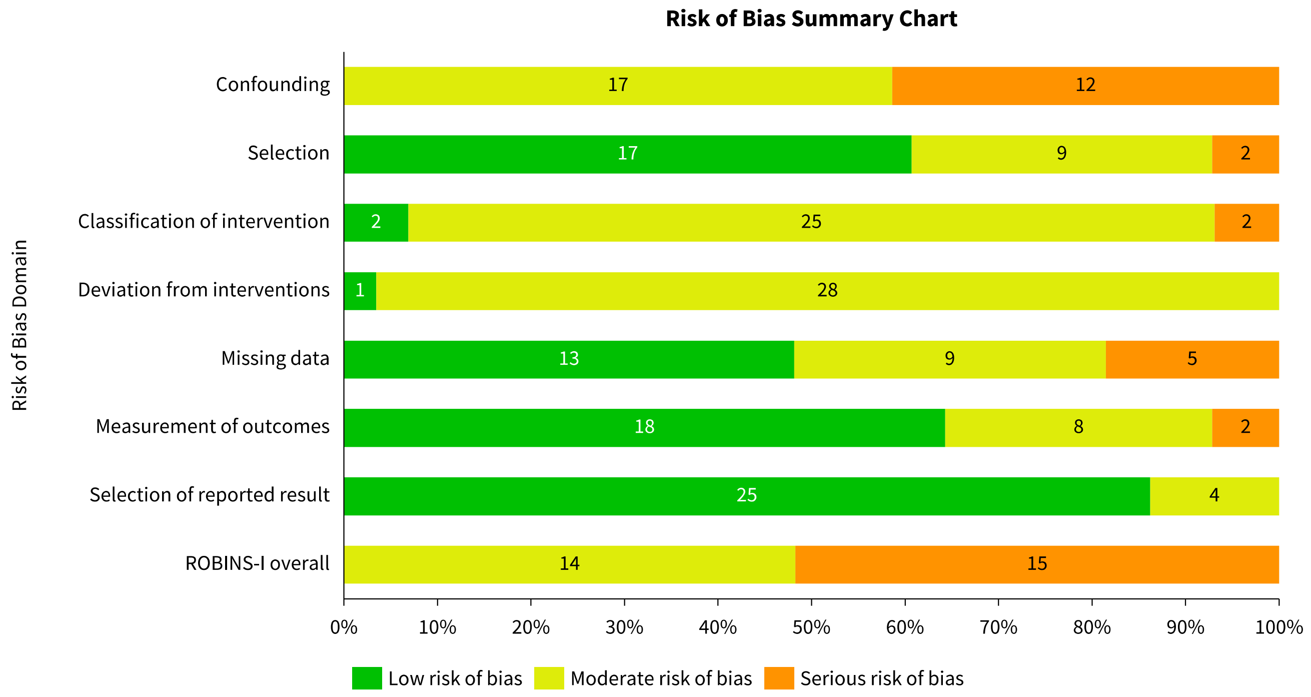

Legend: Summary chart showing number of studies with low, moderate, or serious risk of bias for each of seven domains as well as overall score in ROBINS-I assessment

© 2022 Reighard C et al. *JAMA Network Open.*

eFigure 9. Funnel Plot for Studies of Any Exposure to Surgery and Anesthesia

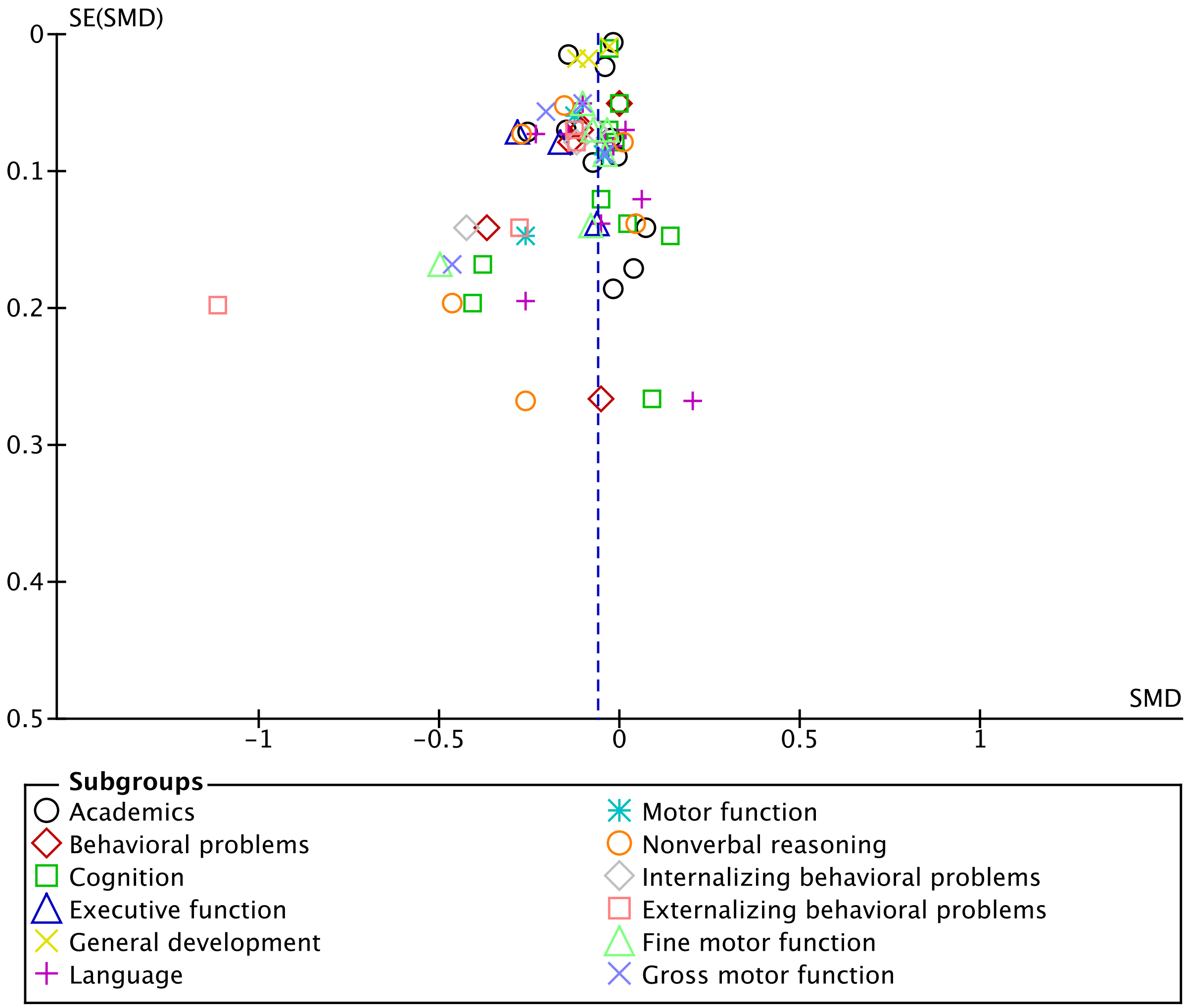

© 2022 Reighard C et al. *JAMA Network Open.*
